# Spectroscopic studies reveal details of substrate-induced conformational changes distant from the active site in isopenicillin N synthase

**DOI:** 10.1016/j.jbc.2022.102249

**Published:** 2022-07-11

**Authors:** Patrick Rabe, Carla C. Walla, Noelle K. Goodyear, Jordan Welsh, Rebecca Southwart, Ian Clifton, James D.S. Linyard, Anthony Tumber, Tim D.W. Claridge, William K. Myers, Christopher J. Schofield

**Affiliations:** 1 Chemistry Research Laboratory, Department of Chemistry and the Ineos Oxford Institute for Antimicrobial Research, University of Oxford, Oxford, United Kingdom; 2 Inorganic Chemistry Laboratory, Department of Chemistry, University of Oxford, Oxford, United Kingdom

**Keywords:** enzyme catalysis, structural dynamics, NMR spectroscopy, isopenicillin N synthase, catalysis, DEER/EPR, 2-oxoglutarate/α-ketoglutarate oxygenases, 2OG, 2-oxoglutarate, ACV, l-δ-(α-aminoadipoyl)-l-cysteinyl-d-valine, BTFA, 3-bromo-1,1,1-trifluoroacetone, CV, column volume, DEER, double electron–electron resonance, DSBH, double-stranded β-helix, EPR, electron paramagnetic resonance, IPN, isopenicillin N, IPNS, isopenicillin N synthase, IPSL, 3-(2-iodoacetamido)-PROXYL spin label, MS, mass spectrometry, MTSL, (1-oxyl-2,2,5,5-tetramethylpyrroline-3-methyl)methanesulfonothioate spin label, NO, nitric oxide, O_2_, dioxygen, PDB, Protein Data Bank, SPE, solid-phase extraction, TCEP, Tris(2-carboxyethyl)phosphine, tr-SFX, time-resolved serial femtosecond crystallography

## Abstract

Isopenicillin N synthase (IPNS) catalyzes formation of the β-lactam and thiazolidine rings of isopenicillin N from its linear tripeptide l-δ-(α-aminoadipoyl)-l-cysteinyl-d-valine (ACV) substrate in an iron- and dioxygen (O_2_)-dependent four-electron oxidation without precedent in current synthetic chemistry. Recent X-ray free-electron laser studies including time-resolved serial femtosecond crystallography show that binding of O_2_ to the IPNS–Fe(II)–ACV complex induces unexpected conformational changes in α-helices on the surface of IPNS, in particular in α3 and α10. However, how substrate binding leads to conformational changes away from the active site is unknown. Here, using detailed ^19^F NMR and electron paramagnetic resonance experiments with labeled IPNS variants, we investigated motions in α3 and α10 induced by binding of ferrous iron, ACV, and the O_2_ analog nitric oxide, using the less mobile α6 for comparison. ^19^F NMR studies were carried out on singly and doubly labeled α3, α6, and α10 variants at different temperatures. In addition, double electron–electron resonance electron paramagnetic resonance analysis was carried out on doubly spin-labeled variants. The combined spectroscopic and crystallographic results reveal that substantial conformational changes in regions of IPNS including α3 and α10 are induced by binding of ACV and nitric oxide. Since IPNS is a member of the structural superfamily of 2-oxoglutarate-dependent oxygenases and related enzymes, related conformational changes may be of general importance in nonheme oxygenase catalysis.

Members of the 2-oxoglutarate (2OG) oxygenase structural superfamily are widespread in nature and have important roles, including in collagen biosynthesis, lipid metabolism, nucleic acid repair, signaling, and antibiotic biosynthesis (1, 2, 3). The importance of these oxygenases in medicine is highlighted by the reaction catalyzed by isopenicillin N synthase (IPNS), an essential enzyme in the biosynthesis of all penicillin and cephalosporin antibiotics (1, 4). IPNS catalyzes the formation of both the β-lactam and thiazolidine rings of isopenicillin N (IPN) from its linear tripeptide l-δ-(α-aminoadipoyl)-l-cysteinyl-d-valine (ACV) substrate in an iron- and dioxygen (O_2_)-dependent four-electron oxidative reaction without precedent in current synthetic chemistry (Fig. 1*A*) (5, 6, 7). The details of the reactions of 2OG oxygenases with O_2_ are of particular interest, in part because some act as hypoxia sensors in humans and other animals (8, 9).

**Figure 1:**
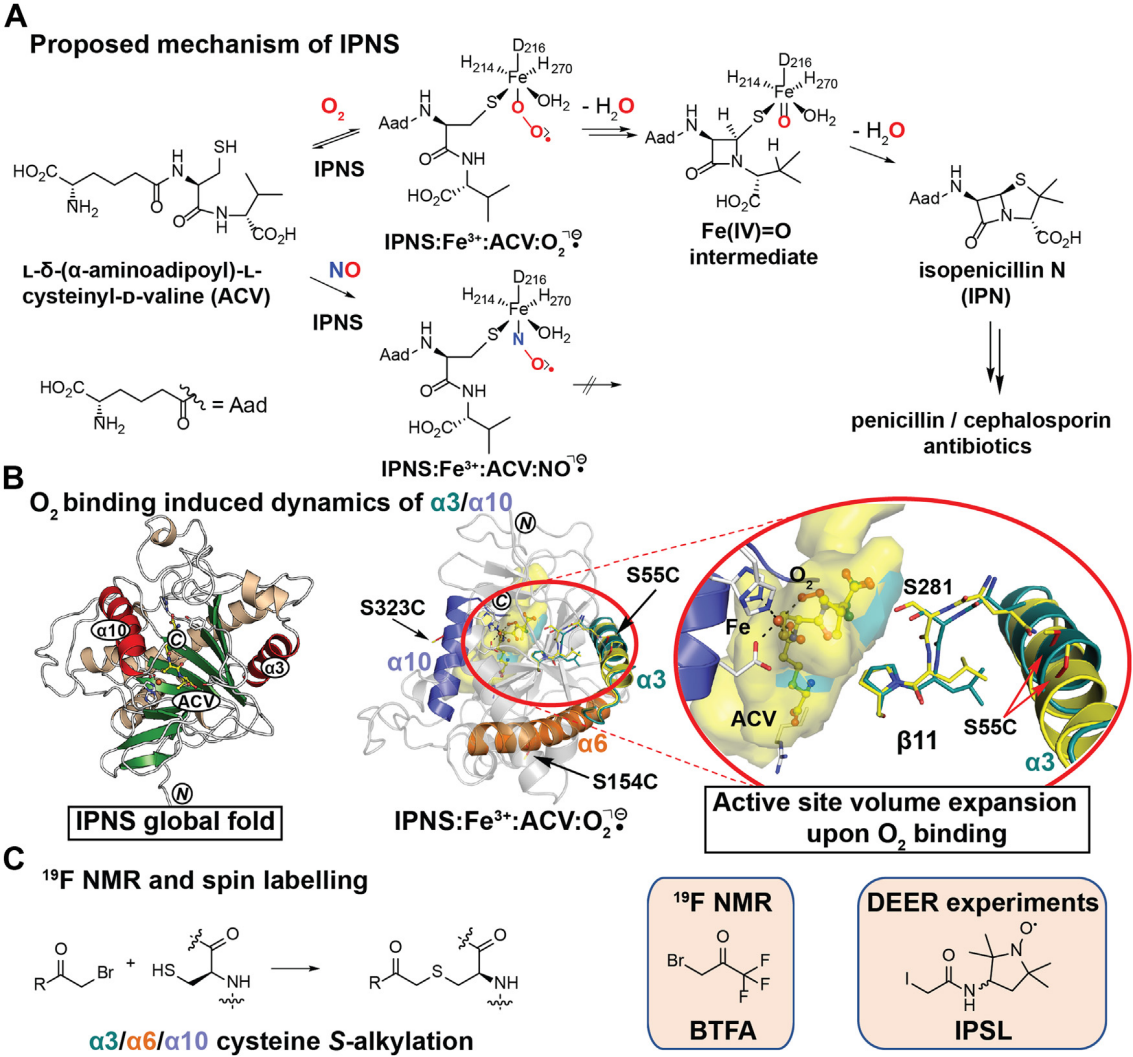
**IPNS-catalyzed penicillin formation showing evidence for O**_**2**_**binding induced rearrangement of helices α3 and α10 and reagents used to introduce labels.***A*, proposed IPNS reaction mechanism showing key intermediates. *B*, *left*, *ribbon view* of IPNS (PDB ID: 1BK0) (39) complexed with Fe (*orange sphere*) and l-δ-(α-aminoadipoyl)-l-cysteinyl-d-valine (ACV, *yellow*). α-helices: *wheat*; β-strands: *green*; and α-helices α3 and α10 that become dynamic on O_2_/NO binding: *red*. *Right*, view from a structure of IPNS–Fe–ACV–O_2_^⸣•−^ (PDB ID: 6ZAP) (21) indicating how O_2_ binding induces dynamic motions of α3 (*yellow*: −O_2_/*teal*: +O_2_) and α10 (*purple*) and (temporary) active site volume expansion (21). To study the extent of dynamics of α3 and α10 in solution, α6 (*orange*) that is predicted to be relatively immobile was investigated. *C*, cys *S*-alkylation with 3-bromo-1,1,1-trifluoroacetone (BTFA, ^19^F NMR experiments) and 3-(2-iodoacetamido)-PROXYL (IPSL, double electron–electron resonance [DEER] experiments). IPNS, isopenicillin N synthase; O_2_, dioxygen; PDB, Protein Data Bank.

There is evidence that conformational changes in catalysis by 2OG-dependent oxygenases are involved in 2OG cosubstrate and substrate binding (10, 11, 12), as supported by NMR and modeling studies (13, 14). Thus, in the case of prolyl hydroxylases, a flexible loop folds to enclose the substrate at the active site (10, 15, 16); flexible loops are also involved in catalysis by the JmjC demethylases/hydroxylases (17) and by 2OG oxygenases acting on small molecules, for example, clavaminic acid synthase (18). There is also evidence that binding of Fe(II)-chelating inhibitors, including human drugs, at the active site of 2OG oxygenases can induce conformational changes (19). These pioneering results have revealed that conformational changes close to the active site can be induced by substrate binding; however, despite evidence from calculations (14, 20), there is missing experimental evidence concerning the roles of dynamic motions extending through large parts of the protein fold during catalysis by 2OG oxygenases and structurally related enzymes including IPNS.

To investigate the extent of structural dynamics that occur during IPNS catalysis, we performed X-ray free-electron laser studies including time-resolved serial femtosecond crystallography (tr-SFX) and X-ray emission spectroscopy on the reaction of IPNS–Fe–ACV crystals with O_2_ (21). This “molecular movie” approach enabled the structural characterization of an Fe(III) superoxide intermediate during *in crystallo* turnover and identified O_2_ binding–induced conformational changes, including of the substrate ACV relating to time-dependent changes in the active site volume. Calculations involving the 2OG-binding pocket of the human 2OG-dependent AlkB homolog 5 (AlkBH5) have led to the prediction of conformational changes that expand the active site to permit catalytically productive substrate binding (13). Unexpectedly, we observed that binding of O_2_, or of its analog nitric oxide (NO), to the active site induces conformational changes in α-helices on the surface of IPNS, in particular in α3 and α10 (Fig. 1*B*) (21). Our initial crystallographic observations were supported by studies employing ^19^F NMR analyses of a ^19^F-labeled IPNS variant produced by alkylation of a cysteine residue on α3 with 3-bromo-1,1,1-trifluoroacetone (BTFA) (21). We now report detailed ^19^F NMR and electron paramagnetic resonance (EPR) solution studies that investigate how binding of Fe(II), ACV, and the O_2_ surrogate NO induce conformational changes in α3 and α10, using the less mobile helix α6 for comparison.

^19^F NMR spectroscopy is useful for measuring changes in protein conformation and dynamics because: (i) ^19^F atoms can be introduced site specifically, (ii) ^19^F atoms do not occur naturally in proteins, and their chemical shifts are sensitive to the local environment (22), (iii) ^19^F atoms typically cause minimal steric changes (the atomic radius of fluorine is close to that of a proton), and (iv) the spin ½ ^19^F nucleus has 100% natural abundance and is a stable nonradioactive isotope (22, 23, 24) enabling analyses at protein concentrations as low as 15 μM, ∼10-fold less than that normally required for ^1^H-detected ^13^C-NMR or ^15^N-NMR studies (25). Since a pioneering report of ^19^F NMR analyses on chymotrypsin complexed with a fluorinated ligand in the 1960s (26), applications of ^19^F NMR spectroscopy have been extended to studies on conformational changes on a range of proteins (25, 27, 28).

EPR spectroscopy is used to study dynamic properties of proteins in solution and can provide information on distances between specific sites. By contrast with NMR (which involves the interaction of an external magnetic field with isotopic nuclei of individual atoms), EPR involves interaction of an external magnetic field with an unpaired electron spin (29). In classical EPR experiments, proteins possessing paramagnetic entities, that is, metal ions or radical cofactors, are analyzed. Advances in molecular biology labeling techniques enable the site-specific incorporation of stable organic radicals at specific locations (30, 31, 32); commonly used nitroxide spin labels are the (1-oxyl-2,2,5,5-tetramethylpyrroline-3-methyl)methanesulfonothioate spin label (MTSL) and the 3-(2-iodoacetamido)-PROXYL spin label (IPSL; Fig. 1*C*) (33, 34), which can be used to study conformational changes and dynamics with labeled proteins (29, 35) and in cells (35). Pulsed EPR experiments employ the four-pulse double electron–electron resonance (DEER) method (a pump-probe sequence with high-power microwave pulses covering two spectral regions) to measure distances between spin centers in the range of 20 to 80 Å (36, 37). By selectively introducing two stable spin centers at specific locations, distance information can be obtained (37).

Here, we describe detailed ^19^F NMR and EPR studies in solution on substrate binding–induced changes in regions of IPNS employing ^19^F and spin-labeled IPNS variants, respectively. Our initial results of ^19^F-labeled IPNS (21) are extended to detailed ^19^F NMR and EPR studies on α3, α10, and the less dynamic α6. Temperature-dependent experiments on labeled IPNS variants highlight the extent of conformational changes in α-helices on the IPNS surface. We used DEER experiments to evaluate distance information between two stable spin centers, introduced by site-directed spin labeling of the α-helices. The NMR and EPR results obtained in solution are compared with reported crystal structures of IPNS and new structures of IPNS covalently modified with a spin label, obtained under anaerobic and NO-exposed conditions. The combined spectroscopic and crystallographic results support a role for motions extending throughout substantial portions of the protein fold, in regions including α3 and α10, during IPNS catalysis.

## Results

IPNS contains a distorted double-stranded β-helix (DSBH) core fold (cupin or “jelly-roll” fold, Fig. 1*B*), which is characteristic of the 2OG oxygenase structural superfamily (1, 7). Several α-helices are located, at least in part, on the exterior of the DSBH fold, that is, α3, α4, α6, α9, and α10. Recently reported X-ray free-electron laser studies have provided evidence that, along with certain other regions, α3 and α10 play roles in IPNS catalysis (21). The C-terminal region including α10 is likely involved in ACV substrate binding and IPN product release, and α3 is involved in conformational changes induced by O_2_ binding (6, 7, 21). However, for the other α-helices including α6, which is located on the exterior of the DSBH, no direct roles in catalysis have been demonstrated, and tr-SFX studies did not provide evidence for their involvement in substantial conformational changes in conversion of active site–complexed ACV to IPN (21).

To use ^19^F NMR and EPR to investigate the relative extent of changes in α3, α6, and α10 induced by Fe(II), ACV, and O_2_ binding in solution, the latter employing NO as an O_2_ analog, Cys-substituted IPNS variants suitable for labeling were produced by site-directed mutagenesis followed by recombinant protein production in *Escherichia coli* and purification. Variants produced included singly labeled IPNS^S55C^ (located on α3), IPNS^S154C^ (α6), and IPNS^S323C^ (α10) variants as well as the doubly labeled IPNS^S55C+S154C^ (α3/α6), IPNS^S154C+S323C^ (α6/α10), and IPNS^S55C+S323C^ (α3/α10) variants. Following substitution of the serine residues in ⍺3, α6, and α10 for a Cys-residue and introduction of a fluorine label (with BTFA, Fig. 1*C*) or a spin label (with IPSL, Fig. 1*C*) by S-alkylation, we monitored changes by ^19^F NMR or EPR spectroscopy, respectively (note that IPSL is racemic so likely reacts to give a mixture of epimers). The purities of the labeled proteins were shown by SDS-PAGE (>95%) and mass spectrometry (MS) analyses. The (un)labeled IPNS variants were shown to be active by assays employing solid-phase extraction (SPE) coupled with MS; similar activities to WT IPNS were observed (Fig. S1). The *K*_*M*_ values for the ^19^F-labeled IPNS^S55C^ (*K*_*M*_ = 196 ± 19 μM, pH 8) and IPNS^S154C^ (*K*_*M*_ = 236 μM, pH 8) were similar to that of WT IPNS (*K*_*M*_ = 206 μM, pH 7.94) (38), though that for ^19^F IPNS^S323C^ (*K*_*M*_ = 296 ± 46 μM, pH 8) was slightly higher.

### Titrations of catalytic compounds have significant effects on ^19^F NMR shifts of α3 and α10

Initial studies on the ^19^F-labeled IPNS^S55C^ (α3) variant revealed that sequential addition of Fe(II), ACV, then NO to *apo*-IPNS resulted in moderate changes in the chemical shifts in the ^19^F spectra. By contrast, addition of NO to the IPNS^S55C^–Fe or IPNS^S55C^–Cd–ACV complexes revealed no apparent shifts in the analogous IPNS^S55C^–Fe or IPNS^S55C^–Cd–ACV ^19^F signals, respectively (21). We extended analyses of the ^19^F-labeled IPNS^S55C^ to titrations with ZnSO_4_ (Fig. S2) and ACV; the results reveal distinct ^19^F chemical shifts for the IPNS–Zn and IPNS–Zn–ACV complexes. By contrast with the result for the IPNS^S55C^–Fe–ACV complex, no changes in chemical shift were observed on addition of NO to the IPNS^S55C^–Zn–ACV complex, as anticipated on the results of the IPNS^S55C^–Cd–ACV complex (21). We also analyzed whether the order of Fe(II) or ACV addition to IPNS^S55C^ has an impact on the ^19^F NMR spectrum; comparison of the IPNS^S55C^–Fe(II)–ACV complex obtained after stepwise addition of Fe(II) followed by ACV or ACV followed by Fe(II) to IPNS^S55C^ showed no differences (Fig. S3).

**Figure 2:**
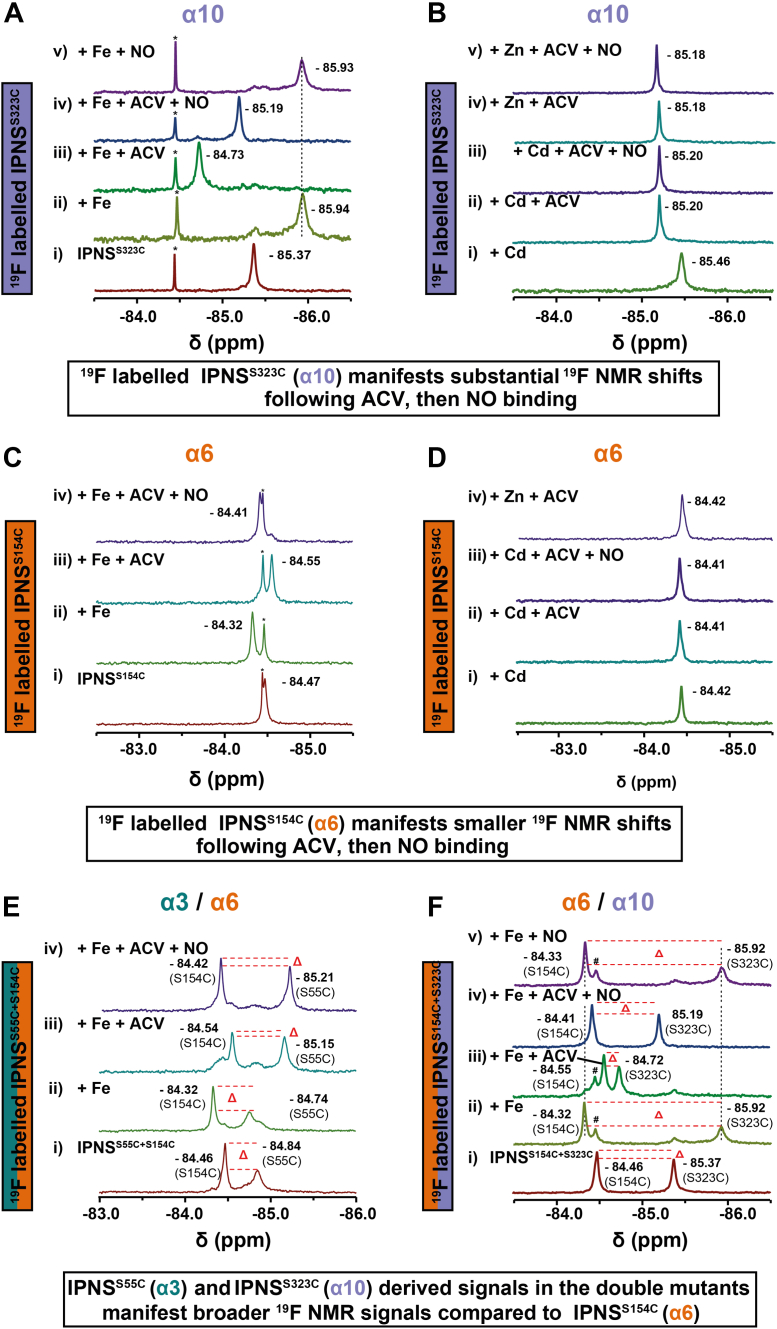
^**19**^**F NMR spectra of titrations of**^**19**^**F-labeled IPNS variants with ACV and Fe(II), Cd(II), or Zn(II), with/without NO exposure imply α3 and α10 undergo more substantial conformational changes than α6.***A*, (i) IPNS^S323C^ (120 μM) and CF_3_CO_2_H (100 μM) in Tris-d_11_ (25 mM, pH 7.5)/D_2_O (50 μl, 10% [v/v]); (ii) IPNS^S323C^ with excess Fe(II) (5 equivalents, 600 μM); (iii) IPNS^S323C^–Fe(II) with excess ACV (64 equivalents, 7.68 mM); (iv) IPNS^S323C^–Fe(II)–ACV + NO (1000 ppm in N_2_, 45 min); and (v) IPNS^S323C^–Fe(II) (71 μM) + NO (1000 ppm in N_2_, 45 min). The ^19^F signal indicated by ∗ is due to residual copurifying 3-bromo-1,1,1-trifluoropropane-2,2-diol produced during ^19^F labeling. *B*, (i) IPNS^S323C^ with excess Cd(II) (5 equivalents, 600 μM); (ii) IPNS^S323C^–Cd(II) with excess ACV (64 equivalents, 7.68 mM); (iii) IPNS^S323C^–Cd(II)–ACV + NO (1000 ppm in N_2_, 35 min); (iv) IPNS^S323C^–Zn(II) with excess ACV (64 equivalents, 7.68 mM); (v) IPNS^S323C^–Zn(II)–ACV + NO (1000 ppm in N_2_, 60 min). *C*, (i) IPNS^S154C^ (120 μM) and CF_3_CO_2_H (100 μM) in Tris-d_11_ (25 mM, pH 7.5) and D_2_O (50 μl, 10% [v/v]); (ii) IPNS^S154C^ with excess Fe(II) (5 equivalents, 600 μM); (iii) IPNS^S154C^–Fe(II) with excess ACV (64 equivalents, 7.68 mM); and (iv) IPNS^S154C^–Fe(II)–ACV + NO (1000 ppm in N_2_, 45 min). *D*, (i) IPNS^S154C^ with excess Cd(II) (5 equivalents, 600 μM); (ii) IPNS^S154C^–Cd(II) with excess ACV (64 equivalents, 7.68 mM); (iii) IPNS^S154C^–Cd(II)–ACV + NO (1000 ppm in N_2_, 40 min) and IPNS^S154C^–Zn(II) with excess ACV (64 equivalents, 7.68 mM). *E*, (i) IPNS^S55C+S154C^ (120 μM) and CF_3_CO_2_H (100 μM) in Tris-d_11_ (25 mM, pH 7.5) and D_2_O (50 μl, 10% [v/v]); (ii) IPNS^S55C+S154C^ with excess Fe(II) (5 equivalents, 600 μM); (iii) IPNS^S55C+S154C^–Fe(II) with excess ACV (64 equivalents, 7.68 mM); and (iv) IPNS^S55C+S154C^–Fe(II)–ACV + NO (1000 ppm in N_2_, 45 min). *F*, (i) IPNS^S154C+S323C^ (120 μM) and CF_3_CO_2_H (100 μM) in Tris-d_11_ (25 mM, pH 7.5) and D_2_O (50 μl, 10% [v/v]); (ii) IPNS^S154C+S323C^ with excess Fe(II) (5 equivalents, 600 μM); (iii) IPNS^S154C+S323C^–Fe(II) with excess ACV (64 equivalents, 7.68 mM); (iv) IPNS^S154C+S323C^–Fe(II)–ACV + NO (1000 ppm in N_2_, 40 min) and IPNS^S154C+S323C^–Fe(II) + NO (1000 ppm in N_2_, 45 min). Note: *Red dashed lines* indicate peak height differences. The ^19^F signal indicated by # is presumably a second conformation as supported by diffusion experiment. The full set of NMR analyses is given in Figs. S2–S8. ACV, l-δ-(α-aminoadipoyl)-l-cysteinyl-d-valine; IPNS, isopenicillin N synthase; NO, nitric oxide.

We then focused our analyses on the ^19^F-labeled IPNS^S323C^ (α10) variant. Titrations of FeSO_4_ with IPNS^S323C^ in an anaerobic environment, followed by addition of ACV, revealed distinct ^19^F NMR signals for the IPNS^S323C^–Fe and IPNS^S323C^–Fe–ACV complexes (Figs. 2*A* and S4). Addition of excess NO (1000 ppm in nitrogen) to the anaerobic IPNS^S323C^–Fe–ACV complex resulted in a substantial change in the chemical shift of the IPNS^S323C^ ^19^F signal. By contrast, no such shift was observed by exposure of IPNS^S323C^–Fe to excess NO, implying that NO binding–induced rearrangement of α10 only occurs in the presence of ACV. These observations with IPNS^S55C^ and IPNS^S323C^ are in agreement with the proposed IPNS mechanism involving ordered sequential binding of ACV and then O_2_ and reported crystallographic studies showing conformational changes in α3 and α10 induced by active-site binding of NO and O_2_ (21). Studies with the catalytically inactive Cd(II) and Zn(II) metal analogs in the presence of ACV were consistent with a lack of NO binding to the active-site metal ions in the IPNS–Cd(II)–ACV or IPNS–Zn(II)–ACV complexes in solution (Figs. 2*B* and S4). One interesting observation was that the addition of Zn(II), but not Cd(II), results in a particularly broad peak/peaks in the absence of ACV for both IPNS^S55C^ and IPNS^S323C^ but not IPNS^S154C^. This broadening was quantified by comparison of peak heights/broadening by full width at half maximum analysis with IPNS^S154C^–Cd (20.8 Hz) and IPNS^S154C^–Zn (23.4 Hz) compared with IPNS^S55C^–Cd (63.5 Hz), IPNS^S55C^–Zn (125.2 Hz), IPNS^S323C^–Cd (39.4 Hz), and IPNS^S323C^–Zn (114.3 Hz) (Figs. S2, S4 and S5). Notably, the addition of ACV to the IPNS^S55C^–Zn and IPNS^S323C^–Zn complexes resulted in restoration of relatively sharp peaks for both variants.

^19^F NMR measurements on titrations of IPNS^S154C^ (α6) with Fe(II), ACV, and NO were then performed. S154 is located on α6, which was not observed to undergo substantial movement during catalysis by tr-SFX (21). The ^19^F NMR results reveal distinct shifts in the ^19^F NMR spectra (Fig. 2*C*) following sequential addition of Fe(II), ACV, and then NO. However, compared with the results for the α3- and α10-labeled variants, where intermediate and substantial changes in chemical shifts were observed, respectively, with the IPNS^S154C^ (α6)-labeled variant, only minor changes in chemical shifts were observed for the individual ^19^F NMR signals with IPNS^S154C^, consistent with a less dynamic nature for α6 helix compared with α3 and α10 (Figs. 2*A* and S5). Interestingly, as noted previously, the addition of Zn(II) did not result in a broad peak in the case of IPNS^S154C^, contrasting with the observations for IPNS^S55C^ and IPNS^S323C^. The combined NMR and tr-SFX studies support the proposal that the substantial changes in chemical shifts observed on ACV and then NO addition to the IPNS–Fe(II) complex positively correlate with the conformational changes in α3 and α10 observed on binding of O_2_ (and NO) to the IPNS active site.

Studies with the doubly ^19^F-labeled IPNS^S55C+S154C^ (Figs. 2*E* and S6) and IPNS^S154C+S323C^ (Figs. 2*F* and S7) variants support the observations with the individually labeled IPNS variants, that is, they reveal very similar shifts for the corresponding ^19^F NMR signals compared with those obtained for the singly labeled IPNS variants. Relatively broad peaks for the corresponding ^19^F NMR signals of IPNS^S55C^ and IPNS^S323C^ in the doubly labeled variants IPNS^S55C+S154C^ and IPNS^S154C+S323C^ were observed for the *apo* complexes and in the presence of Fe and ACV (Figs. 2, *E* and *F*, indicated by *red dashed lines*). By contrast, the corresponding ^19^F signals for IPNS^S154C^ in the *apo*, Fe-, and ACV-bound complexes of IPNS^S55C+S154C^ and IPNS^S154C+S323C^ were relatively sharp (Fig. 2, *E* and *F*), as observed in the singly labeled IPNS^S154C^ spectra. Notably, on addition of NO to all the α3, α6, or α10 ^19^F-labeled IPNS–Fe–ACV complexes, peak sharpening was observed on formation of the IPNS–Fe–ACV–NO complexes.

Collectively, these experiments imply that α3 and α10 are more conformationally flexible/adopt more conformations then α6, consistent with the time-resolved crystallographic studies (21). They provide evidence that binding of Fe(II), ACV, and NO induces conformational changes in α3 and α10 implying that relatively small changes at the active site can affect the conformation of the protein surface.

### Temperature-dependent ^19^F NMR studies on the dynamics of α3 and α10

To further investigate how the addition of Fe(II), Zn(II), or Cd(II), ACV, and NO effect the ^19^F NMR spectra, we then carried out variable temperature studies. The ^19^F NMR spectra of the singly labeled *apo*-IPNS variants (Figs. S9*A*, S12*A* and S15*A*) implied full decomposition of folded IPNS after heating to 313 K (rerecording the ^19^F NMR spectrum at 298 K after heating to 313 K revealed loss of the ^19^F NMR signal). By contrast in the presence of Fe(II), all the IPNS–Fe variant complexes were more stable (Figs. S9*B*, S12*B* and S15*B*); analysis of spectra after heating of the sample to 313 K in the presence of Fe(II) implied only minor decomposition for IPNS^S55C^ (α3) and IPNS^S323C^ (α10); no apparent decomposition was observed for IPNS^S154C^ (α6). This observation implies that irreversible decomposition of the sample (*e.g.*, by polymerization/irreversible unfolding) is unlikely to be a reason for the peak changes observed in the Fe complexes after heating to 313 K.

**Figure 3:**
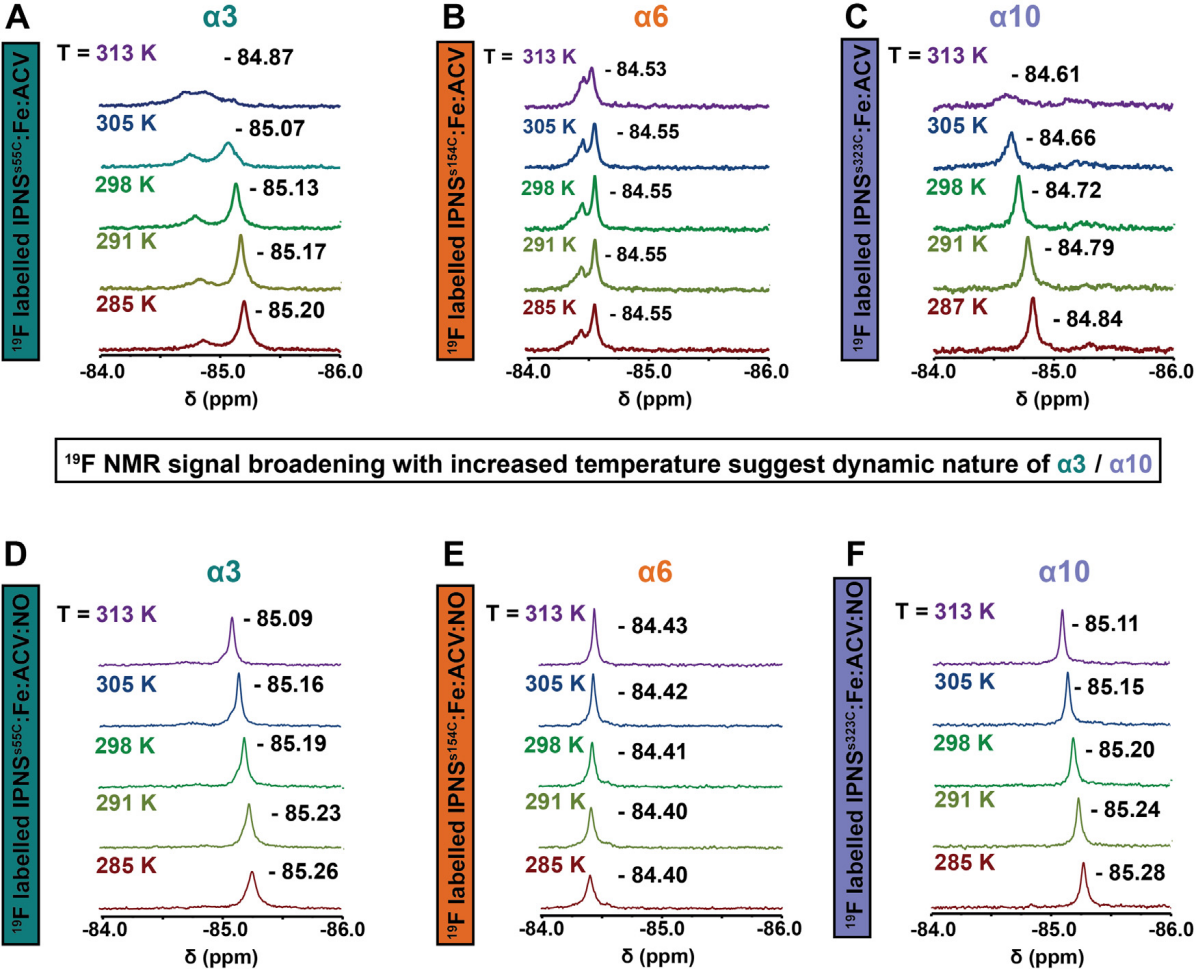
^**19**^**F NMR spectra of**^**19**^**F-labeled IPNS variants with Fe(II) and ACV** ± **NO at different temperatures.***A*, IPNS^S55C^–Fe–ACV (IPNS 120 μM; Fe(II) 5 equivalents, 600 μM; ACV 64 equivalents, 7.68 mM) and CF_3_CO_2_H (100 μM) in Tris-d_11_ (25 mM, pH 7.5) and D_2_O (50 μl, 10% [v/v]) at 285 K (*red*), 291 K (*olive green*), 298 K (*green*), 305 K (*blue*), and 313 K (*purple*). *B*, IPNS^S154C^–Fe–ACV at 285 K (*red*), 291 K (*olive green*), 298 K (*green*), 305 K (*blue*), and 313 K (*purple*). The minor ^19^F signal likely corresponds to a second conformation of α6 as supported by diffusion experiment. *C*, IPNS^S323C^–Fe–ACV at 285 K (*red*), 291 K (*olive green*), 298 K (*green*), 305 K (*blue*), and 313 K (*purple*). Note: ^19^F NMR spectra reveal significant peak broadening of the ^19^F signals for ^19^F-labeled IPNS^S55C^ (α3) and IPNS^S323C^ (α10), whereas the ^19^F spectra of IPNS^S154C^ (α6) show only minor peak broadening. *D*, IPNS^S55C^–Fe–ACV–NO (IPNS 120 μM; Fe(II) 5 equivalents, 600 μM; ACV 64 equivalents, 7.68 mM, NO exposure (1000 ppm in N_2_, >30 min)) and CF_3_CO_2_H (100 μM) in Tris-d_11_ (25 mM, pH 7.5) and D_2_O (50 μl, 10% [v/v]) at 285 K (*red*), 291 K (*olive green*), 298 K (*green*), 305 K (*blue*), and 313 K (*purple*). *E*, IPNS^S154C^–Fe–ACV–NO at 285 K (*red*), 291 K (*olive green*), 298 K (*green*), 305 K (*blue*), and 313 K (*purple*). *F*, IPNS^S323C^–Fe–ACV–NO at 285 K (*red*), 291 K (*olive green*), 298 K (*green*), 305 K (*blue*), and 313 K (*purple*). ACV, l-δ-(α-aminoadipoyl)-l-cysteinyl-d-valine; IPNS, isopenicillin N synthase; NO, nitric oxide.

Comparison of the peak shapes revealed sharpening of the ^19^F NMR signals for all IPNS^S55C^ (α3), IPNS^S154C^ (α6), and IPNS^S323C^ (α10) in the presence of Fe(II) with increasing temperatures (Figs. S9, S12 and S15). By contrast, addition of ACV to the IPNS–Fe variant complexes manifested an opposite trend with increasing temperature. Whereas IPNS^S154C^–Fe (α6) showed minor broadening effects on ACV binding with increased temperature (Figs. 3*B* and S13*A*), the data for the IPNS^S55C^–Fe (α3) and IPNS^S323C^–Fe (α10) complexes in the presence of ACV revealed very substantial broadening of the ^19^F NMR signal at 313 K (Figs. 3, *A* and *C*, S10*A* and S16*A*). The minor signal in the ^19^F NMR spectrum of IPNS^S55C^–Fe–ACV is likely due to *apo* IPNS^S55C^ or IPNS^S55C^–Fe, because of incomplete ACV binding (Fig. S10*A*). By contrast, the additional signal in the ^19^F NMR spectrum of IPNS^S154C^–Fe–ACV might reflect a second conformation of α6 given that it displayed properties of a macromolecule as supported by diffusion experiments, which exclude the possibility of the signal arising because of hydrolyzed BTFA (Figs. S18–S20).

Analysis of changes in the chemical shifts in the ^19^F NMR spectra of IPNS^S55C^ ± Fe ± ACV ± NO (Figs. S9–S11) and IPNS^S323C^ ± Fe ± ACV ± NO (Figs. S15–S17) with increasing temperature shows deshielding of the ^19^F NMR signals in these two IPNS variants. By contrast, the signals of the IPNS^S154C^ ± Fe ± ACV ± NO complexes (Figs. S12–S14) showed only a small shielding of chemical shifts as a function of temperature. Overall, the observations of broadening in the IPNS^S55C^–Fe–ACV (α3, Fig. 3*A*) and IPNS^S323C^–Fe–ACV (α10, Fig. 3*C*), but not IPNS^S154C^–Fe–ACV (α6, Fig. 3*B*), spectra as a function of temperature support the proposed more substantial conformational changes in α3 and α10 compared with α6.

By contrast with the studies for IPNS–Fe–ACV where ^19^F peak broadening as a function of increasing temperature was observed, peak sharpening was observed in temperature-dependent studies of the catalytically inactive IPNS–Zn–ACV and IPNS–Cd–ACV complexes in the absence of NO (Figs. S11, S14 and S17). Although the precise reasons for the difference in behavior between the catalytically active Fe(II) and inactive Zn(II)/Cd(II) complexes are unclear, these results further reveal how subtle differences in active site coordination chemistry of IPNS can impact on relatively remote structural regions.

### DEER experiments

We used the IPNS cysteine double mutants to introduce stable spin labels to perform DEER experiments to obtain distance information on substrate binding. We performed labeling studies with three commercially available spin labels, that is, IPSL, 3-maleimido-PROXYL, and MTSL. Protein MS analysis revealed that only labeling with racemic 3-(2-iodoacetamido)-PROXYL (Fig. 1*B*) resulted in sufficiently pure (>90%) double-labeled IPNS^S55C+S154C^ (α3/α6), IPNS^S154C+S323C^ (α6/α10), and IPNS^S55C+S323C^ (α3/α10) variants, likely as a mixture of epimers, by S-alkylation of the introduced sulphur residues; reaction with the other spin labels led to multiple nonspecific labeling as observed by MS.

**Figure 4:**
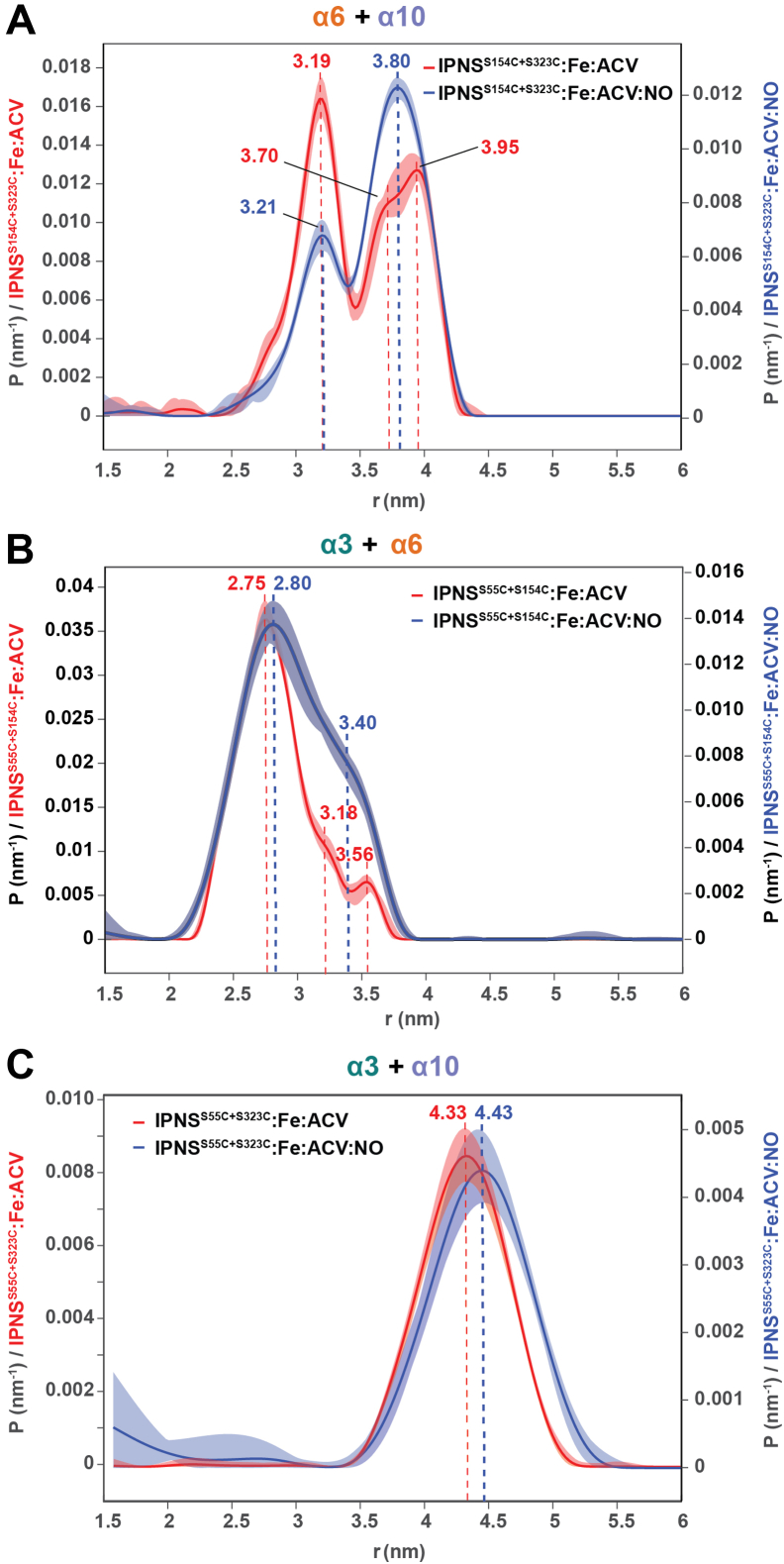
**DEER experiments with doubly spin-labeled IPNS variants in complex with Fe and ACV under anaerobic and NO-exposed conditions.** Samples of spin-labeled IPNS–Fe–ACV (IPNS 150 μM; Fe(II) 0.97 equivalents, 145 μM; ACV 32 equivalents, 4.80 mM in EPR buffer (Tris 25 mM, 200 mM NaCl, in D_2_O, pH 8.0); and 30% [v/v] glycerol-*d*_8_) were prepared and exposed to NO (1000 ppm in N_2_, 30 min) to obtain spin-labeled IPNS–Fe–ACV–NO. *A*, comparison of distance distributions derived from doubly spin-labeled IPNS^S154C+S323C^–Fe–ACV (*red*) and IPNS^S154C+S323C^–Fe–ACV–NO (*blue*) revealing an increase of the species with a longer distance distribution upon NO binding. *B*, comparison of distance distributions derived from doubly spin-labeled IPNS^S55C+S154C^–Fe–ACV (*red*) and IPNS^S55C+S154C^–Fe–ACV–NO (*blue*) revealing an increase of the species with a longer distance distribution upon NO binding. *C*, distance distributions derived from experimental data of doubly spin-labeled IPNS^S55C+S323C^–Fe–ACV ± NO revealing one major distance distribution of 4.33 nm (anaerobic) and 4.43 nm (+NO). ACV, l-δ-(α-aminoadipoyl)-l-cysteinyl-d-valine; DEER, double electron–electron resonance; IPNS, isopenicillin N synthase; NO, nitric oxide.

After optimization of the EPR samples, we performed DEER experiments with the double-labeled IPNS variants. Analysis of the distance distributions between the two spin labels of IPNS^S154C+S323C^–Fe–ACV (α6/α10) revealed a major distance distribution with a maximum of 3.2 nm; two other smaller distance distributions with local maxima at 3.7 and 4.0 nm were also observed under anaerobic conditions. Exposure of IPNS^S154C+S323C^–Fe–ACV (α6/α10) to NO revealed a significant change in the distribution maxima to the longer distances with a distance distribution maximum at 3.8 nm and a smaller local maximum at 3.2 nm (Figs. 4*A* and S21). Analysis of the distance distributions for IPNS^S55C+S154C^–Fe–ACV under anaerobic conditions revealed a main distance distribution of 2.8 nm and a small second local maximum of 3.6 nm. NO exposure of IPNS^S55C+S154C^–Fe–ACV revealed a major distance distribution of 2.8 nm similar to the anaerobic IPNS^S55C+S154C^–Fe–ACV complex and an increase of a species with longer distance distribution. The second distance distribution with a maximum of 3.4 nm is shorter than under anaerobic conditions (3.6 nm), likely because of movement of α3, although an altering of conformational (or less likely, epimeric) populations cannot be fully excluded (Figs. 4*B* and S22). Analysis of the distance distribution of IPNS^S55C+S323C^–Fe–ACV revealed a single distance distribution with a maximum 4.3 nm; exposure of IPNS^S55C+S323C^–Fe–ACV to NO suggested a small increase in this distance, by 0.1 to 4.4 nm (Figs. 4*C* and S23).

### Crystallographic analysis of spin-labeled IPNS^S55C^ complexes in the absence and presence of NO

To investigate whether the presence of the 3-acetamido-PROXYL spin label necessary for the DEER experiments influences conformational changes involving α3 and α10, we carried out crystallographic studies. Previous attempts to crystallize the ^19^F-labeled IPNS^S55C^–Fe–ACV variant revealed covalent modification of C55 with a 1,1,1-trifluoroacetonyl residue (Protein Data Bank [PDB] ID: 6ZAM) (21). We were able to crystallize the anaerobic spin-labeled IPNS^S55C^–Fe–ACV complex and exposed some of these single crystals to NO. After cryocooling and synchrotron data collection, several single crystal datasets of spin-labeled IPNS^S55C^–Fe–ACV and IPNS^S55C^–Fe–ACV–NO complexes under the same conditions were obtained.

Analysis of the anaerobic structure of the spin-labeled IPNS^S55C^–Fe–ACV complex (PDB ID: 7PSW; 1.21 Å resolution) reveals a square pyramidal Fe in the active site ligated *via* H214, D216, and H270, the ACV thiolate, and a water molecule with a vacant coordination site. The active site and global structure appear to be near identical with that previously obtained for anaerobic complexes of IPNS–Fe–ACV (PDB ID: 1BK0 (39) (cryo), C-α RMSD: 0.097 Å; PDB ID: 6Y0O (40) [room temperature SSX], C-α RMSD: 0.286 Å; and PDB ID: 6ZAE (21) [room temperature SFX], C-α RMSD: 0.233 Å). Careful analysis of the electron density close to C55 revealed several positive density features reflecting covalent modification by IPSL. We attempted to model and iteratively fit the 3-(acetomido)-PROXYL spin label on C55 in the anaerobic complex. However, confident modeling of the covalent modification was not possible, despite multiple modeling attempts with different conformations of the 3-(acetomido)-PROXYL spin label. Hence, although clear positive electron density after calculation of m*F*_o_-D*F*_c_ maps was apparent close to C55 (Fig. S24*D*), the coordinates for the 3-(acetomido)-PROXYL spin label were not included in the model used for PDB deposition.

**Figure 5:**
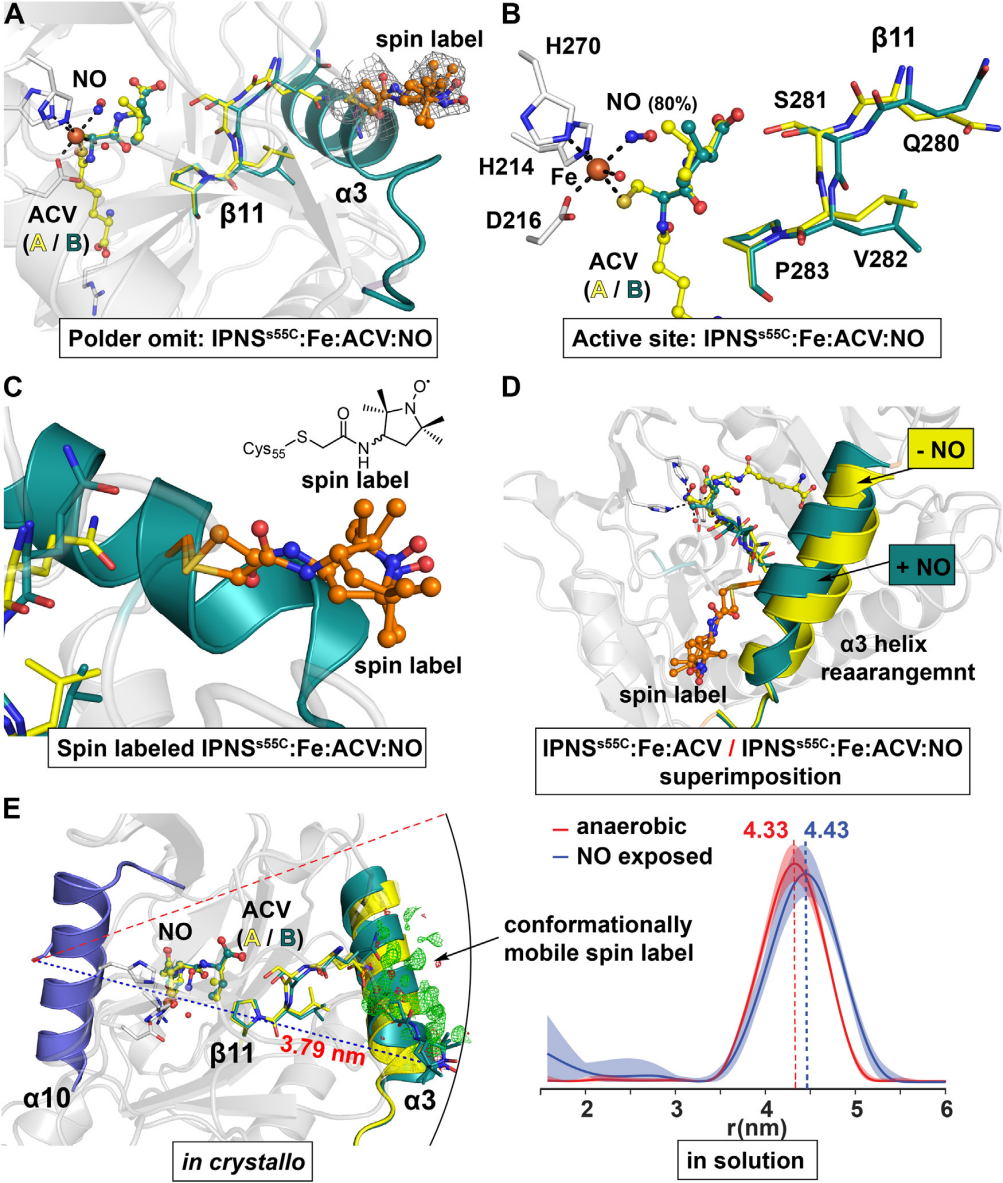
**Views from crystal structures of spin-labeled IPNS**^**S55C**^**–Fe–ACV (PDB ID:****7PSW****) and spin-labeled IPNS**^**S55C**^**–Fe–ACV–NO (PDB ID:****7POY****) complexes revealing conformational rearrangement of α3 on NO binding.***A*, Polder omit map of the spin-labeled IPNS^S55C^–Fe–ACV–NO complex (PDB ID: 7POY, 1.75 Å resolution, 3.0 σ contour level) revealing the 3-acetomido-PROXYL spin label covalently bound to C55 and partial binding of NO (80%). The latter is reflected in the observation of two ACV conformations: (*A*) under anaerobic conditions (20%, *yellow*) and (*B*) after NO binding (80%). *B*, active-site view of spin-labeled IPNS^S55C^–Fe–ACV–NO revealing incomplete NO binding (80%) to Fe (coordinated *via* H214, D216, H270, the ACV thiolate, and a water). NO binding induces rearrangement of the ACV (*teal*) and, remotely, S281, adjacent to Q280, V282, and P283 (part of sheet β11). *C*, close-up of the 3-acetomido-PROXYL spin label bound to C55, revealing two major conformations. *D*, superimposed views of spin-labeled IPNS^S55C^–Fe–ACV (PDB ID: 7PSW, 1.21 Å resolution, *yellow*) and spin-labeled IPNS^S55C^–Fe–ACV–NO (PDB ID: 7POY, 1.75 Å resolution, *teal*) revealing α3 rearrangement because of NO binding. Note: In the spin-labeled anaerobic IPNS^S55C^–Fe–ACV structure, confident modeling of the spin label was not possible (see m*F*_o_–D*F*_c_ maps in Fig. S24*D*). *E*, distance comparison between spin labels on α3 and α10 observed *in crystallo* and in solution by DEER. *Left*, superimposition of spin-labeled IPNS^S55C^–Fe–ACV (PDB ID: 7PSW, *yellow*) with spin-labeled IPNS^S55C^–Fe–ACV–NO (PDB ID: 7POY, *teal*) views. In the NO complex, the spin label could be modelled, whereas in the anaerobic complex it was not modelled, though positive mF_o_-DF_c_ electron density adjacent to Cys55 is present (likely due to conformational mobility). An arc (radius = 3.79 nm) drawn around S323 indicates the distance of S323 to the spin label in the NO complex. All the positive m*F*_o_–D*F*_c_ electron density feature lies within the arc, implying a shorter distance between S323 and the spin label under anaerobic compared with NO-exposed conditions consistent with the EPR results as shown in solution. *Right*, distance distributions from DEER experiments with IPNS^S55C+S323C^–Fe–ACV ± NO reveal a longer distance (4.43 nm) in the NO complex compared with anaerobic conditions (4.33 nm). Note: The longer distance in the EPR data may be due to double spin labeling of C323 and C55 compared with single spin labeling of C55 *in crystallo*. Note: The distance distributions shown in Figure 5*E* (*right*) are a simplified replicate of Figure 4*C* for easier comparison. ACV, l-δ-(α-aminoadipoyl)-l-cysteinyl-d-valine; DEER, double electron–electron resonance; EPR, electron paramagnetic resonance; IPNS, isopenicillin N synthase; NO, nitric oxide; PDB, Protein Data Bank.

We solved a structure of the spin-labeled IPNS^S55C^–Fe–ACV complex after NO exposure (PDB ID: 7POY; 1.75 Å resolution). By contrast with the spin-labeled IPNS^S55C^–Fe–ACV complex, the structure obtained after NO exposure clearly revealed covalent modification of C55 with a 3-(acetomido)-PROXYL label *in crystallo* (Fig. 5, *A* and *C*). Analysis of the active site reveals octahedral coordination of the Fe *via* H214, D216, and H270, the ACV thiolate, and water, as well as substantial, but incomplete, NO binding (80%). Likely, as a result of the incomplete NO binding, two conformations of the substrate ACV were observed (Fig. 5*B*). One of these is the same as in the absence of NO in the anaerobic complex (ACV conf A 20%, *yellow*, Fig. S24, *B* and *D*). The rearranged conformation of ACV induced by NO binding (ACV conf B 80%, *teal*) is similar to that observed on incomplete O_2_ binding in the tr-SFX datasets of the IPNS–Fe–ACV–O_2_^⸣•−^ complex (PDB ID: 6ZAP; superoxo complex) (21). The active site and the overall structure of the IPNS–Fe–ACV–NO complex superimposes well with the previously reported IPNS–Fe–ACV–NO complexes (PDB ID: 6ZAN (21), C-α RMSD: 0.121 Å), with the notable exception of the covalent modification of C55. In the spin-labeled IPNS–Fe–ACV–NO complex, it appears that α3 is rearranged (>80%) compared with anaerobic IPNS–Fe–ACV. The new conformation is the same as that previously reported to be induced by O_2_/NO binding to the metal ion. Two conformations of the covalently reacted 3-(acetomido)-PROXYL spin label were modeled (Fig. 5*C*).

Comparison of the overall structures for the spin-labeled anaerobic IPNS^S55C^–Fe–ACV (PDB ID: 7PSW) and the NO-exposed IPNS^S55C^–Fe–ACV–NO (PDB ID: 7POY) complexes reveals differences in the conformations of β11 (residues 279–282) and α3 (residues 47–64) (Figs. 5*B* and S24, *A* and *B*) (21). NO binding apparently changes the conformation of the ACV valinyl carboxylate, which induces rearrangement of S281 on β11, in turn causing changes in the conformations of the adjacent residues Q280 and V282 with consequent conformational rearrangement of α3 (Fig. 5*B*). Besides the NO-induced rearrangements of β11 and α3 and the conformationally stable spin label in the NO complex, no other differences were observed in the IPNS^S55C^–Fe–ACV structures before and after NO exposure.

The combined crystallographic studies show that the presence of a spin label, at least on α3, does not, at least substantially, alter the conformational changes induced by NO/O_2_ binding compared with the structures missing a label, for example, for the IPNS–Fe–ACV–NO (PDB ID: 6ZAN) or IPNS–Fe–ACV–O_2_ (PDB ID: 6ZAP) complexes (21). They are thus consistent with the proposal that the observed changes in the ^19^F NMR and DEER analyses likely reflect similar conformational changes *in crystallo* and in solution.

## Discussion

**Figure 6:**
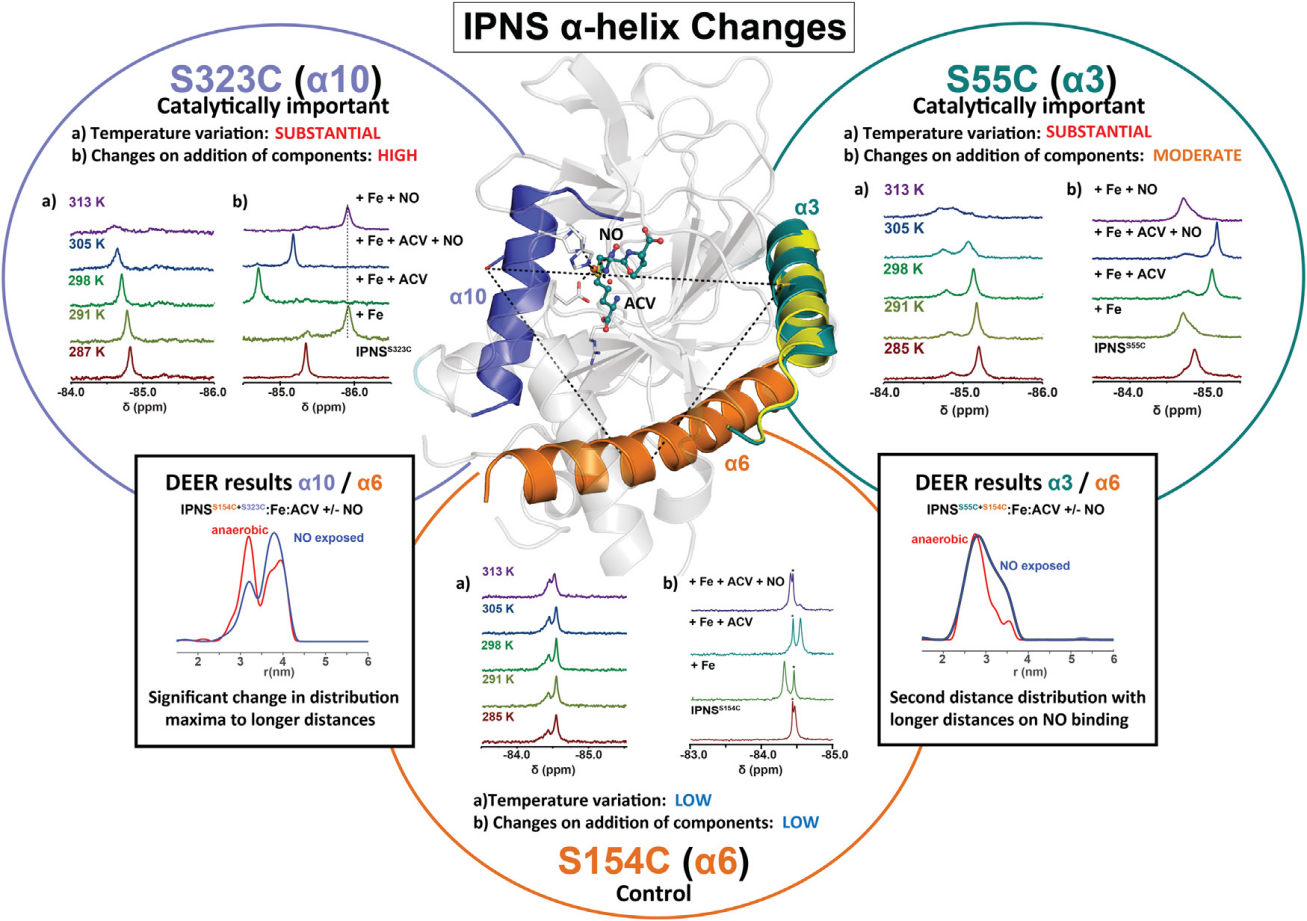
**Summary of biophysical studies on the IPNS α-helices.** Moderate–substantial functional and temperature-dependent changes are observed in the ^19^F NMR spectra on ACV–NO binding. By contrast, only minor changes are observed in the analogous spectra on ACV–NO binding for α6. The DEER experiments reveal a change of distance distributions between both spin centers to longer distances upon NO binding. The combined spectroscopic and crystallographic studies support a role of conformational changes of α3 and α10 in IPNS catalysis. Note: The information on chemical shifts and peak broadening of the ^19^F NMR figures as well as the distance distributions shown in this summary figure are simplified replicates of Figures 2, *A* and *C*, 3, *A*–*C* and 4, *A* and *B*. ACV, l-δ-(α-aminoadipoyl)-l-cysteinyl-d-valine; DEER, double electron–electron resonance; IPNS, isopenicillin N synthase; NO, nitric oxide.

The IPNS-catalyzed conversion of the linear tripeptide ACV substrate into the penicillin ring system involves both challenging C–H bond oxidations and precise control of the conformations of reactive intermediates. tr-SFX studies on the reaction of the IPNS–Fe–ACV complex with O_2_ using a crystal form that traps the product complex have revealed that conformational changes involving α3 and α10 are involved in penicillin ring formation, at least *in crystallo* (21). The combined studies in solution described here support roles for conformational changes involving α3 and α10 in IPNS catalysis. Although our ^19^F NMR studies relied on analysis of changes in chemical shift and peak shape, which might reflect differences in local chemical environment independent of the conformational changes observed by tr-SFX, across multiple analyses (Figs. 2, 3, 6 and S9–S17), with singly and doubly labeled α3 and α10 IPNS variants at varied temperatures, we consistently observed more substantial changes in the ^19^F NMR peaks for α3 and α10, compared with those for α6, which was not observed to undergo substantial conformational changes in the tr-SFX studies (21). Crystallographic studies on IPNS show that, at least in one case (α3), labeling of S55C does impact neither on the overall fold nor on the conformational changes induced by NO binding.

Thus, combined with the tr-SFX studies, the ^19^F NMR results presented here provide evidence that ordered sequential binding of Fe(II), ACV, and NO to IPNS can affect protein conformations in regions that are relatively remote from the active site, including α3 and α10. Analysis of the data obtained for IPNS^S55C^ (α3) (21) and IPNS^S323C^ (α10) titrations reveals moderate to substantial changes in the ^19^F chemical shifts on Fe(II), substrate, and NO binding. By contrast with these results, in the case of ^19^F-labeled IPNS^S154C^ (α6), only very minor changes in the ^19^F chemical shifts were observed. The results with singly labeled ^19^F IPNS variants were supported by those with the IPNS^S55C+S154C^ (α3/α6) and IPNS^S154C+S323C^ (α6/α10) double variants. Further evidence for conformational changes involving α3 and α10 was provided by temperature variation studies that revealed substantial temperature-dependent broadening/multiple signals of the ^19^F NMR signals in the Fe(II) and ACV complexes of IPNS^S55C^ and IPNS^S323C^; by contrast, only minor effects were observed for IPNS^S154C^ (Fig. 6). The temperature variation studies also showed that addition of Fe(II) and ACV increased the stabilities of the IPNS variant complexes in solution (Figs. S9, S10, S12, S13, S15, S16).

The combined results support the proposal of movements of α3/α10 during substrate and O_2_/NO binding, but imply that α6 undergoes only minor conformational changes, consistent with the tr-SFX results. The observation that there are no substantial changes in the ^19^F NMR spectra after exposure of the IPNS–Fe complex to NO in the absence of ACV, or in the IPNS–Zn–ACV and IPNS–Cd–ACV complexes after NO exposure, supports the proposal that conformational changes involving α3 and α10 are induced by active site metal ion binding of NO, and by implication of O_2_, in the presence of the substrate ACV. Note that the ^19^F NMR observations following NO binding likely reflect the early stages of O_2_-mediated reaction since NO reacts to form a stable complex. Consistent with the proposal that conformational movement of α6 is limited compared with α3 and α10 in multiple analyses, the ^19^F NMR peaks for IPNS^S154C^ were observed to be sharper than those for IPNS^S55C^ and IPNS^S323C^, as illustrated by the peak heights/width analysis in the IPNS^S55C+S154C^ and IPNS^S154C+S323C^ double variants upon substrate additions (Fig. 2, *E* and *F*).

By contrast with the relatively broad peaks observed in the IPNS–Fe(II)–ACV complexes, all the ^19^F NMR spectra for the IPNS–Zn–ACV and IPNS–Cd–ACV complexes manifested sharp signals (as especially pronounced in the temperature-dependent experiments [Figs. S10, S11, S13, S14, S16, and S17]; note that anaerobic IPNS–Fe(II)–ACV is diamagnetic and unlikely to affect peak broadening) (41). These observations suggest that of the tested metal ions, binding of the catalytically active Fe(II) might be more effective in inducing conformational changes, including in α3 and α10, that extend to the surface of IPNS. Unexpectedly, exposure of IPNS^S55C^–Fe–ACV, IPNS^S154C^–Fe–ACV, and IPNS^S323C^–Fe–ACV variant complexes to NO caused sharpening of their ^19^F NMR signals (Figs. 3, *D*–*F*, S10, S13 and S16). The sharpening in the IPNS–Fe–ACV–NO complexes is more pronounced with increased temperature; this trend clearly differs from that for the IPNS–Fe–ACV complexes in the absence of NO, where we observed broadening as a function of increased temperature. Although there are other possible explanations, we speculate that the peak sharpening observed on NO binding might reflect limitation of energetically accessible conformations, to control reactivity of the superoxide intermediate prior to stereoselective abstraction of the ACV cysteinyl C-3 hydrogen; the relatively broad nature of the peaks prior to NO addition may reflect conformational mobility to enable efficient transfer of O_2_ from bulk solution to the active site.

Spin labeling of the IPNS double Cys variants with 3-(2-iodoacetomido)-PROXYL enabled DEER experiments measuring the distance between the two spin centers. In all the analyzed samples, a shift of the distance distributions to longer maxima on NO binding to all the investigated IPNS–Fe–ACV complexes was observed. NO exposure of IPNS^S55C+S154C^–Fe–ACV revealed a major distance distribution of 2.8 nm similar to the anaerobic IPNS^S55C+S154C^–Fe–ACV complex and a species with a longer distance distribution of 3.4 nm; the latter is not present without NO and is likely because of movement of α3, although an altering of conformation populations in the NO-exposed samples cannot be excluded. The overall quality of the DEER analyses was compromised by relatively broad distance distributions, likely because of more than one conformation of the IPSL. Future work with a bifunctionally linked spin label may reduce conformational isomers giving narrower distance distributions (42, 43, 44). The IPSL linkage, however, with a rather large 10 to 15 Å full width at half maximum distance distribution, shows considerable sensitivity to steric variation, consistent with twisting helix motion, partial uncoiling, and linear translation (Fig. 5, *D* and *E*).

To support the proposed association of the ^19^F NMR and DEER studies with the tr-SFX analysis, we carried out crystallographic analyses on the spin-labeled IPNS^S55C^–Fe–ACV complex under anaerobic conditions and after exposure of single crystals to NO to give a spin-labeled IPNS^S55C^–Fe–ACV–NO complex. The results reveal conformational rearrangement of α3 upon NO binding giving a similar conformation as reported for the synchrotron-derived cryo IPNS–Fe–ACV–O_2_^⸣•−^ (PDB ID: 6ZAP) (21) and IPNS–Fe–ACV–NO complexes (PDB ID: 6ZAN) (21). In the anaerobic IPNS^S55C^–Fe–ACV complex, the spin label is directed away from the protein surface likely leading to conformational mobility as manifested by disordered electron density for it. After NO exposure, however, clear electron density for the spin label was apparent, and it was refined in two conformations. These observations probably, in part, reflect crystal lattice packing interactions but highlight the changes in α3 induced by NO/O_2_ binding. Consistent with the DEER distances for the IPNS^S55C+S323C^–Fe–ACV (4.33 nm) and IPNS^S55C+S323C^–Fe–ACV–NO (4.43 nm) complexes (Fig. 5*E*), analysis of distances between C55 and S323 in anaerobic (PDB ID: 7PSW) and NO-exposed rearranged complexes (PDB ID: 7POY) reveals a slightly shorter distance for the anaerobic complex.

The combined solution-based spectroscopic and crystallographic studies highlight the importance of conformational changes extending from the active site to the protein surface, including involving α3 and α10 (and likely other regions), during sequential substrate binding of Fe(II) and ACV to the IPNS active site. Such changes are also involved in binding of O_2_ as implied on the basis of observations with NO, which reacts with IPNS–Fe–ACV to give a stable complex (39, 41). Note, it is difficult to distinguish between conformational changes relating to O_2_ binding and those relating to early stages in reaction subsequent to O_2_ binding, something that we are investigating in ongoing tr-SFX and modeling studies. Evidence from the reported tr-SFX studies implies that the conformational mobilities of α3 and, to a lesser extent, α10 are reduced in the IPN product complex compared with the superoxide intermediate (and NO) complex (21), though are still increased relative to the ACV substrate complex, possibly reflecting motions to promote product release. Thus, conformational changes involving α3 and α10 and other regions play different roles at different intermediate stages in IPNS catalysis. Evidence for the different roles of correlated motions/conformational changes in regulation of intermediate reactivity in catalysis by 2OG oxygenases, which are structurally related to IPNS, has come from quantum mechanics/molecular mechanics modeling studies, including on the DNA demethylase AlkB and the JmjC histone demethylases (14, 45).

The roles of conformational changes involving substantial regions of an enzyme structure, particularly for reactions involving reactive intermediates, remain poorly understood and are not easy to predict with confidence by modeling (because of the large number of atoms involved, potentially including from solvent). Time-resolved crystallography can now enable monitoring of global changes in a protein fold on a short timescale (46, 47). When coupled with solution studies and computational modeling, time-resolved crystallography has considerable potential to define conformational changes during catalysis, as shown by the combined work on IPNS and mechanistically related oxygenases (*e.g.*, 3-hydroxyanthranilate-3,4-dioxygenase (48)) as well as other enzyme families such as kinases (38, 49). Aside from being of basic interest, the information provided by such time-resolved studies should be useful in engineering enzymes to alter their reactivity for biocatalytic purposes and may shed light on enzyme evolution including as to why substitutions away from the active site can help alter function as observed, for example, in (metallo) enzyme-mediated antibiotic resistance and biosynthesis, in the latter case including penicillin and cephalosporin biosynthesis, where 2OG oxygenases closely related in sequence to IPNS catalyze modification of the penicillin ring (1, 4, 50). Time-resolved studies on enzymes may also help to identify new types of small molecules that inhibit (or enhance) enzyme activity by altering the reactivity of intermediates compared with most small-molecule drugs/agrochemicals, which are active site binders, mostly identified on the basis of simple turnover/binding assays with rational design informed by static structural information. Most examples where more subtle (*e.g.*, allosteric) inhibition mechanisms have been identified, for example, in recent work on allosteric isocitrate dehydrogenase inhibitors (51, 52), have emerged from empirical screens rather than detailed knowledge of the roles of the overall protein fold in enzyme catalysis.

## Experimental procedures

### Materials and methods

Chemicals for preparation of buffers and crystallization screens were obtained from commercial suppliers and were used without further purification except where otherwise mentioned. ACV was synthesized by solid-phase peptide synthesis as reported (21). ACV purity was confirmed by NMR (Bruker AVIII HD 600 equipped with a BB-F/H N_2_; CryoProbe Prodigy).

### Site-directed mutagenesis of IPNS variants

A pCOLD I vector (Addgene) containing DNA encoding for IPNS (codon optimized for expression in *E. coli*) was used to produce IPNS variants with an N-terminal hexa-histidine tag and a 3C human rhinovirus (HRV3C) protease cleavage site (21) *via* site-directed mutagenesis. The five IPNS variants (IPNS^S154C^, IPNS^S323C^, IPNS^S55C+S154C^, IPNS^S55C+S323C^, and IPNS^S154C+S323C^) were produced using the Q5 site-directed mutagenesis kit (New England Biolabs) using the manufacturer’s protocol and primers as defined in Table S1. Following PCRs, DpnI digestions were conducted at 37 °C for 1 h. The plasmid-containing solutions were transformed into XL10 Gold ultracompetent cells (Agilent) using heat shock (42 °C for 30 s). Cells were incubated in 200 μl 2YT medium for 45 min at 37 °C. Cell colonies were grown overnight on 2YT agar plates containing ampicillin (50 μg ml^−1^). Single colonies were picked and used to inoculate 10 ml cultures in 2YT medium. Plasmid DNA was isolated using a GeneJET Plasmid Miniprep kit (Thermo Fisher Scientific), and the required DNA sequences were confirmed by sequencing (Eurofins).

### Production and purification of IPNS variants

IPNS variant production and purification was performed as reported (21). Plasmids encoding for the IPNS variants were transformed into *E. coli* BL21 cells using a heat-shock protocol and grown on 2YT agar plates containing ampicillin (50 μg ml^−1^). Single colonies were picked to inoculate a starter culture of 2YT medium (100 ml, 50 μg ml^−1^ ampicillin). Cells were grown overnight with shaking (150 rpm, 37 °C). The starter culture (6 ml, 1:100 v/v) was used to inoculate large-scale growth (12× 600 ml 2YT medium) containing ampicillin (50 μg ml^−1^). The cells were grown (150 rpm, 37 °C) until an absorbance of 0.6 at 600 nm was reached. IPTG (1 mM final concentration) was added, and the cultures were incubated overnight at 15 °C. Cells were harvested by centrifugation (11,000*g*, 10 min, 4 °C), and the cell pellet was stored at −80 °C.

Protein purification involved resuspension of the cell pellet with the IPNS variant in binding buffer (1:4 w:v; 50 mM Tris, 200 mM NaCl, 5 mM imidazole, pH 8.0) containing DNAse I (10 μg ml^−1^), PMSF (10 μg ml^−1^), and lysozyme (0.2 mg ml^−1^). Cell lysis was achieved by sonication (9 s on:9 s off, 60% amplitude, 12 min total time, 4 °C; Sonics Vibra-Cell); cell debris was removed by centrifugation (58,000*g*, 30 min, 4 °C). The supernatant was filtered (2 μm syringe filter) and loaded onto a nickel affinity column (5 ml HisTrap HP; GE Healthcare), pre-equilibrated with binding buffer (5 column volumes [CVs]). The loaded protein was washed with binding buffer (20 CVs). Elution was achieved by applying a gradient (20 CVs) from the binding buffer to the elution buffer (50 mM Tris, 200 mM NaCl, 500 mM imidazole, pH 8.0). SDS-PAGE analysis was used to identify protein-containing fractions, which were concentrated using a centrifugation tube (10 k molecular weight cutoff, 3000*g*, 4 °C; Merck Millipore). 3C protease (1:100 dilution, w:w) was added to the solution, and the mixture was incubated overnight at 5 °C. The protein solution was loaded onto a nickel affinity column (5 ml HisTrap HP; GE Healthcare), and untagged IPNS was eluted with 5 CVs of binding buffer. Successful cleavage of the hexa-histidine tag was confirmed by SDS-PAGE and protein MS analysis. Size-exclusion chromatography (Superdex 75 column, 300 ml; GE Healthcare) with size-exclusion buffer (50 mM Tris, 200 mM NaCl, pH 8.0) was carried out for further purification. Purified IPNS (>95% by SDS-PAGE) was loaded into a dialysis cassette (10 k molecular weight cutoff; Slide-A-Lyzer; Thermo Fisher Scientific) and buffer exchanged against dialysis buffer (50 mM Tris, 200 mM NaCl, 30 mM EDTA, 5 mM 1,10-phenantroline, pH 8.0, 1.5 l each, 4 × 3 h, 4 °C), followed by dialysis (6 × 3 h, 1.5 l each, 4 °C) against storage buffer (50 mM Tris, 200 mM NaCl, pH 8.0; Chelex resin treated; Bio-Rad) to remove residual EDTA. *Apo*-IPNS was concentrated to 50 mg ml^−1^, aliquoted, flash-frozen in liquid nitrogen, and stored at −80 °C.

### Labeling of IPNS variants with BTFA or 3-(2-iodoacetomido)-PROXYL

#### Labeling with 3-(2-iodoacetomido)-PROXYL

Three nitroxide spin labels, 3-(2-iodoacetomido)-PROXYL (Sigma–Aldrich), 3-maleimido-PROXYL (Sigma–Aldrich), and MTSL (Santa Cruz Biotechnology), were investigated for their performance in IPNS^S323C^ variant spin labeling. A 2 mM stock solution of the compounds was prepared in water; 150 μl of each of these solutions was added to 150 μl of a 200 μM IPNS^S323C^ solution (1:10 ratio of IPNS^S323C^:spin label). The reaction mixture was incubated at 5 °C, and samples were extracted at various time points (10 min, 1 h, 3 h, and 16 h). Reactions were quenched by removal of the spin label using a Bio-Rad Micro Bio-spin column (pre-equilibrated with storage buffer, see previous one). Protein MS analysis was carried out to determine labeling efficiency. Under the tested conditions, only 3-(2-iodoacetomido)-PROXYL labeling resulted in monolabeled IPNS^S323C^, even after an extended reaction period (16 h). With the other spin labels, multiple reactions/labeling were observed.

Spin labeling of the other IPNS variants was carried out with 3-(2-iodoacetomido)-PROXYL as described previously. Monolabeling (predicted mass increase of 198 Da) was observed for IPNS^S55C^, IPNS^S154C^, and IPNS^S323C^ variants, and double labeling was observed for IPNS^S55C+S154C^ and IPNS^S154C+S323C^ variants after an extended reaction time of 24 h.

#### Labeling with BTFA

A solution of an IPNS variant (350 μM) was incubated with tris(2-carboxyethyl)phosphine (TCEP; 350 μM, 1 equivalent) in storage buffer for 5 min at 4 °C. BTFA (10.5 mM final concentration, 30 equivalents) was added, and the reaction mixture was incubated overnight at 4 °C. Protein MS analysis was used to confirm successful labeling of IPNS. The observed mass increase of IPNS upon monolabeling is 128 Da. The predominant mass difference observed was 18 Da higher than the predicted mass increase (based on the calculated mass of CH_2_C(O)F_3_, 110 Da), suggesting that the ketone exists predominantly in its hydrated form, as previously reported (21).

The reaction of the IPNS variants with BTFA was quenched by buffer exchange using a PD-10 column that had been pre-equilibrated with storage buffer (50 mM Tris, 200 mM NaCl, pH 8.0, Chelex resin treated). Protein MS analysis indicated a labeling efficiency of >95%.

### NMR measurements

^19^F NMR experiments on the IPNS variants were recorded using a Bruker AVIII HD 600 NMR instrument equipped with a BB-F/H Prodigy N_2_ CryoProbe. The samples were prepared in 5 mm regular or J Young valve NMR tubes (5 mm; Norell) at 298 K. Temperature series were carried out at variable temperatures of 285, 291, 298, 305, and 313 K. ^19^F NMR spectra were referenced to CF_3_CO_2_H (100 μM, at δ_F_ = −76.55 ppm) in all experiments, regardless of pH shifts because of temperature effects on Tris buffer. ^19^F NMR spectra were processed with 3 Hz Lorentzian line broadening using MestReNova 14.1 (MestReLabs; www.mestrelab.com) and TopSpin 3.6.1 (Bruker; www.bruker.com).

### Sample preparation for ^19^F NMR measurements

Sample preparation was performed under anaerobic conditions (<2 ppm O_2_) in an anaerobic chamber (Belle Technology). Stock solutions of NMR buffer (25 mM Tris-d_11_ buffer, pH 7.5 in water), TFA (10 mM in water), and D_2_O were deoxygenated by argon purging (30 min) before placing in an anaerobic chamber. Solids (FeSO_4_, ZnCl_2_, CdCl_2_, and ACV) and NMR tubes were transferred into the anaerobic chamber and equilibrated before use. Stock solutions of the *apo*-IPNS variants (20–30 mg ml^−1^) were transferred into the anaerobic chamber prior to use. Final solutions of ACV (100 mM in NMR buffer), FeSO_4_ (50 mM in water), ZnCl_2_ (50 mM in water), and CdCl_2_ (50 mM in water) were prepared in the anaerobic chamber. The total volume of a typical sample was 450 μl and contained 120 μM ^19^F-labeled IPNS variant, 100 μM TFA (4.5 μl, internal standard), ± 600 μM metal ion (5.4 μl, 5 equivalents), ± 7.68 mM ACV (34.5 μl, 64 equivalents), and 10% (v/v) D_2_O (45 μl). After equilibration, samples were transferred from Eppendorf tubes (1.5 ml) into 5 mm J Young valve NMR tubes for analysis.

For subsequent additions, an NMR tube containing the sample was transferred back into the glovebox and equilibrated for 5 to 10 min before opening. The appropriate solution was added, and the tube closed and inverted several times to equilibrate. NO exposure of a sample inside a J Young valve NMR involved custom glass apparatus as described (21).

### EPR measurements

Collection of DEER data in the Centre for Advanced ESR in the Department of Chemistry, University of Oxford, involved the use of a Bruker E580 EleXSys II instrument with a SpinJet AWG at Q-band, ca. 33.8 GHz with a Bruker EN5107D2-W1 resonator. The temperature was held at 50 K with helium flow through an Oxford Instruments CF935O cryostat whilst control over temperature stability was performed by an Oxford Instruments Mercury unit. The standard four-pulse DEER measurement (53) involved four “default” Gaussian pulses of 22 (±2) ns length, with the ELDOR pulse at +100 MHz from observe pulses, and the ELDOR pulses were positioned at the spectral maximum of the nitroxide field sweep signal intensity. A starting tau value of 260 ns was used for the primary echo, with eight increments of 16 ns to average deuterium modulations, and 4000 ns was used for the refocused echo.

### Sample preparation for EPR measurements

EPR samples were prepared under anaerobic conditions (<2 ppm O_2_) in an anaerobic chamber (Belle Technology) with all stock solutions, and solids were deoxygenated as described previously. A 40 mM ACV stock solution in EPR buffer (25 mM Tris, 200 mM NaCl, in D_2_O, pH 8.0) and a 100 mM metal ion solution (either FeSO_4_^.^7H_2_O in water) were prepared in the anaerobic chamber. The metal solution was diluted to 1 mM in EPR buffer. The EPR sample (final volume: 25 μl) was achieved by adjusting the concentration of the IPNS variant to 150 μM with 3 μl of ACV (stock: 40 mM in EPR buffer, final: 4.8 mM, 32 equivalents), 3.64 μl of the metal ion (stock: 1 mM, final: 145.5 μM, 0.97 equivalents), and 30% (v/v) glcycerol-d^8^ (Santa Cruz Biotechnology; 7.5 μl). NO exposure of the samples was carried out as described previously (21). The solution was homogenized and transferred into an 1.2 inner diameter × 1.6 mm outer diameter clear-fused quartz tube (“Ilmasil” quartz from Qsil GmbH), cryocooled, and stored in liquid nitrogen.

### Protein MS

Protein LC–MS analyses were conducted using a Xevo G2-XS mass spectrometer to investigate the mass of the IPNS variants before and after incubation with 3C protease, as well as after ^19^F or spin labeling to confirm labeling efficiency (>95%). The protein solutions were injected to a ProSwift RP-4H column (1 × 50 mm). The IPNS variants were eluted with a gradient of acetonitrile in water (with water and acetonitrile supplemented with formic acid, 0.1%): 0 to 10 min (5–95%), 10 to 12 min (95%), 12 to 13 min (95–5%), and 13 to 15 min (5%). The obtained mass spectra were deconvoluted using MassLynx (Waters Cooperation), version 4.1 masses are given in Table S2.

### Activity assays and SPE MS

Activity assays for the labeled and unlabeled IPNS variants were performed using cofactor stock solutions with conditions: l-ascorbic acid: 100 mM in MQ-grade water; ammonium iron(II) sulfate hexahydrate (NH_4_)_2_Fe(SO_4_)_2_·6H_2_O: 100 mM in 20 mM HCl diluted to 10 mM in MQ-grade water, TCEP: 100 mM in MQ-grade water, potassium clavulanate: 50 mM in MQ-grade water, which were freshly prepared from commercially sourced solids (Sigma–Aldrich). The tripeptide ACV was prepared and purified as reported (21).

The assay was performed in 2 ml deep well blocks (Greiner Bio-One GmbH) with final concentrations of enzyme (2 μM), ACV (400 μM, from an initial stock solution: 100 mM, in 25 mM Tris, 100 mM NaCl [pH 8.0]), (NH_4_)_2_Fe(SO_4_)_2_ (100 μM), TCEP (800 μM), l-ascorbate (800 μM), and potassium clavulanate (200 μM) under ambient O_2_ levels. The reaction was initiated by addition of enzyme, and the reaction was monitored in real time using SPE coupled to MS.

MS analyses were performed using a RapidFire RF 365 high-throughput sampling robot (Agilent) attached to an iFunnel Agilent 6550 accurate-mass quadrupole time-of-flight mass spectrometer operated in the positive ionization mode. Assay samples were aspirated under vacuum for 0.6 s and loaded onto a C4 SPE cartridge. After loading, the C4 SPE cartridge was washed with 0.1% (v/v) aqueous formic acid to remove nonvolatile buffer salts (5.5 s, 1.5 ml min^−1^). The peptide was eluted from the SPE cartridge with 0.1% (v/v) aqueous formic acid in 85/15 (v/v) acetonitrile/water into the mass spectrometer (5.5 s, 1.25 ml min^−1^), and the SPE cartridge re-equilibrated with 0.1% (v/v) aqueous formic acid (0.5 s, 1.25 ml min^−1^). The mass spectrometer was operated using the MassHunter Workstation B.08.00 software (Agilent), the mass spectrometer parameters were capillary voltage (4000 V), nozzle voltage (1000 V), fragmentor voltage (365 V), gas temperature (280 °C), gas flow (13 l min^−1^), sheath gas temperature (350 °C), sheath gas flow (12 l min^−1^). The *m/z* +1 charge state of the ACV substrate was used to extract ion chromatogram data, peak areas were integrated using RapidFire Integrator 4.3.0 (Agilent). Data were exported into Microsoft Excel and used to calculate the percent conversion of the ACV substrate.

### Crystal growth of spin-labeled IPNS and data collection

The 3-(2-iodoacetomido)-PROXYL spin-labeled *apo*-IPNS^S55C^ variant was used for crystallization. IPNS crystals were grown in 24-well hanging drop VDX plates (Hampton Research), anaerobically in an anaerobic chamber (Belle Technologies; <2 ppm O_2_). The IPNS crystallization solution was prepared by mixing freshly prepared FeSO_4_ (4 μl, 100 mM) with IPNS (in 25 mM Tris [pH 8.0], 80 μl of 52 mg ml^−1^), followed by addition of ACV (2.0 mg in 20 μl of 25 mM Tris [pH 8.5]). A screen, varying the pH (0.1 M Tris [pH 8.1–8.7] in steps of pH 0.2, *vertical axis*) and the salt concentration (Li_2_SO_4_ 1.5–2.0 M in steps of 0.1, *horizontal axis*), was carried out (54). Crystals were grown using the hanging drop method by combining the reservoir solutions (3 μl) and protein solutions (3 μl). Crystals (120 μm) formed after 24 to 72 h, and some were exposed to NO as reported (21). After exposure of the crystals to NO (3 h, 1.000 ppm in nitrogen), the crystals were supplemented with cryoprotectant (mother liquor with 20% [v/v] glycerol mixed with crystal containing drop 1:1), then harvested with a nylon loop and cryocooled by rapid plunging into liquid nitrogen before data collection. Data for single crystals were collected at 100 K using synchrotron radiation at the Diamond Light Source beamline I03, then indexed, integrated, and scaled using the Xia2 pipelines (Table S3) (55). The crystal structures were determined by molecular replacement using the PHASER subroutine in PHENIX (56) using the ^19^F-labeled IPNS^S55C^–Fe–ACV structure (PDB ID: 6ZAM) (21) as search model. The structural model was optimized by iterative cycles of manual rebuilding in Coot (57) and crystallographic refinement using phenix.refine (56).

## Data and materials availability

All data needed to evaluate the conclusions are present in the article and/or the Supporting information. The atomic coordinates and structure factors are deposited in the PDB accession codes: 7PSW (IPNS^S55C^–Fe–ACV spin labeled) and 7POY (IPNS^S55C^–Fe–ACV–NO spin labeled, rearranged).

## Supporting information

This article contains supporting information (21, 58, 59).

## Conflict of interest

The authors declare that they have no conflicts of interest with the contents of this article.
