# Supplementary material for: Spectroscopic studies reveal details of substrate-induced conformational changes distant from the active site in isopenicillin N synthase
Source: J Biol Chem. 2022 Jul 11;298(9):102249. doi: 10.1016/j.jbc.2022.102249 (PMC9403350; doi:10.1016/j.jbc.2022.102249)
Supplement: Supplemental Figures S1–S24 and Tables S1–S3 [file mmc1.pdf]

Spectroscopic studies reveal details of substrate-induced conformational changes distant from the active site in isopenicillin N synthase

Patrick Rabe,[1,*] Carla C. Walla,[1] Noelle K. Goodyear,[1] Jordan Welsh,[1,2] Rebecca Southwart,[1] Ian Clifton,[1] James D. S. Linyard,[1] Anthony Tumber,[1] Tim D. W. Claridge,[1] William K. Myers,[2,*] and Christopher J. Schofield[1,*]

[1]Chemistry Research Laboratory, Department of Chemistry and the Ineos Oxford Institute for Antimicrobial Research, University of Oxford, 12 Mansfield Road, Oxford OX1 3TA, United Kingdom.

[2] Inorganic Chemistry Laboratory, Department of Chemistry, University of Oxford, South Parks Road, Oxford OX1 3QR, UK.

[*]Corresponding authors: Email: christopher.schofield@chem.ox.ac.uk; william.myers@chem.ox.ac.uk; patrick.rabe@chem.ox.ac.uk.

Supporting Information

Table S1

Primers used for site-directed mutagenesis.

**IPNS variant**	**Starting template**	**Primer Name**	**Sequence**
IPNS^{S55C},(1)	pCOLD_IPNS(1)	IPNS_S55C_fwd	cattaatgtgcagcgtctgtgccag aaaaccaaagaatt
		IPNS_S55C_rev	aattctttggttttctggcacagacgc tgcacattaatg
IPNS^{S154C}	pCOLD_IPNS(1)	IPNS_S154C_fwd	ctgagctgcgcactgctgaaag
		IPNS_S154C_rev	gtttttggtctgagctgcgcactg
IPNS^{S323C}	pCOLD_IPNS(1)	IPNS_S323C_fwd	ctggtttgcctgattaacaaaaatgg ccag
		IPNS_S323C_rev	ctgcagaatggtctggtttgcctgat taac
IPNS$^{S55C+S154C}$	pCOLD_IPNS^{S55C}(1)	IPNS_S154C_fwd	ctgagctgcgcactgctgaaag
		IPNS_S154C_rev	gtttttggtctgagctgcgcactg
IPNS$^{S55C+S323C}$	pCOLD_IPNS^{S55C}(1)	IPNS_S323C_fwd	ctggtttgcctgattaacaaaaatgg ccag
		IPNS_S323C_rev	ctgcagaatggtctggtttgcctgat taac
IPNS$^{S154C+S323C}$	pCOLD_IPNS^{S154C}	IPNS_S323C_fwd	ctggtttgcctgattaacaaaaatgg ccag
		IPNS_S323C_rev	ctgcagaatggtctggtttgcctgat taac

Table S2

Masses of IPNS variants in Da with predicted masses in paratheses.

**IPNS variant**	**His-tagged**	**untagged**	**^{19}F-labelled***	**spin-labelled**
IPNS^{S55C}	41592 (41741)	37774 (37774)	37902 (37906)	37972 (37972)
IPNS^{S154C}	41609 (41741)	37775 (37774)	37904 (37906)	37972 (37972)
IPNS^{S323C}	41592 (41741)	37774 (37774)	37903 (37906)	37972 (37972)
IPNS$^{S55C+S154C}$	41724 (41757)	37791 (37790)	n/a	38186 (38186)
IPNS$^{S55C+S323C}$	n/a	37790 (37790)	38047 (38046)	n/a
IPNS$^{S154C+S323C}$	n/a	37791 (37790)	38047 (38046)	38186 (38186)

* Note that the predicted mass difference based on the calculated mass of $CH_2C(O)CF_3$ (110 Da) was higher by 18 Da than observed, suggesting that the ketone exists mainly in its hydrated form, as reported.(1)

Table S3

**Data collection and refinement statistics.**		
**Data Collection and Refinement Statistics**	**IPNS^{S55C}:Fe:ACV spin labelled** (PDB ID: 7PSW)	**IPNS^{S55C}:Fe:ACV:NO spin labelled, rearranged** (PDB ID: 7POY)
**Data Collection (T in K)**	MX (100)	MX (100)
Beamline (Wavelength, Å)	DLS I03 (0.9763)	DLS I03 (0.9763)
Detector	Eiger2 XE 16M	Eiger2 XE 16M
Data Processing	Xia2	Xia2
Space group	$P\,2_1\,2_1\,2_1$	$P\,2_1\,2_1\,2_1$
Cell dimensions		
a,b,c (Å)	46.7, 71.4, 101.1	41.4, 74.9, 101.0
α, β, γ (°)	90, 90, 90	90, 90, 90
No. of molecules/ASU	1	1
No. reflections	103419 (10086)*	32521 (1576)*
Resolution (Å)	58.35-1.21 (1.23-1.21)*	41.89-1.75 (1.78-1.75)*
$R_{meas}(I)$	0.052 (0.478)*	0.240 (2.614)*
I/σI	24.2 (1.60)*	8.30 (0.60)*
CC-half	1.000 (0.922)*,#	0.997 (0.585)*
Completeness (%)	100 (97.2)*	100 (100)*
Multiplicity	12.20 (7.20)*	13.10 (13.30)*
Wilson B value (Å^2)	11.96	22.26
**Refinement**	PHENIX	PHENIX
R_{work}/R_{free+}	0.1433/0.1627	0.1806/0.2168
No. atoms	3359	3061
- Enzyme	2834	2663
- Ligand	76	86
- Water	479	312
Average B-factors	18.40	25.39
- Enzyme (Å^2)	15.93	24.21
- Ligand (Å^2)	30.68	36.84
- Water (Å^2)	31.87	32.39
R.m.s deviations		
- Bond lengths (Å)	0.010	0.005
- Bond angles (°)	1.07	0.729

*Highest resolution shell in parentheses. # Resolution cut-off of 1.21 Å was selected, since the detector limit used during data collection was insufficient for the quality of data and reflections in higher resolution shell suffered from low completeness. DLS = Diamond Light Source.

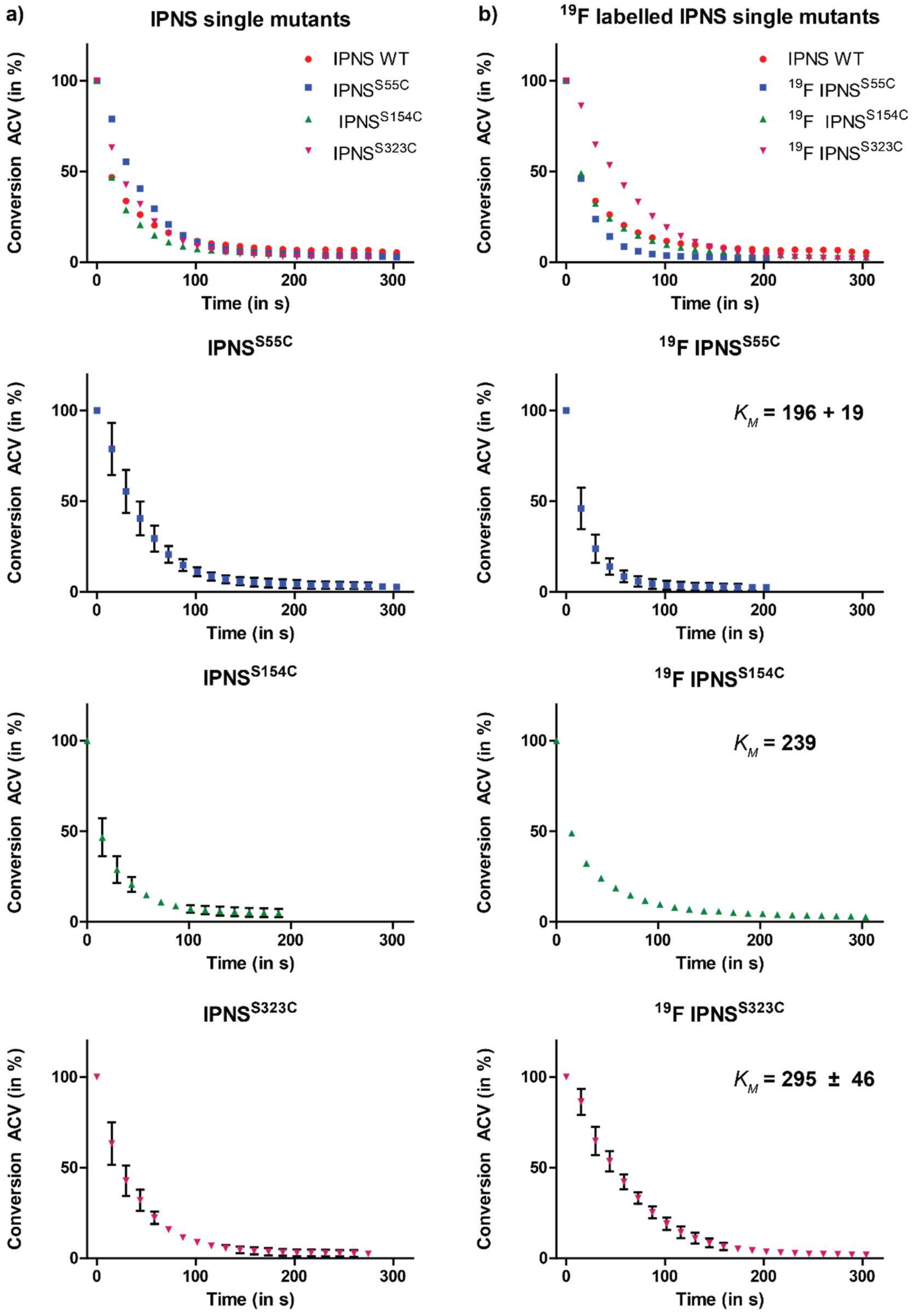

Figure 1. Activity of IPNS variants by solid phase extraction (SPE) mass spectrometry. Turnover was monitored in real time using the following conditions: IPNS variant (2 μM), ACV (300 μM), $(NH_4)_2Fe(SO_4)_2$ (100 μM), TCEP (800 μM), L-Ascorbate (800 μM) and potassium clavulanate (200 μM) under ambient O_2, in 25mM Tris, 100 mM NaCl pH 8.0. Note: Turnover of ^{19}F labelled IPNS^{S154C} was measured once; however, the ^{19}F labelled IPNS$^{S55C+S154C}$ and IPNS$^{S154C+S323C}$ double variants containing the S154C mutation are similar to WT IPNS. The K_M values of singly ^{19}F labelled IPNS variants are similar to the WT K_M of 206 μM ar pH 7.94)(2), with a somewhat higher K_M for ^{19}F IPNS^{S323C}.

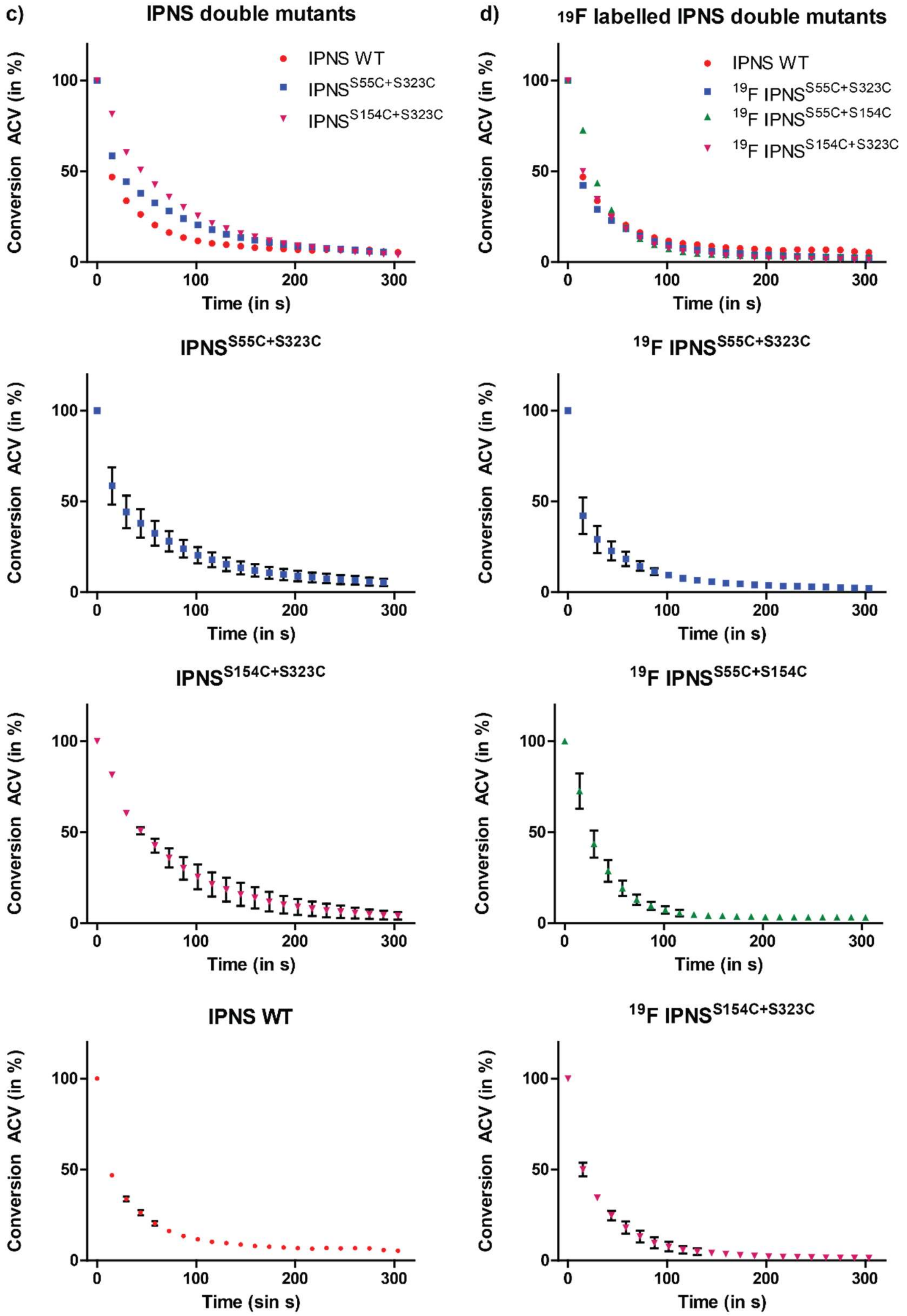

Figure 1. Activity of IPNS variants by solid phase extraction (SPE) mass spectrometry. Turn over was monitored in real time with the following conditions: IPNS variant (2 μM), ACV (300 μM), $(NH_4)_2Fe(SO_4)_2$ (100 μM), TCEP (800 μM), L-Ascorbate (800 μM) and potassium clavulanate (200 μM) under ambient O_2 levels in 25mM Tris, 100 mM NaCl pH 8.0.

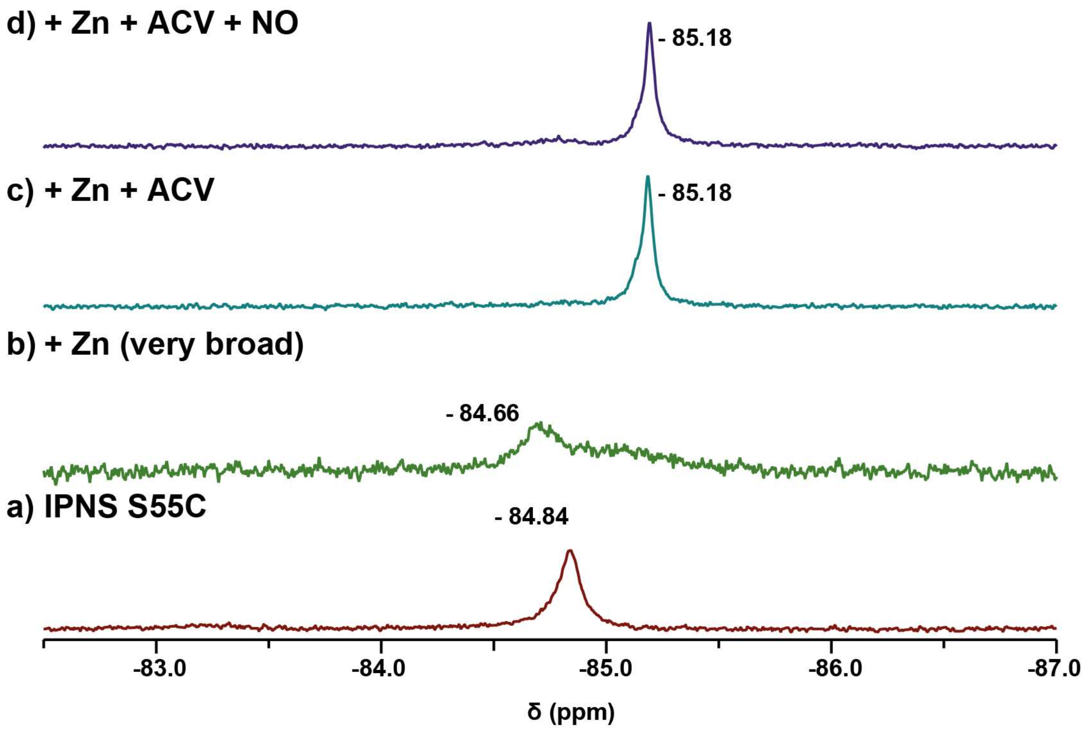

Figure 2. **^{19}F NMR spectra for reactions of ^{19}F labelled $IPNS^{S55C}$ with Zn(II) and ACV, and exposure to nitric oxide (NO).** a) $IPNS^{S55C}$ (120 μM) and CF_3CO_2H (100 μM) in Tris-d_{11} (25 mM, in H_2O, pH 7.5) and D_2O (50 μL, 10% (v/v)) (this mixture is referred to as ^$IPNS^{S55C}$); (b) ^$IPNS^{S55C}$ with an excess of Zn(II) (5 eq., 600 μM); (k) ^$IPNS^{S55C}$:Zn(II) with an excess of ACV (64 eq., 7.68 mM); (i) ^$IPNS^{S55C}$:Zn(II):ACV exposed to NO (1000 ppm in N_2) for 45 min. Note analogous experiments with Fe and Cd have been reported previously.(1)

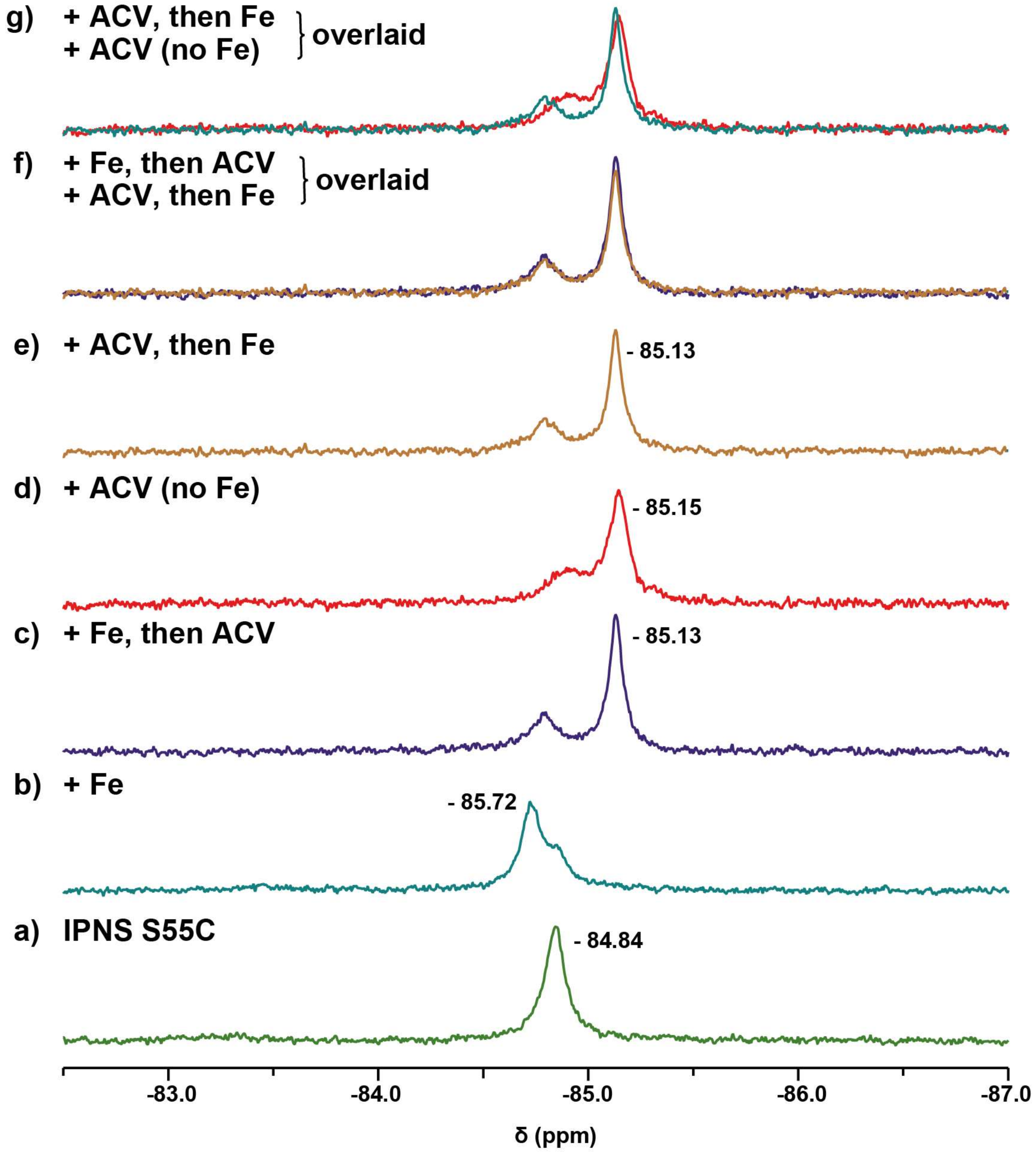

Figure 3. ^{19}F NMR spectra for reactions of ^{19}F labelled IPNS^{S55C} with Fe(II) and ACV. a) IPNS^{S55C} (120 μM) and CF_3CO_2H (100 μM) in Tris-d_{11} (25 mM, in H_2O, pH 7.5) and D_2O (50 μL, 10% (v/v)) (this mixture is referred to as ^IPNS^{S55C}); (b) ^IPNS^{S55C} with an excess of Fe(II) (5 eq., 600 μM); (c) ^IPNS^{S55C}:Fe(II) with an excess of ACV (64 eq., 7.68 mM); (d) ^IPNS^{S55C} with an excess of ACV (64 eq., 7.68 mM), (e) ^IPNS^{S55C}:ACV with an excess of Fe(II) (5 eq., 600 μM); (f) Overlay of IPNS:Fe:ACV spectra *(c)* and *(e)*. Spectra differ in order of Fe(II) and ACV additions; (g) Overlay of IPNS:Fe:ACV and IPNS:ACV spectra *(d)* and *(e)*, respectively. This spectral overlay indicates small differences in the ^{19}F NMR shifts in both the main peak and broad side peak, suggesting the possible presence of a dynamic second conformation.

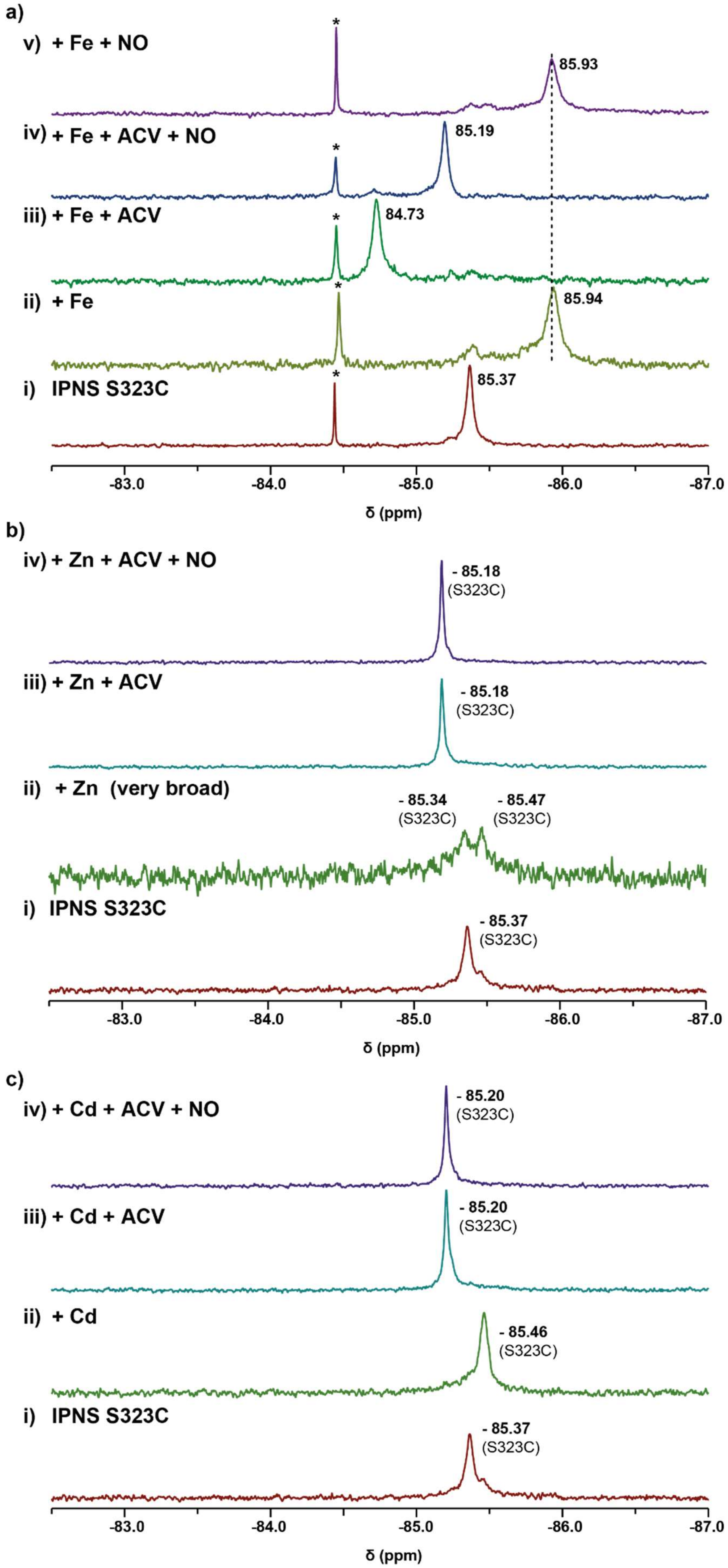

a)
v) + Fe + NO
85.93
iv) + Fe + ACV + NO
85.19
iii) + Fe + ACV
84.73
ii) + Fe
85.94
i) IPNS S323C
85.37
-83.0
-84.0
-85.0
-86.0
-87.0
δ (ppm)
b)
iv) + Zn + ACV + NO
- 85.18 (S323C)
iii) + Zn + ACV
- 85.18 (S323C)
ii) + Zn (very broad)
- 85.34 (S323C)
- 85.47 (S323C)
i) IPNS S323C
- 85.37 (S323C)
-83.0
-84.0
-85.0
-86.0
-87.0
δ (ppm)
c)
iv) + Cd + ACV + NO
- 85.20 (S323C)
iii) + Cd + ACV
- 85.20 (S323C)
ii) + Cd
- 85.46 (S323C)
i) IPNS S323C
- 85.37 (S323C)
-83.0
-84.0
-85.0
-86.0
-87.0
δ (ppm)

Figure 4. ^{19}F NMR spectra for titrations of ^{19}F labelled IPNS^{S323C} with Fe(II), Cd(II), Zn(II) and ACV, and exposure to nitric oxide (NO). a) i) IPNS^{S323C} (120 μM) and CF_3CO_2H (100 μM) in Tris-d_{11} (25 mM, in H_2O, pH 7.5) and D_2O (50 μL, 10% (v/v)) (this mixture is referred to as ^IPNS^{S323C}); (ii) ^IPNS^{S323C} with an excess of Fe(II) (5 eq., 600 μM); (iii) ^IPNS^{S323C}:Fe(II) with an excess of ACV (64 eq., 7.68 mM); (iv) ^IPNS^{S323C}:Fe(II):ACV exposed to NO (1000 ppm in N_2) for 45 min; (v) ^IPNS^{S323C}:Fe(II) (71 μM) exposed to NO (1000 ppm in N_2) for 45 min. Note: The * labelled ^{19}F NMR signal is due to residual 3-bromo-1,1,1-trifluoropropane-2,2-diol from the labelling process. b) i) ^IPNS^{S323C}; (ii) ^IPNS^{S323C} with an excess of Zn(II) (5 eq., 600 μM) (Note, the broad nature of the peak after addition of Zn(II)); (iii) ^IPNS^{S323C}:Zn(II) with an excess of ACV (64 eq., 7.68 mM); (iv) ^IPNS^{S323C}:Zn(II):ACV exposed to NO (1000 ppm in N_2) for 60 min. c) i) ^IPNS^{S323C}; (ii) ^IPNS^{S323C} with an excess of Cd(II) (4 eq., 480 μM); (iii) ^IPNS^{S323C}:Cd(II) with an excess of ACV (64 eq., 7.68 mM); (iv) ^IPNS^{S323C}:Cd(II):ACV exposed to NO (1000 ppm in N_2) for 35 min.

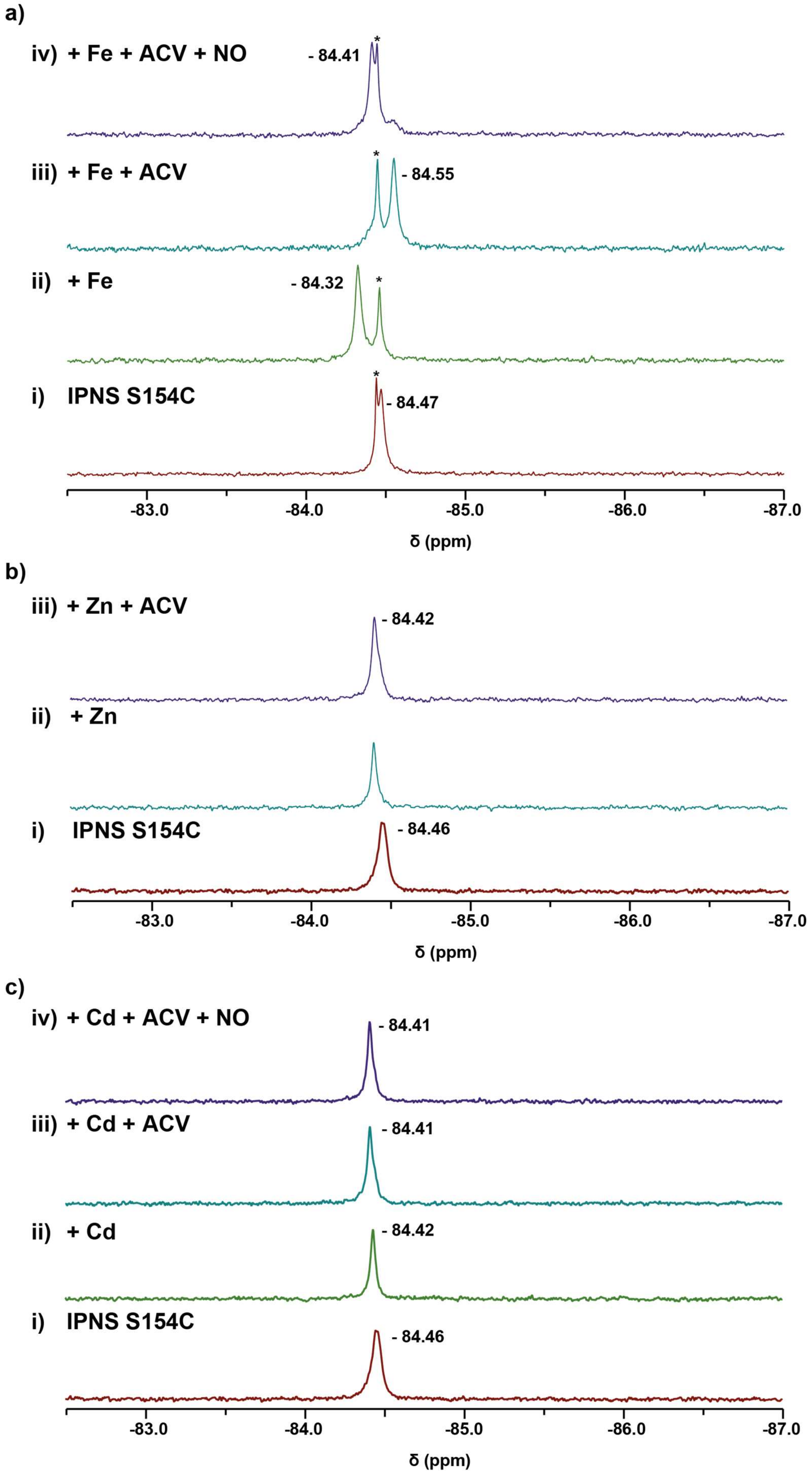

a)
iv) + Fe + ACV + NO
- 84.41
*
iii) + Fe + ACV
*
- 84.55
ii) + Fe
- 84.32
*
i) IPNS S154C
*
- 84.47
-83.0
-84.0
-85.0
-86.0
-87.0
δ (ppm)
b)
iii) + Zn + ACV
- 84.42
ii) + Zn
i) IPNS S154C
- 84.46
-83.0
-84.0
-85.0
-86.0
-87.0
δ (ppm)
c)
iv) + Cd + ACV + NO
- 84.41
iii) + Cd + ACV
- 84.41
ii) + Cd
- 84.42
i) IPNS S154C
- 84.46
-83.0
-84.0
-85.0
-86.0
-87.0
δ (ppm)

Figure 5. ^{19}F NMR spectra of reactions of ^{19}F labelled IPNS^{S154C} with Fe(II), Cd(II), Zn(II) and ACV, and exposure to nitric oxide (NO). a) i) IPNS^{S154C} (150 μM) and CF_3CO_2H (100 μM) in Tris-d_{11} (25 mM, in H_2O, pH 7.5) and D_2O (50 μL, 10% (v/v)) (this mixture is referred to as ^IPNS^{S154C}); (ii) ^IPNS^{S154C} with an excess of Fe(II) (5 eq., 750 μM); (iii) ^IPNS^{S154C}:Fe(II) with an excess of ACV (64 eq., 9.6 mM); (iv) ^IPNS^{S154C}:Fe(II):ACV exposed to NO (1000 ppm in N_2) for 30 min. Note, the *labelled ^{19}F NMR signal is due to residual 3-bromo-1,1,1-trifluoropropane-2,2-diol from the labelling process. b) i) ^IPNS^{S154C}; (ii) ^IPNS^{S154C} with an excess of Zn(II) (5 eq., 600 μM) and (iii) ^IPNS^{S154C}:Zn(II) with an excess of ACV (64 eq., 7.68 mM). c) i) ^IPNS^{S154C}; (ii) ^IPNS^{S154C} with an excess of Cd(II) (5 eq., 600 μM); (iii) ^IPNS^{S154C}:Cd(II) with an excess of ACV (64 eq., 7.68 mM); (iv) ^IPNS^{S154C}:Cd(II):ACV exposed to NO (1000 ppm in N_2) for 45 min.

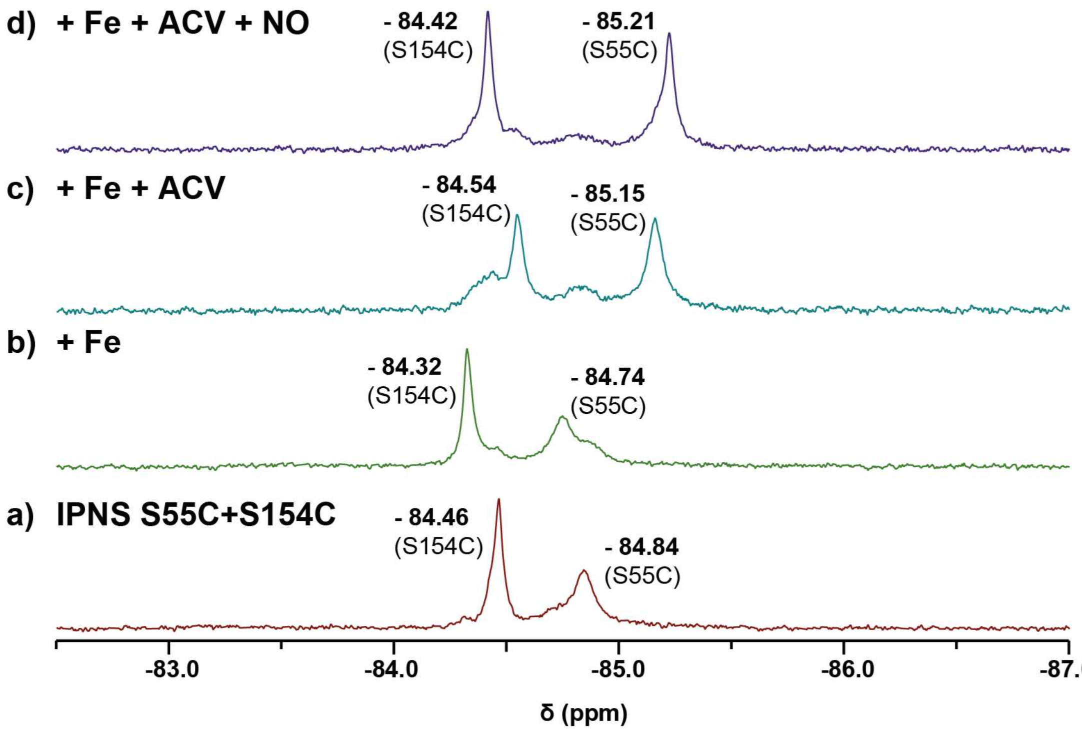

Figure 6. ^{19}F NMR spectra of reactions of double ^{19}F labelled IPNS$^{S55C/S154C}$ with Fe(II) and ACV, and exposure to nitric oxide (NO). a) IPNS$^{S55C/S154C}$ (120 μM) and CF_3CO_2H (100 μM) in Tris-d_{11} (25 mM, in H_2O, pH 7.5) and D_2O (50 μL, 10% (v/v)) (this mixture is referred to as ^IPNS$^{S55C/S154C}$); (b) ^IPNS$^{S55C/S154C}$ with an excess of Fe(II) (10 eq., 1.2 mM); (c) ^IPNS$^{S55C/S154C}$:Fe(II) with an excess of ACV (64 eq., 7.68 mM); (d) IPNS$^{S55C/S154C}$:Fe(II):ACV exposed to NO (1000 ppm in N_2) for 80 min.

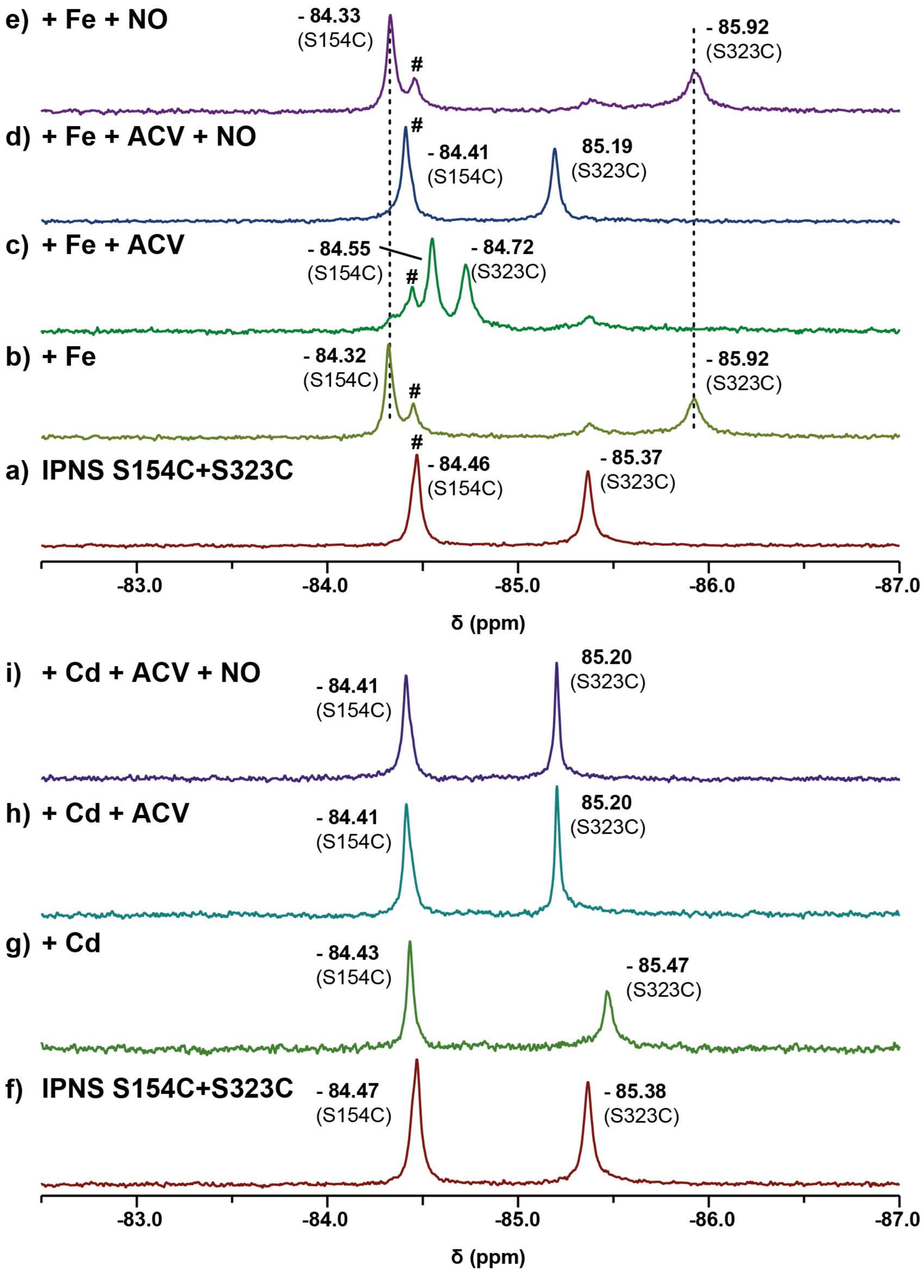

Figure 7. ^{19}F NMR spectra of reactions of double ^{19}F labelled $IPNS^{S154C/S323C}$ with Fe(II), Cd(II) and ACV, and exposure to nitric oxide (NO). a) $IPNS^{S154C/S323C}$ (120 μM) and CF_3CO_2H (100 μM) in Tris-d_{11} (25 mM, in H_2O, pH 7.5) and D_2O (50 μL, 10% (v/v)) (this mixture is referred to as ^$IPNS^{S154C/S323C}$); (b) ^$IPNS^{S154C/S323C}$ with an excess of Fe(II) (5 eq., 600 μM); (c) ^$IPNS^{S154C/S323C}$:Fe(II) with an excess of ACV (64 eq., 7.68 mM); (d) ^$IPNS^{S154C/S323C}$:Fe(II):ACV exposed to NO (1000 ppm in N_2) for 30 min: (e) ^$IPNS^{S154C/S323C}$:Fe(II) exposed to NO (1000 ppm in N_2) for 45 min.. Note: The ^{19}F signal indicated by # is presumably a second conformation as confirmed by diffusion analysis as described in

Figure S20. f) ^IPNS$^{S154C/S323C}$; (g) ^IPNS$^{S154C/S323C}$ with an excess of Cd(II) (5 eq., 600 μM); (h) ^IPNS$^{S154C/S323C}$:Cd(II) with an excess of ACV (64 eq., 7.68 mM); (i) ^IPNS$^{S154C/S323C}$:Cd(II):ACV exposed to NO (1000 ppm in N_2) for 45 min.

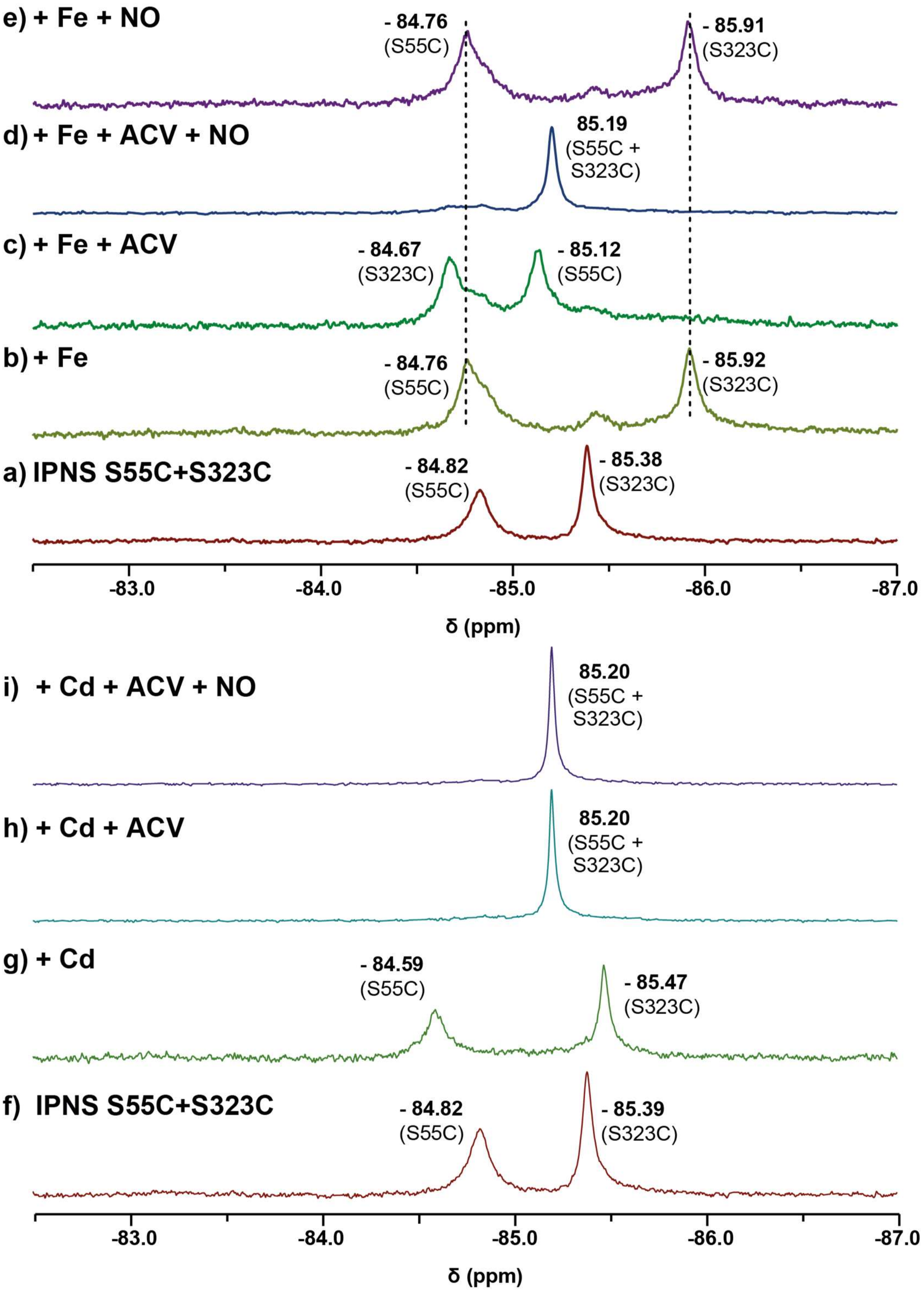

Figure 8. ^{19}F NMR spectra of reactions of double ^{19}F labelled IPNS$^{S55C/S323C}$ with Fe(II), Cd(II) and ACV, and exposure to nitric oxide (NO). a) IPNS$^{S55C/S323C}$ (120 μM) and CF_3CO_2H (100 μM) in Tris-d_{11} (25 mM, in H_2O, pH 7.5) and D_2O (50 μL, 10% (v/v)) (this

mixture is referred to as ^IPNS$^{S55C/S323C}$); (b) ^IPNS$^{S55C/S323C}$ with an excess of Fe(II) (5 eq., 600 μM); (c) ^IPNS$^{S55C/S323C}$:Fe(II) with an excess of ACV (64 eq., 7.68 mM); (d) ^IPNS$^{S55C/S323C}$:Fe(II):ACV exposed to NO (1000 ppm in N_2) for 30 min: (e) ^IPNS$^{S55C/S323C}$:Fe(II) exposed to NO (1000 ppm in N_2) for 45 min.. f) ^IPNS$^{S55C/S323C}$; (g) ^IPNS$^{S55C/S323C}$ with an excess of Cd(II) (5 eq., 600 μM); (h) ^IPNS$^{S55C/S323C}$:Cd(II) with an excess of ACV (64 eq., 7.68 mM); (i) ^IPNS$^{S55C/S323C}$:Cd(II):ACV exposed to NO (1000 ppm in N_2) for 45 min.

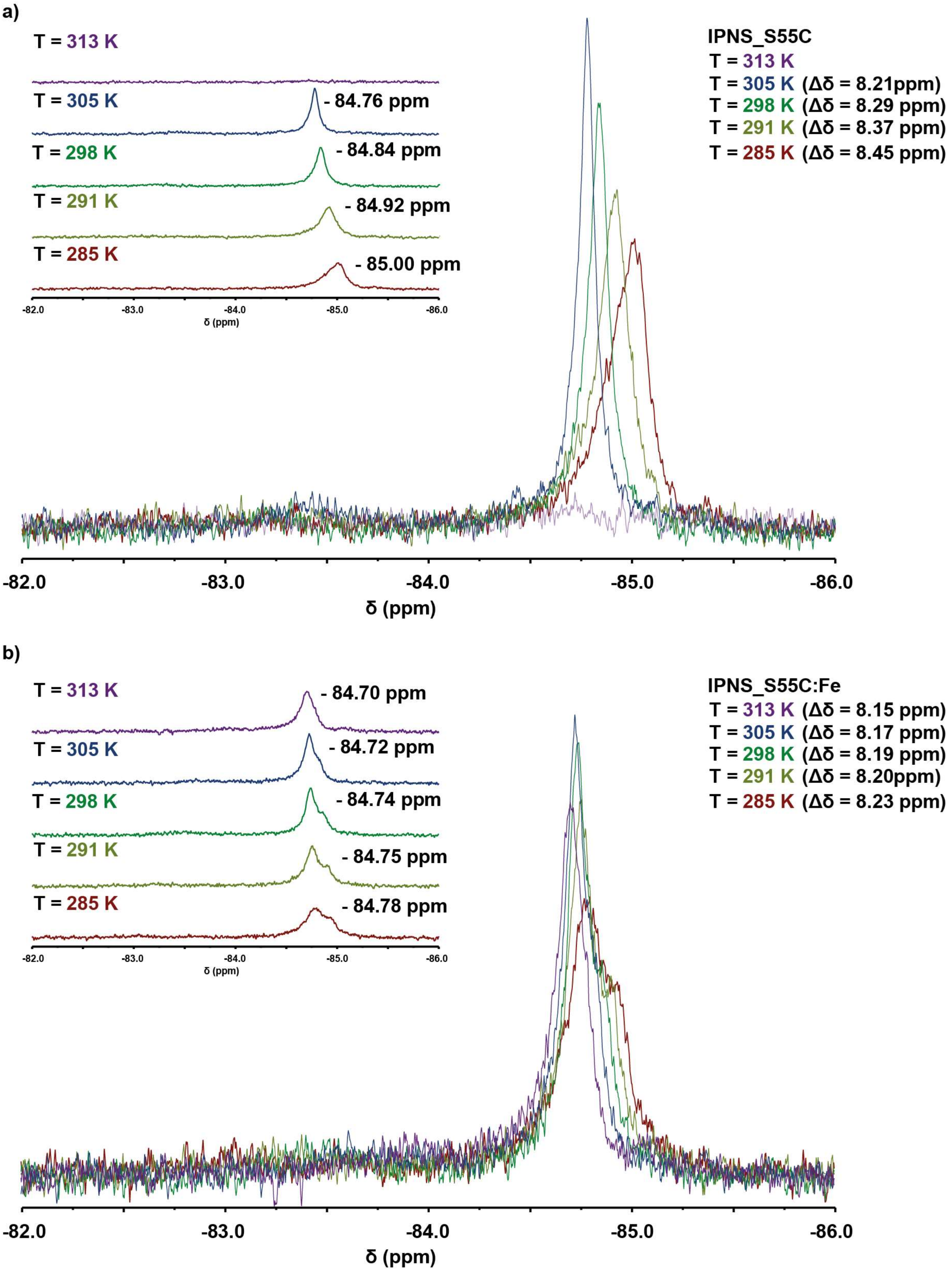

Figure 9. ^{19}F NMR spectra of ^{19}F labelled IPNS^{S55C} ± Fe(II) at different temperatures. a) IPNS^{S55C} (IPNS 120 μM) and CF_3CO_2H (100 μM) in Tris-d$_{11}$ (25 mM, in H_2O, pH 7.5) and D_2O (50 μL, 10% (v/v)) at 285 K (red); at 291 K (olive green); 298 K (green): 305 K (blue) and 313 K (purple). b) IPNS^{S55C}:Fe (IPNS 120 μM; Fe(II) 5 eq., 600 μM) and CF_3CO_2H (100 μM) in Tris-d$_{11}$ (25 mM, in H_2O, pH 7.5) and D_2O (50 μL, 10% (v/v)) at 285 K (red); at 291 K (olive green); 298 K (green): 305 K (blue) and 313 K (purple). Note: ^{19}F NMR spectra

were re-measured at 298 K after heating to 313 K (data not shown). These spectra indicate that *(a)* there is complete decomposition of IPNS^{S55C}, but that for *(b)* IPNS^{S55C}:Fe there is no discernible decomposition.

a)

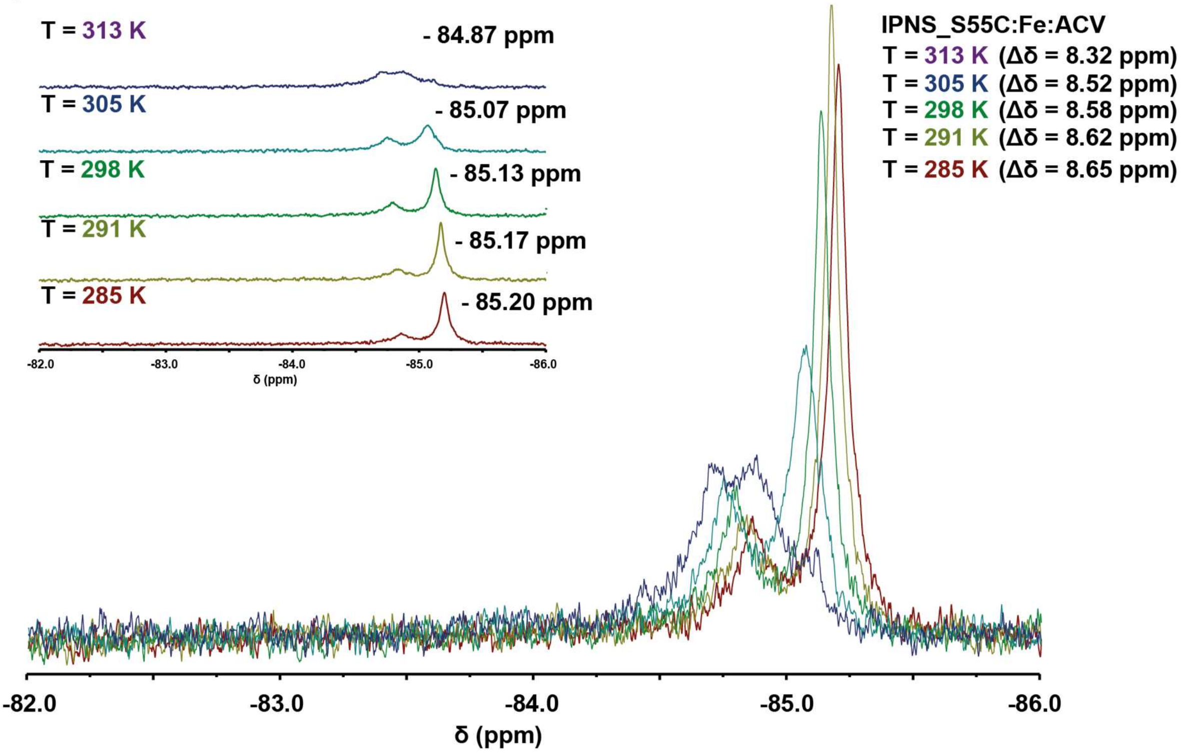

b)

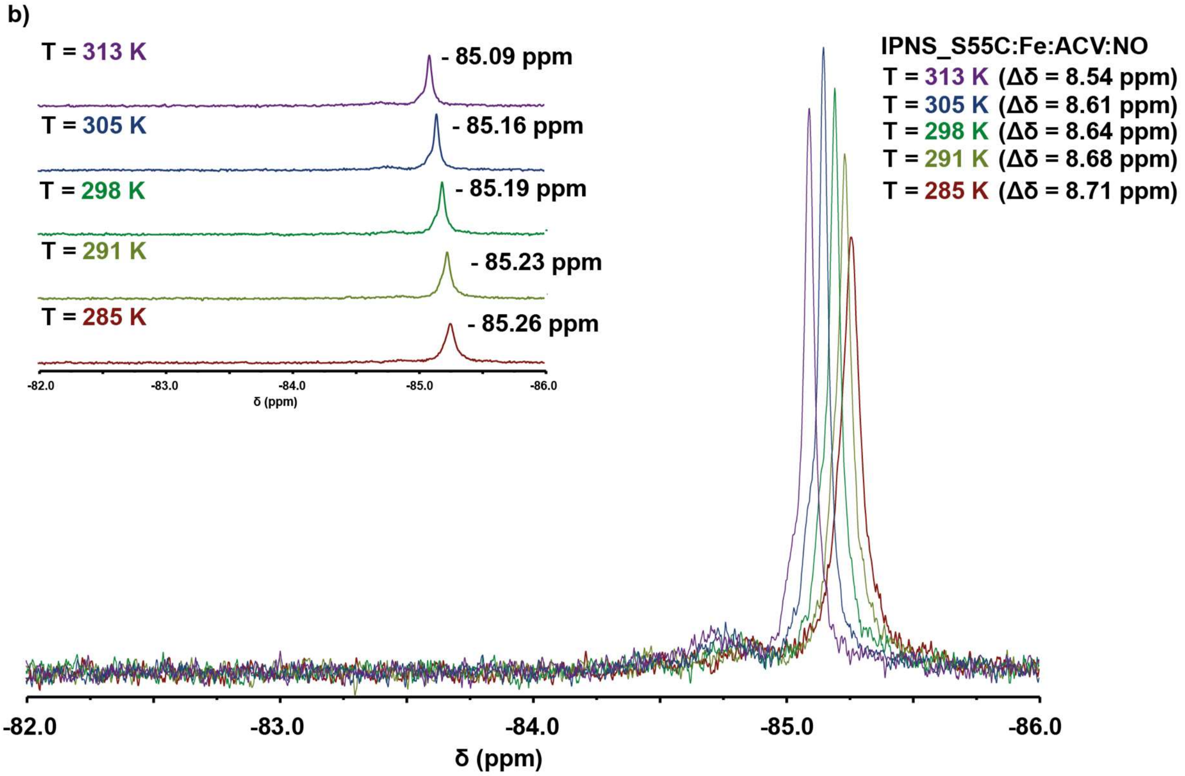

Figure 10. ^{19}F NMR spectra of ^{19}F labelled IPNS^{S55C} with Fe(II) and ACV ± nitric oxide (NO) at different temperatures revealing evidence for different conformations of the dynamic α3 helix. a) IPNS^{S55C}:Fe:ACV (IPNS 120 μM; Fe(II) 5 eq., 600 μM; ACV 64 eq., 7.68 mM) and CF_3CO_2H (100 μM) in Tris-d_{11} (25 mM, in H_2O, pH 7.5) and D_2O (50 μL, 10% (v/v)) at 285 K (red); at 291 K (olive green); 298 K (green): 305 K (blue) and 313 K (purple). b) IPNS^{S55C}:Fe:ACV:NO (IPNS 120 μM; Fe(II) 5 eq., 600 μM; ACV 64 eq., 7.68 mM: 45 min NO exposure (1000 ppm in N_2)(1) and CF_3CO_2H (100 μM) in Tris-d_{11} (25 mM, in H_2O, pH 7.5) and D_2O (50 μL, 10% (v/v)) at 285 K (red); at 291 K (olive green); 298 K (green): 305 K (blue) and 313 K (purple). Note: ^{19}F NMR spectra were re-measured at 298 K after heating to 313 K (data not shown). These spectra indicate a lack of decomposition of *(a)* IPNS^{S55C}:Fe:ACV and *(b)* IPNS^{S55C}:Fe:ACV:NO.

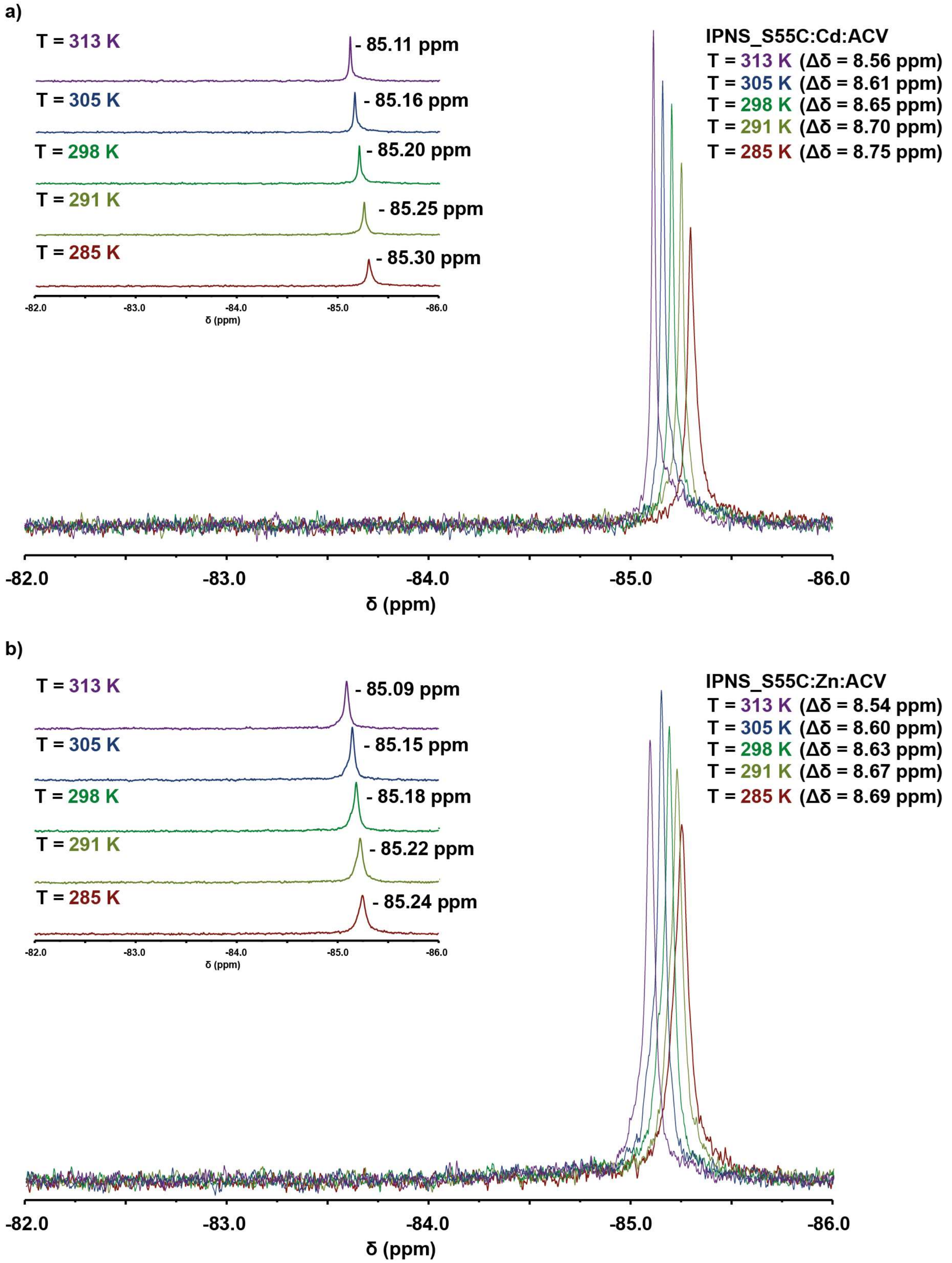

Figure 11. ^{19}F NMR spectra of ^{19}F labelled IPNS^{S55C} with Cd(II)/Zn(II) and ACV at different temperatures. a) IPNS^{S55C}:Cd:ACV (IPNS 120 μM; Cd(II) 5 eq., 600 μM; ACV 64 eq., 7.68 mM) and CF_3CO_2H (100 μM) in Tris-d_{11} (25 mM, in H_2O, pH 7.5) and D_2O (50 μL, 10% (v/v)) at 285 K (red); at 291 K (olive green); 298 K (green): 305 K (blue) and 313 K (purple). a) IPNS^{S55C}:Zn:ACV:NO (IPNS 120 μM; Zn(II) 5 eq., 600 μM; ACV 64 eq., 7.68 mM) and CF_3CO_2H (100 μM) in Tris-d_{11} (25 mM, in H_2O, pH 7.5) and D_2O (50 μL, 10% (v/v))

at 285 K (red); at 291 K (olive green); 298 K (green): 305 K (blue) and 313 K (purple). Note: ^{19}F NMR spectra were re-measured at 298 K after heating to 313 K (data not shown). These spectra indicate a lack of decomposition of *(a)* IPNS^{S55C}:Cd:ACV and *(b)* IPNS^{S55C}:Zn:ACV.

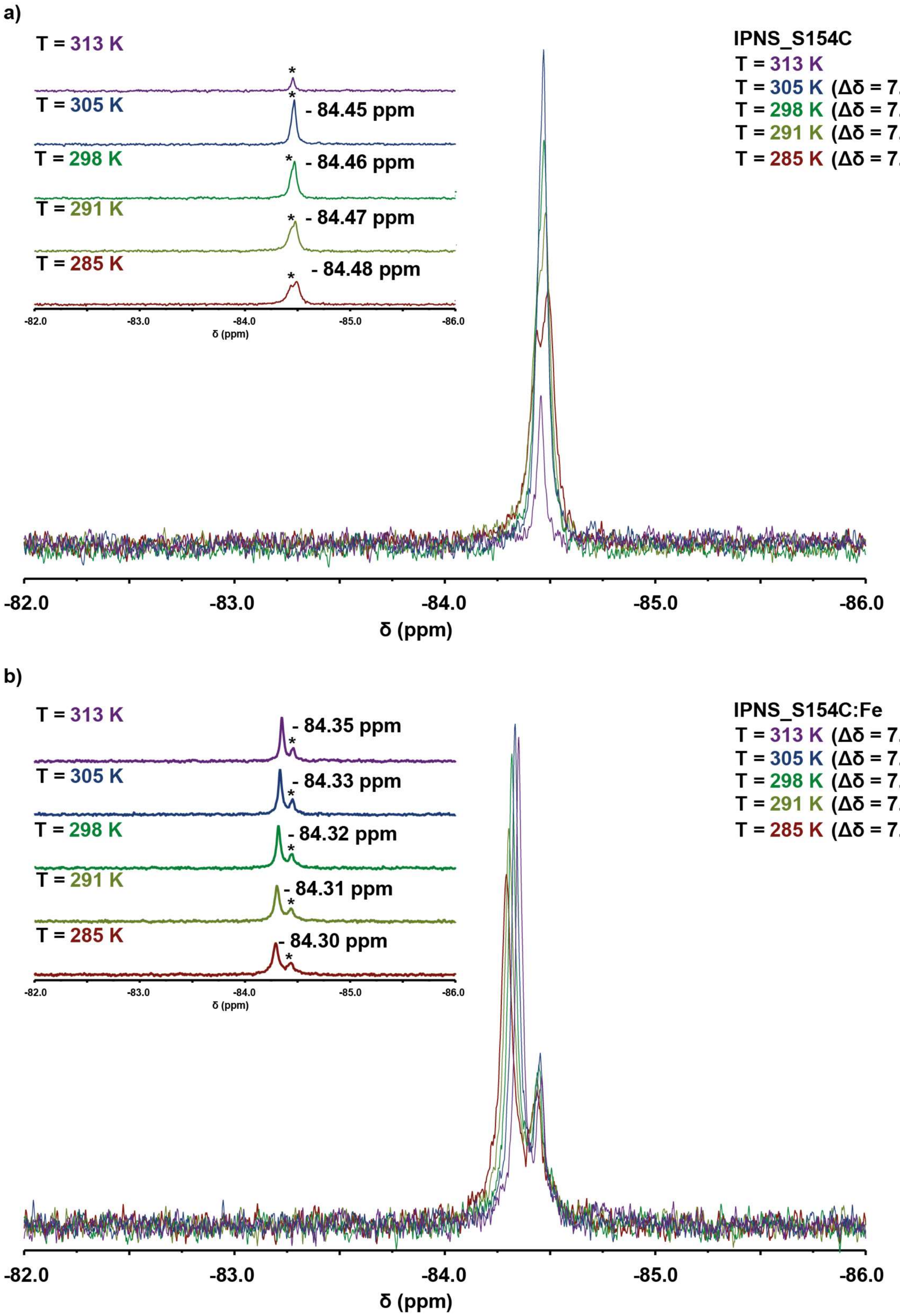

Figure 12. ^{19}F NMR spectra of ^{19}F labelled IPNS^{S154C} ± Fe(II) at different temperatures. a) IPNS^{S154C} (IPNS 120 μM) with CF_3CO_2H (100 μM) in Tris-d_{11} (25 mM, in H_2O, pH 7.5)

and D_2O (50 µL, 10% (v/v)) at 285 K (red); at 291 K (olive green); 298 K (green): 305 K (blue) and 313 K (purple). ^{19}F NMR spectra were re-measured at 298 K after heating to 313 K implied complete decomposition of the sample. The * labelled peak is likely a contaminant. b) IPNS^{S154C}:Fe (IPNS 120 µM; Fe(II) 5 eq., 600 µM) with CF_3CO_2H (100 µM) in Tris-d_{11} (25 mM, in H_2O, pH 7.5) and D_2O (50 µL, 10% (v/v)) at 285 K (red); at 291 K (olive green); 298 K (green): 305 K (blue) and 313 K (purple).

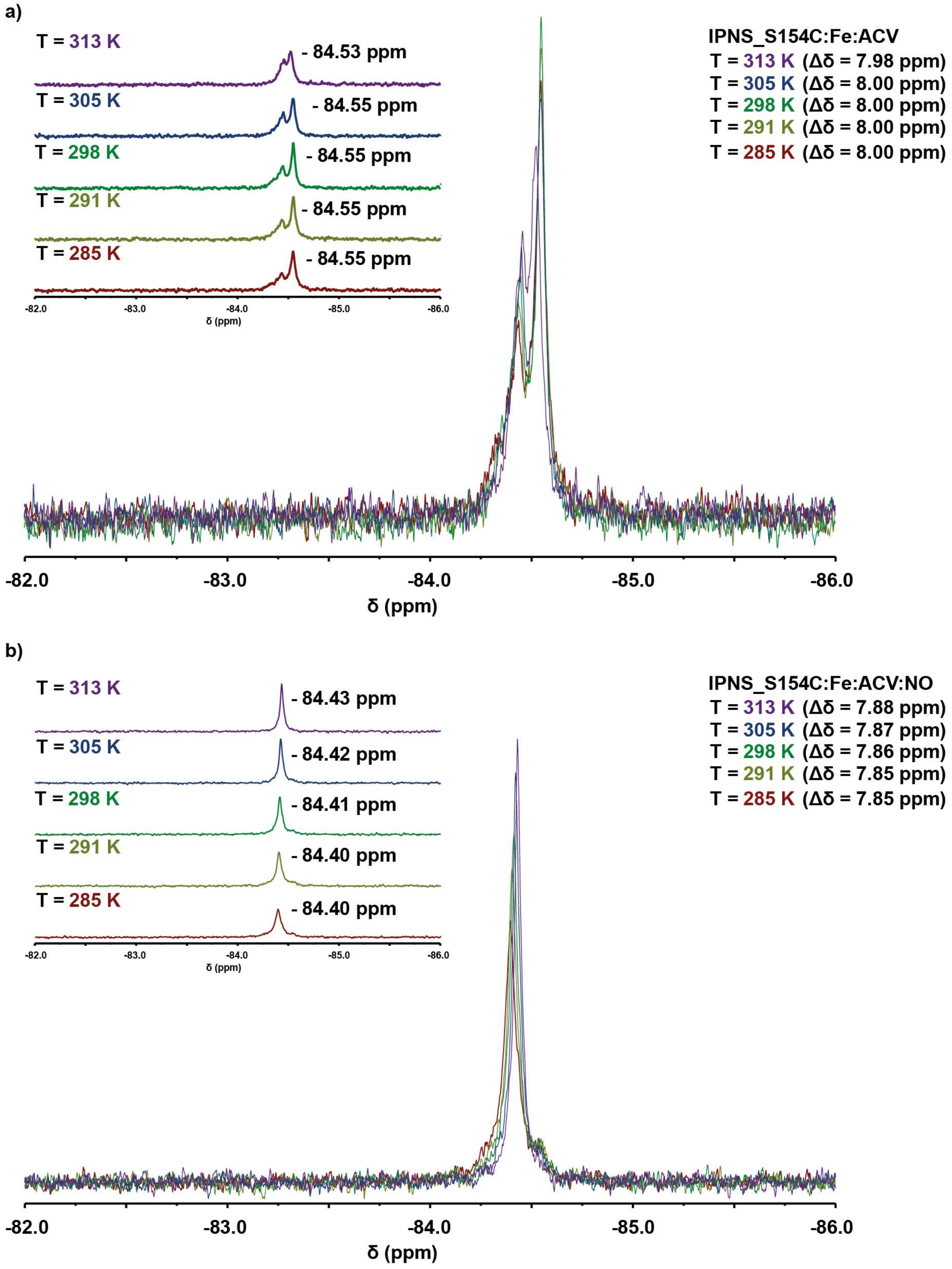

Figure 13. ^{19}F NMR spectra of ^{19}F labelled $IPNS^{S154C}$ with Fe(II) and ACV ± nitric oxide (NO) at different temperatures. a) $IPNS^{S154C}$:Fe:ACV (IPNS 120 μM; Fe(II) 5 eq., 600 μM; ACV 64 eq., 7.68 mM) and CF_3CO_2H (100 μM) in Tris-d_{11} (25 mM, in H_2O, pH 7.5) and D_2O (50 μL, 10% (v/v)) at 285 K (red); at 291 K (olive green); 298 K (green): 305 K (blue) and 313 K (purple). b) $IPNS^{S154C}$:Fe:ACV:NO (IPNS 120 μM; Fe(II) 5 eq., 600 μM; ACV 64 eq., 7.68 mM: 45 min NO exposure (1000 ppm in N_2) (1) and CF_3CO_2H (100 μM) in Tris-d_{11} (25 mM,

in H_2O, pH 7.5) and D_2O (50 μL, 10% (v/v)) at 285 K (red); at 291 K (olive green); 298 K (green): 305 K (blue) and 313 K (purple). Note: ^{19}F NMR spectra were re-measured at 298 K after heating to 313 K (data not shown). The resultant spectra indicate no evidence for decomposition of the sample.

a)

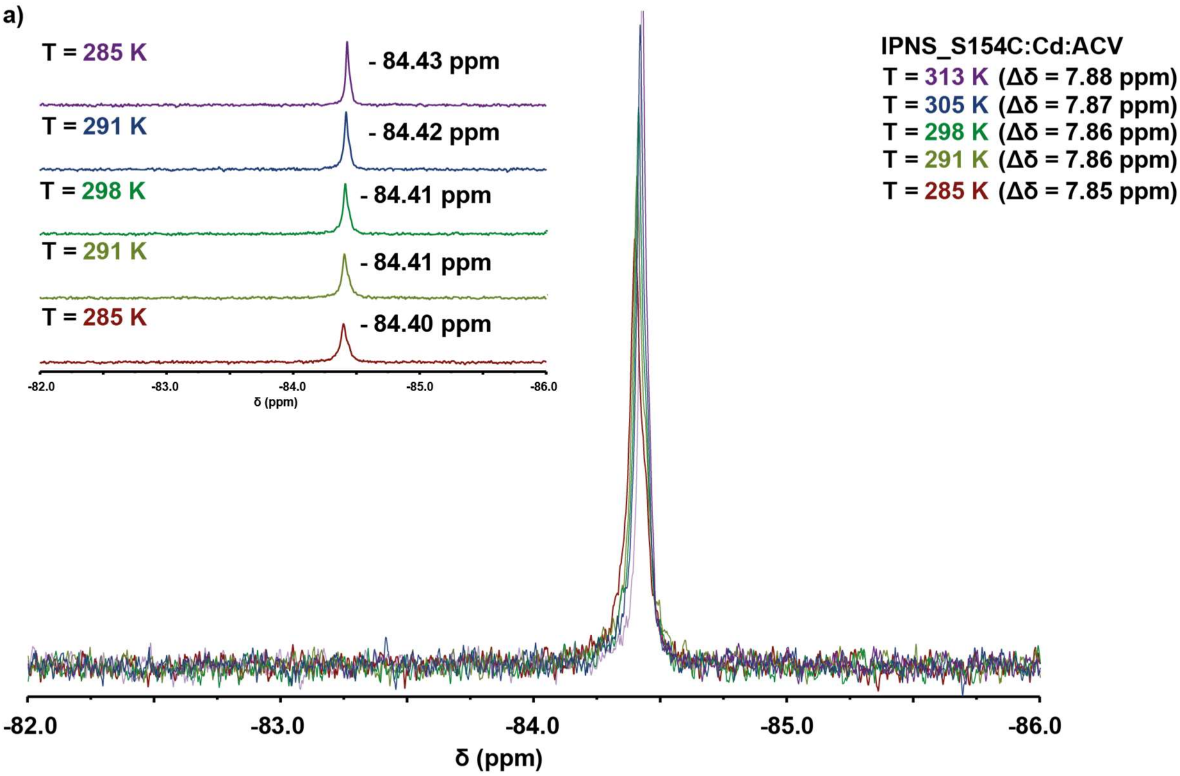

b)

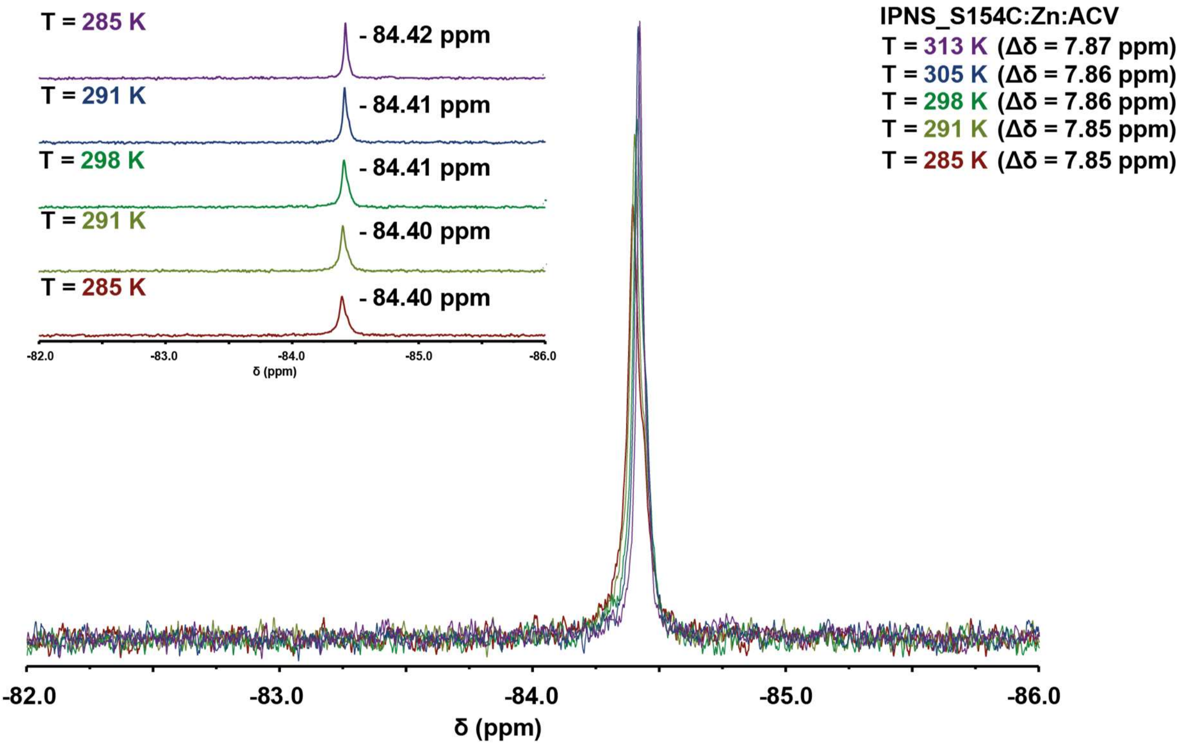

Figure 14. ^{19}F NMR spectra of ^{19}F labelled $IPNS^{S154C}$ with Cd(II)/Zn(II) and ACV at different temperatures. a) $IPNS^{S154C}$:Cd:ACV (IPNS 120 μM; Cd(II) 5 eq., 600 μM; ACV

64 eq., 7.68 mM) and CF_3CO_2H (100 μM) in Tris-d_{11} (25 mM, in H_2O, pH 7.5) and D_2O (50 μL, 10% (v/v)) at 285 K (red); at 291 K (olive green); 298 K (green): 305 K (blue) and 313 K (purple). b) $IPNS^{S154C}$:Zn:ACV:NO (IPNS 120 μM; Zn(II) 5 eq., 600 μM; ACV 64 eq., 7.68 mM) with CF_3CO_2H (100 μM) in Tris-d_{11} (25 mM, in H_2O, pH 7.5) and D_2O (50 μL, 10% (v/v)) at 285 K (red); at 291 K (olive green); 298 K (green): 305 K (blue) and 313 K (purple). Note: ^{19}F NMR spectra were repeated at 298 K after heating to 313 K. The resultant spectra indicate no evidence for decomposition of *(a)* $IPNS^{S154C}$:Cd:ACV and *(b)* $IPNS^{S154C}$:Zn:ACV.

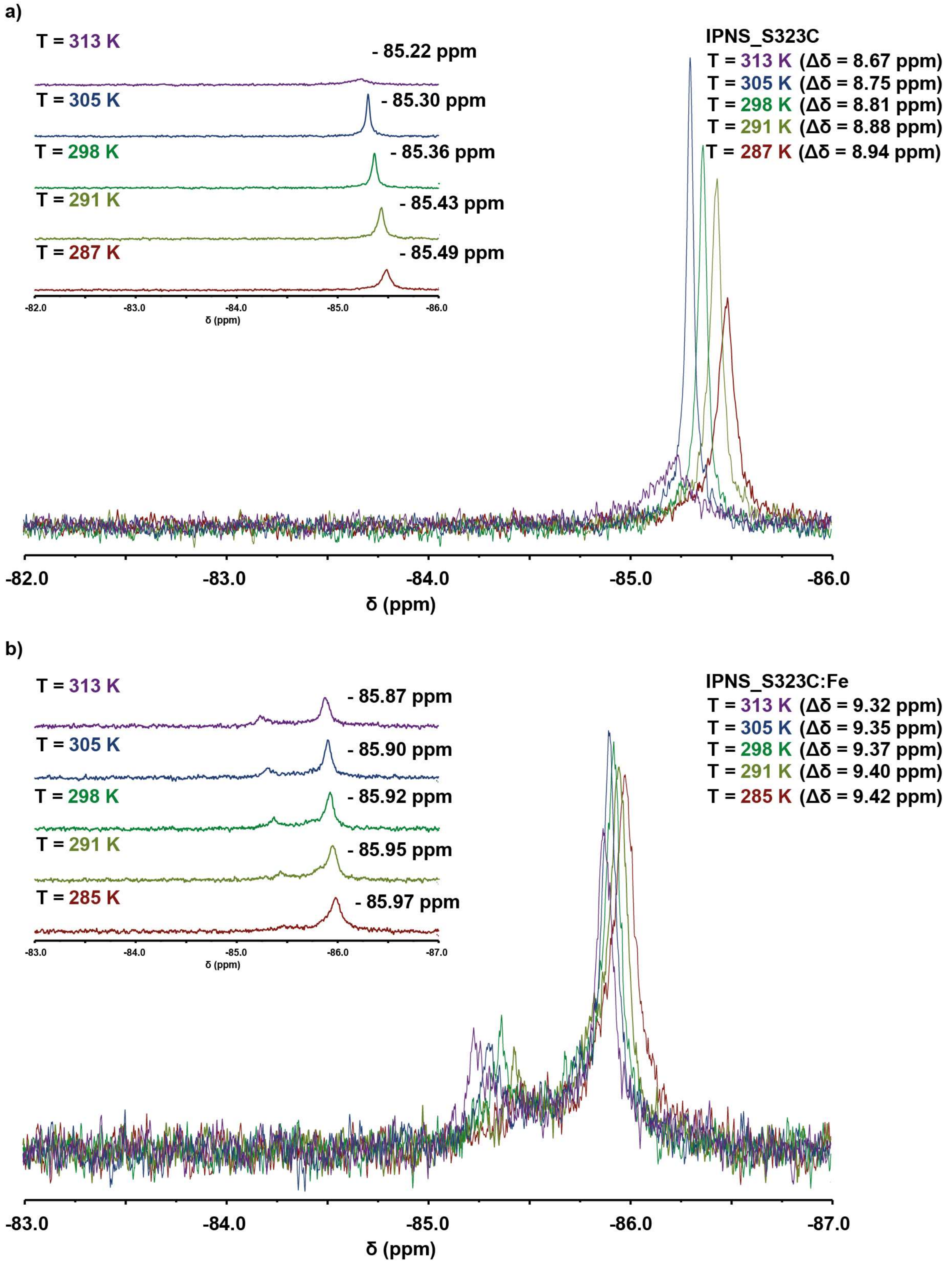

Figure 15. ^{19}F NMR spectra of ^{19}F labelled IPNS^{S323C} ± Fe(II) at different temperatures a) IPNS^{S323C} (IPNS 120 μM) and CF_3CO_2H (100 μM) in Tris-d_{11} (25 mM, in H_2O, pH 7.5) and D_2O (50 μL, 10% (v/v)) at 285 K (red); at 291 K (olive green); 298 K (green): 305 K (blue) and 313 K (purple). b) IPNS^{S323C}:Fe (IPNS 120 μM; Fe(II) 5 eq., 600 μM) and CF_3CO_2H (100 μM) in Tris-d_{11} (25 mM, in H_2O, pH 7.5) and D_2O (50 μL, 10% (v/v)) at 285 K (red); at 291 K (olive green); 298 K (green): 305 K (blue) and 313 K (purple). Note, the ^{19}F NMR

spectra were re-measured at 298 K after heating to 313 K. The resultant spectra indicate that *(a)* there is almost complete decomposition of IPNS^{S323C}, but that for *(b)* IPNS^{S323C}:Fe there is only slight decomposition.

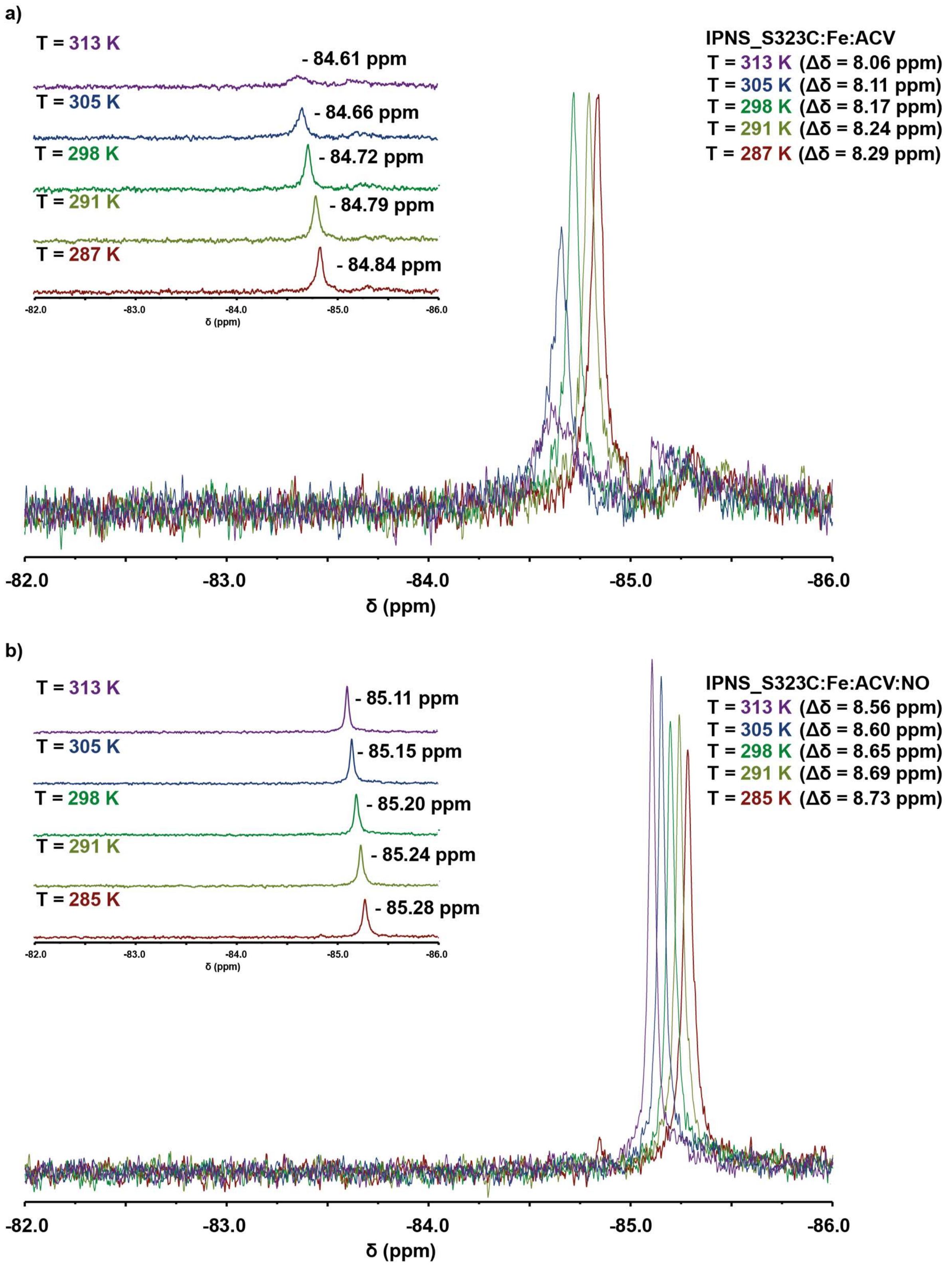

Figure 16. ^{19}F NMR spectra of ^{19}F labelled IPNS^{S323C} with Fe(II) and ACV ± nitric oxide (NO) at different temperatures revealing the different conformations of the dynamic α10 helix. a) IPNS^{S323C}:Fe:ACV (IPNS 120 μM; Fe(II) 5 eq., 600 μM; ACV 64 eq., 7.68 mM) and CF_3CO_2H (100 μM) in Tris-d_{11} (25 mM, in H_2O, pH 7.5) and D_2O (50 μL, 10% (v/v)) at 285 K (red); at 291 K (olive green); 298 K (green): 305 K (blue) and 313 K (purple). b) IPNS^{S323C}:Fe:ACV:NO (IPNS 120 μM; Fe(II) 5 eq., 600 μM; ACV 64 eq., 7.68 mM: 45 min NO exposure (1000 ppm in N_2)(1) and CF_3CO_2H (100 μM) in Tris-d_{11} (25 mM, in H_2O, pH 7.5) and D_2O (50 μL, 10% (v/v)) at 285 K (red); at 291 K (olive green); 298 K (green): 305 K (blue) and 313 K (purple). Note: ^{19}F NMR spectra were re-measured at 298 K after heating to 313 K. The resultant spectra indicate no evidence for decomposition for *(a)* IPNS^{S323C}:Fe:ACV and *(b)* IPNS^{S323C}:Fe:ACV:NO.

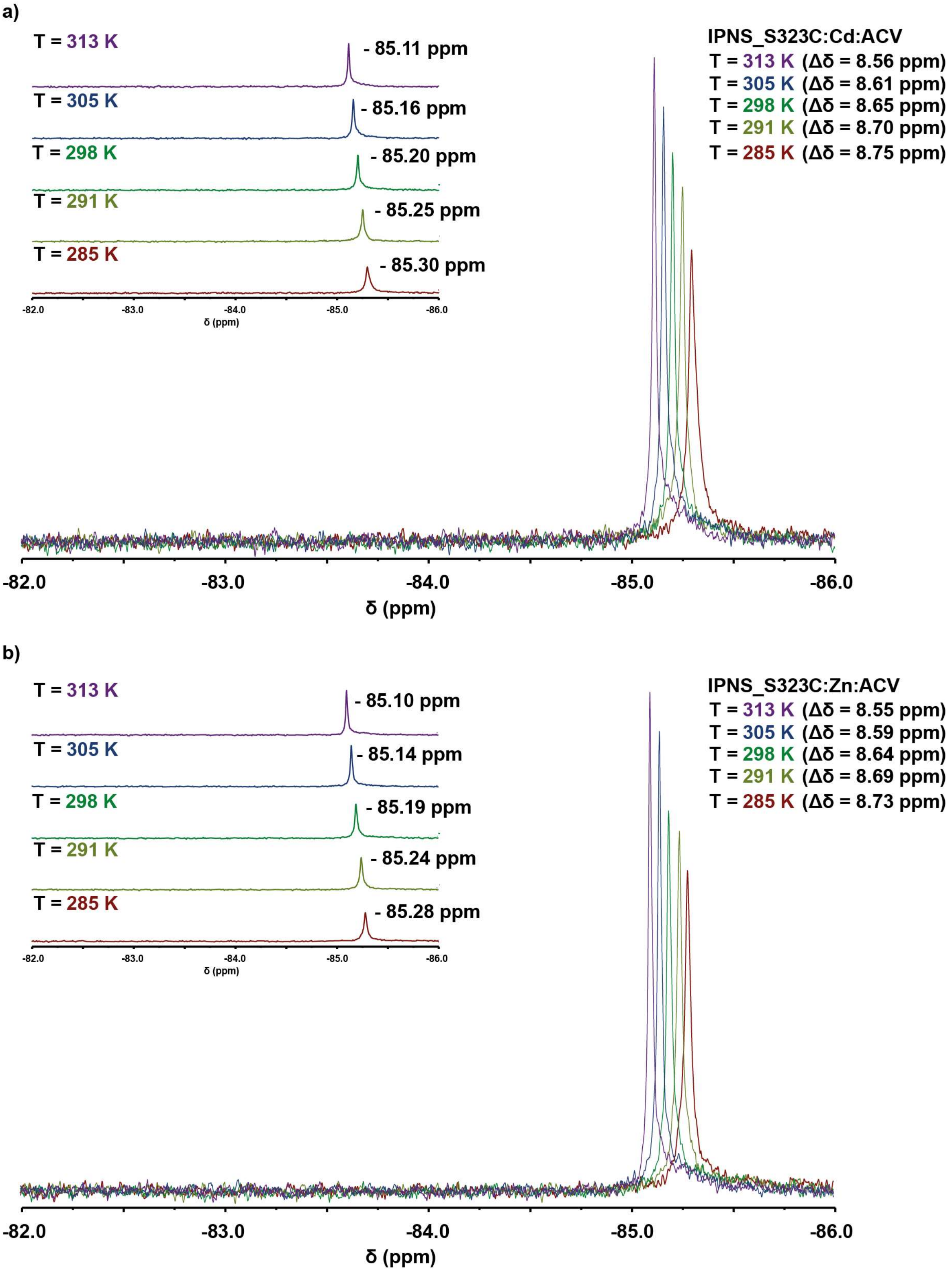

Figure 17. ^{19}F NMR spectra of ^{19}F labelled IPNS^{S323C} with Cd(II)/Zn(II) and ACV at different temperatures. a) IPNS^{S323C}:Cd:ACV (IPNS 120 μM; Cd(II) 5 eq., 600 μM; ACV 64 eq., 7.68 mM) and CF_3CO_2H (100 μM) in Tris-d$_{11}$ (25 mM, in H_2O, pH 7.5) and D_2O (50 μL, 10% (v/v)) at 285 K (red); at 291 K (olive green); 298 K (green): 305 K (blue) and 313 K (purple). b) IPNS^{S323C}:Zn:ACV:NO (IPNS 120 μM; Zn(II) 5 eq., 600 μM; ACV 64 eq., 7.68 mM) with CF_3CO_2H (100 μM) in Tris-d$_{11}$ (25 mM, in H_2O, pH 7.5) and D_2O (50 μL, 10% (v/v)) at 285 K (red); at 291 K (olive green); 298 K (green): 305 K (blue) and 313 K (purple).

Note: ^{19}F NMR spectra were re-measured at 298 K after heating to 313 K. The resultant spectra indicate that there is for *(a)* IPNS^{S323C}:Cd:ACV no and for *(b)* IPNS^{S323C}:Zn:ACV:NO slight decomposition.

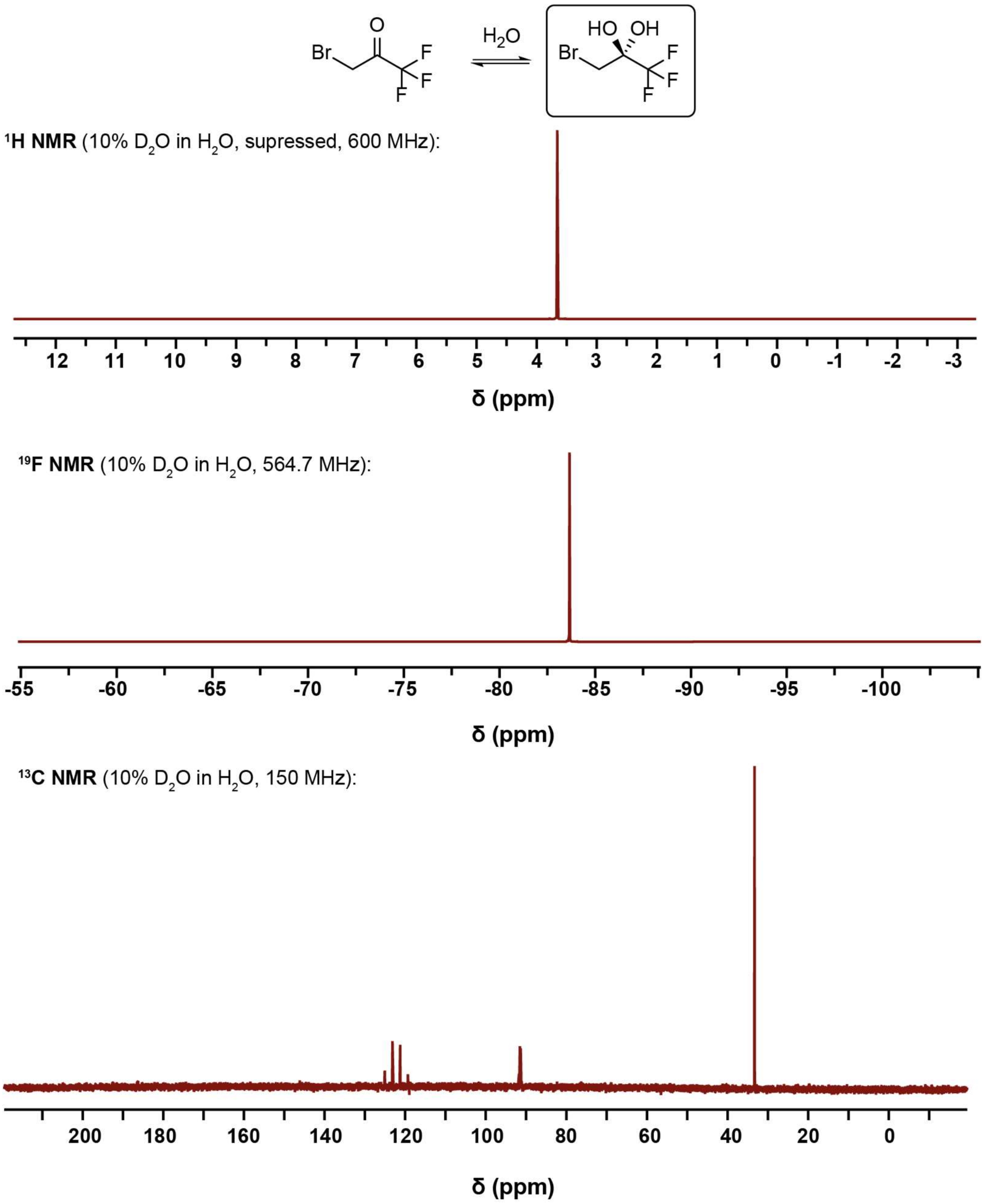

Figure 18. 3,3,3-Trifluoropropane-1,2,2-triol. ^{1}H NMR (600 MHz, D_2O) δ: 3.68 (s, 2H, CH_2) ppm. ^{19}F NMR (564.7 MHz, D_2O) δ: -83.3 ppm. ^{13}C NMR (150.9 MHz, D_2O) δ: 123.1 (q, $^{1}J_{C,F}$ = 287.2 Hz), 92.4 (q, $^{2}J_{C,F}$ = 30.9 Hz), 62.6 ppm.

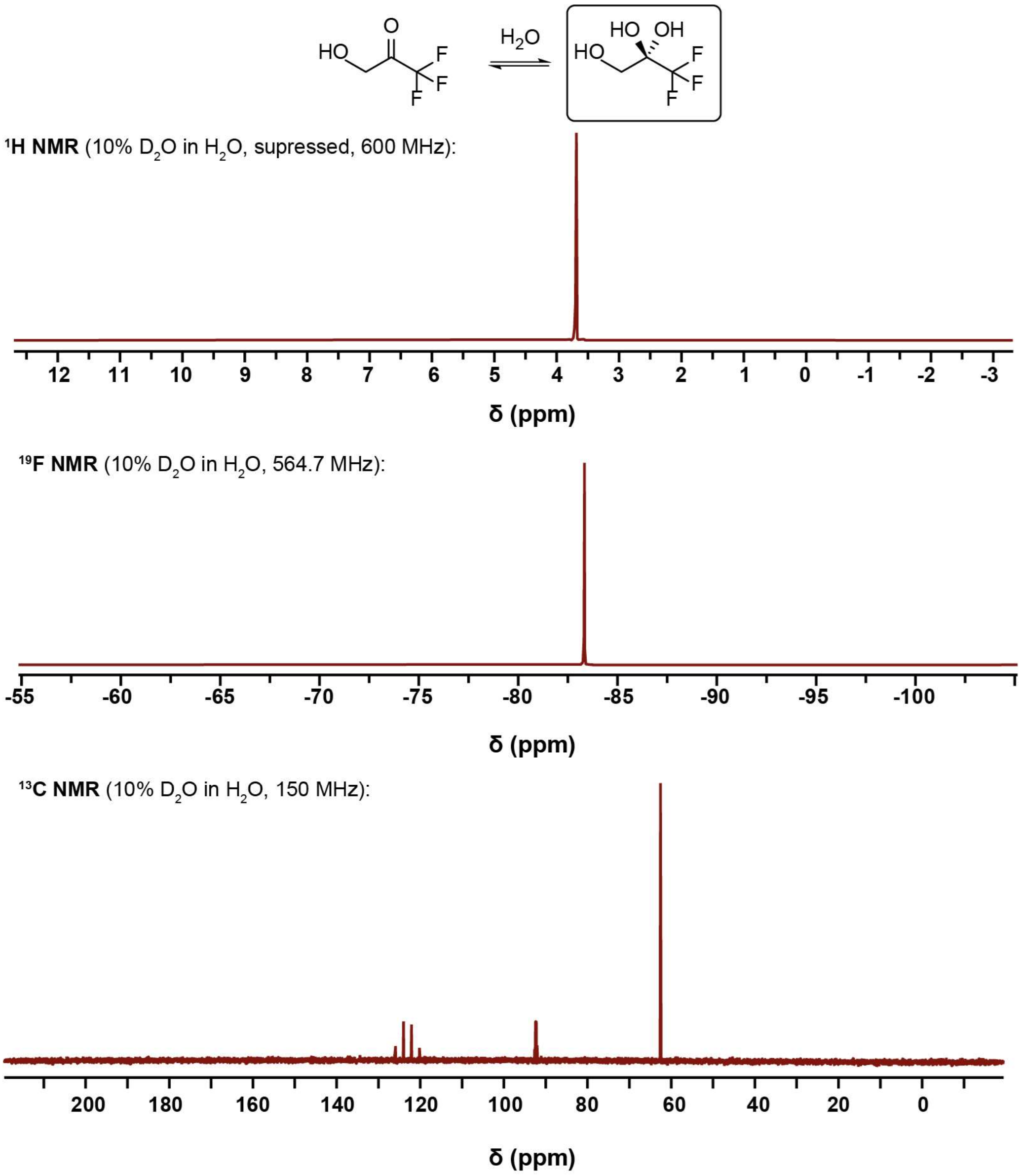

Figure 19. 3-Bromo-1,1,1-trifluoropropane-2,2-diol. ^{1}H NMR (600 MHz, D_2O) δ: 3.66 (s, 2H, CH_2) ppm. ^{19}F NMR (564.7 MHz, D_2O) δ: -83.7 ppm. ^{13}C NMR (150.9 MHz, D_2O) δ: 122.1 (q, $^1J_{C,F}$ = 288.1 Hz), 91.4 (q, $^2J_{C,F}$ = 32.0 Hz), 33.3 ppm.

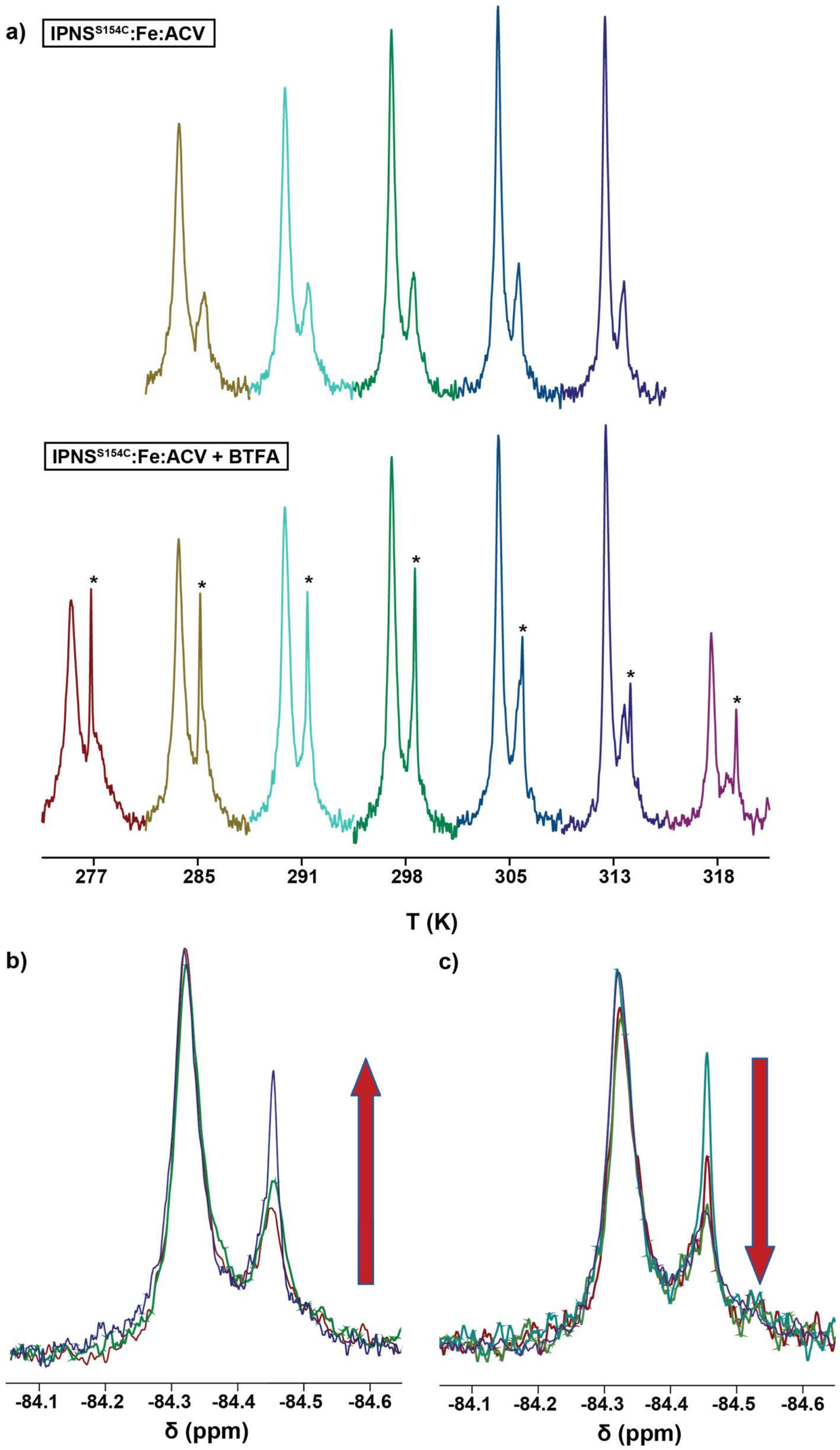

Figure 20. ^{19}F NMR-observed comparison of a minor peak of ^{19}F labelled IPNS^{S154C}:Fe(II) and 'free' hydrolysed BTFA. These studies were carried out to determine if the sharp minor peak of ^IPNS^{S154C}:Fe (and some other conformations) was formed by 3,3,3-trifluoropropane-1,2,2-triol, which is formed by hydrolysis of BTFA. a) Temperature series: The lower spectra were recorded on IPNS^{S154C}:Fe (IPNS 120 μM) with CF_3CO_2H (100 μM) in Tris-d_{11} (25 mM, in H_2O, pH 7.5) and D_2O (50 μL, 10% (v/v)) (this mixture is referred to as ^IPNS^{S154C}:Fe) at 277 K (red); 285 K (gold); 291 K (olive green); 298 K (green): 305 K (blue); 313 K (purple) and 318 K (magenta). The upper spectra are

from Figure 11b and are shown for comparison in absence of hydrolysed BTFA, 3,3,3-trifluoropropane-1,2,2-triol. b) Titration of 3,3,3-trifluoropropane-1,2,2-triol into ^IPNS^{S154C}:Fe solution. IPNS^{S154C}:Fe (red); ^IPNS^{S154C}:Fe with 1 μM 3,3,3-trifluoropropane-1,2,2-triol (green); ^IPNS^{S154C}:Fe with 3 μM 3,3,3-trifluoropropane-1,2,2-triol (purple). c) 3,3,3-trifluoropropane-1,2,2-triol and IPNS^{S154C}:Fe peaks were differentiated by diffusion experiments with a standard Bruker ledgp2s pulse sequence with a d1 of 2 seconds. ^IPNS^{S154C}:Fe:BTFA with a gradient pulse of 2% (teal); 60% (brown) and 80% (green). ^IPNS^{S154C}:Fe using a standard Bruker znflqn pulse sequence in the absence of BTFA (purple) for comparison.

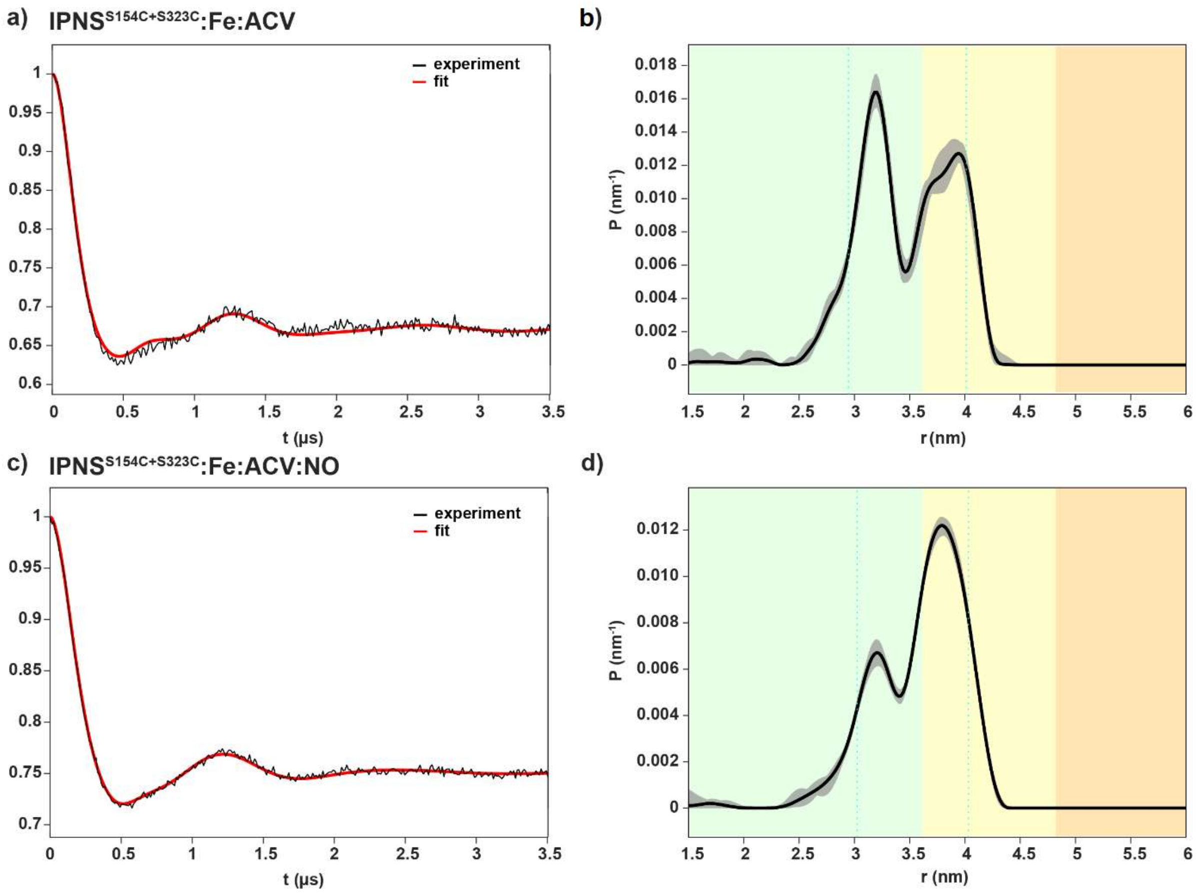

Figure 21: DEER experiments with IPNS$^{S154C+S323C}$:Fe:ACV under anaerobic and NO exposed conditions. Samples of spin labelled IPNS$^{S154C+S323C}$:Fe:ACV (IPNS 150 µM; Fe(II) 1 eq., 150 µM; ACV 32 eq., 4.80 mM in EPR buffer (Tris 25 mM, 200 mM NaCl, in D_2O, pH 8.0) and 30 % (v/v) glycerol-d_8) were prepared and exposed to NO (1000 ppm in N_2, 30 min) to obtain spin-labelled IPNS$^{S154C+S323C}$:Fe:ACV:NO. a) Comparison of background-corrected experimental data and fitted model of IPNS$^{S154C+S323C}$:Fe:ACV. b) Distance distribution derived from experimental data of doubly spin labelled IPNS$^{S154C+S323C}$:Fe:ACV revealing a major distance distribution between both spin labels of 3.19 nm, and a minor distance distribution of 3.95 nm. c) Comparison of background-corrected experimental data and fitted model of IPNS$^{S154C+S323C}$:Fe:ACV:NO. d) Distance distributions derived from experimental data of doubly spin labelled IPNS$^{S154C+S323C}$:Fe:ACV:NO revealing two major distance distributions between both spin labels of 3.21 nm and 3.80 nm, with a substantial increase of the larger distance distribution compared to the doubly spin labelled IPNS$^{S154C+S323C}$:Fe:ACV complex. In each case, time traces were truncated by one microsecond to remove [2+1] artefacts prior to analysis, distance distributions were optimised using Tikhonov regularisation, and the optimal regularisation parameter (α) was chosen using automatic L-curve corner recognition.(3)

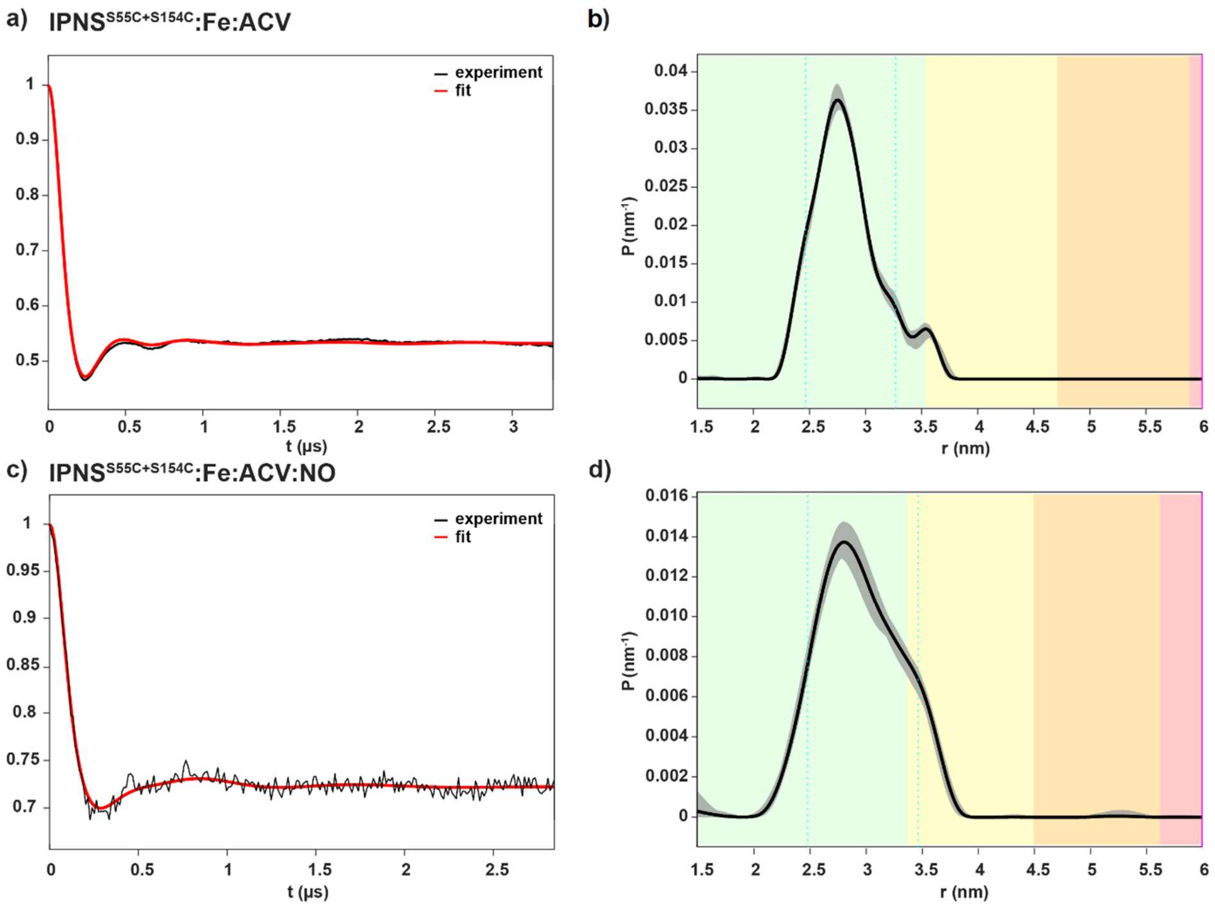

Figure 22: DEER experiments with IPNS$^{S55C+S154C}$:Fe:ACV under anaerobic and NO exposed conditions. Samples of spin labelled IPNS$^{S55C+S154C}$:Fe:ACV (IPNS 150 µM; Fe(II) 1 eq., 150 µM; ACV 32 eq., 4.80 mM in EPR buffer (Tris 25 mM, 200 mM NaCl, in D_2O, pH 8.0) and 30 % (v/v) glycerol-*d*$_8$) were prepared and exposed to NO (1000 ppm in N_2, 30 min) to obtain spin-labelled IPNS$^{S55C+S154C}$:Fe:ACV:NO. a) Comparison of background-corrected experimental data and fitted model of IPNS$^{S55C+S154C}$:Fe:ACV. b) Distance distribution derived from experimental data of doubly spin labelled IPNS$^{S55C+S154C}$:Fe:ACV revealing a major distance distribution between both spin labels of 2.75 nm, and a minor distance distribution of 3.60 nm. c) Comparison of background-corrected experimental data and fitted model of IPNS$^{S55C+S154C}$:Fe:ACV:NO. d) Distance distributions derived from experimental data of doubly spin labelled IPNS$^{S55C+S154C}$:Fe:ACV:NO revealing two major distance distributions between both spin labels of 2.75 nm and a shorter second distance distribution of 3.50 nm compared to the doubly spin labelled IPNS$^{S55C+S154C}$:Fe:ACV complex. Note: The increase in the height of the peak corresponding to higher spin label separation is likely due to movement of α3, although an altering of rotamer populations cannot be fully excluded. In each case, time traces were truncated by one microsecond to remove [2+1] artefacts prior to analysis, distance distributions were optimised using Tikhonov regularisation, and the optimal regularisation parameter (α) was chosen using automatic L-curve corner recognition.(3)

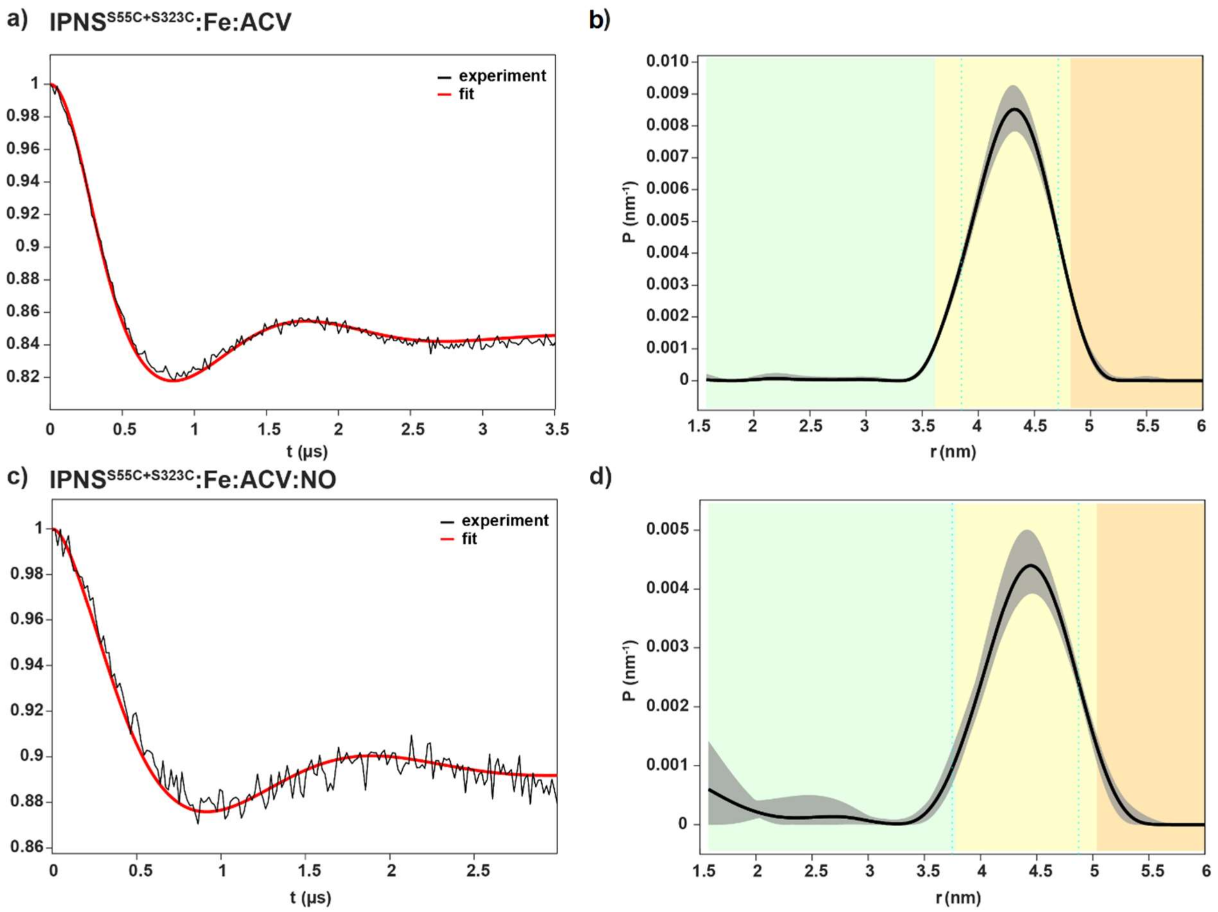

Figure 23: DEER experiments with IPNS$^{S55C+S323C}$:Fe:ACV under anaerobic and NO exposed conditions. Samples of spin labelled IPNS$^{S55C+S323C}$:Fe:ACV (IPNS 150 µM; Fe(II) 1 eq., 150 µM; ACV 32 eq., 4.80 mM in EPR buffer (Tris 25 mM, 200 mM NaCl, in D_2O, pH 8.0) and 30 % (v/v) glycerol-d_8) were prepared and exposed to NO (1000 ppm in N_2, 30 min) to obtain spin-labelled IPNS$^{S55C+S323C}$:Fe:ACV:NO. a) Comparison of background-corrected experimental data and fitted model of IPNS$^{S55C+S323C}$:Fe:ACV. b) Distance distribution derived from experimental data of doubly spin labelled IPNS$^{S55C+S323C}$:Fe:ACV revealing a major distance distribution between both spin labels of 4.33 nm. c) Comparison of background-corrected experimental data and fitted model of IPNS$^{S55C+S323C}$:Fe:ACV:NO. d) Distance distributions derived from experimental data of doubly spin labelled IPNS$^{S55C+S323C}$:Fe:ACV:NO revealing one major distance distribution between both spin labels of 4.45 nm. In each case, time traces were truncated by one microsecond to remove [2+1] artefacts prior to analysis, distance distributions were optimised using Tikhonov regularisation, and the optimal regularisation parameter (α) was chosen using automatic L-curve corner recognition.(3)

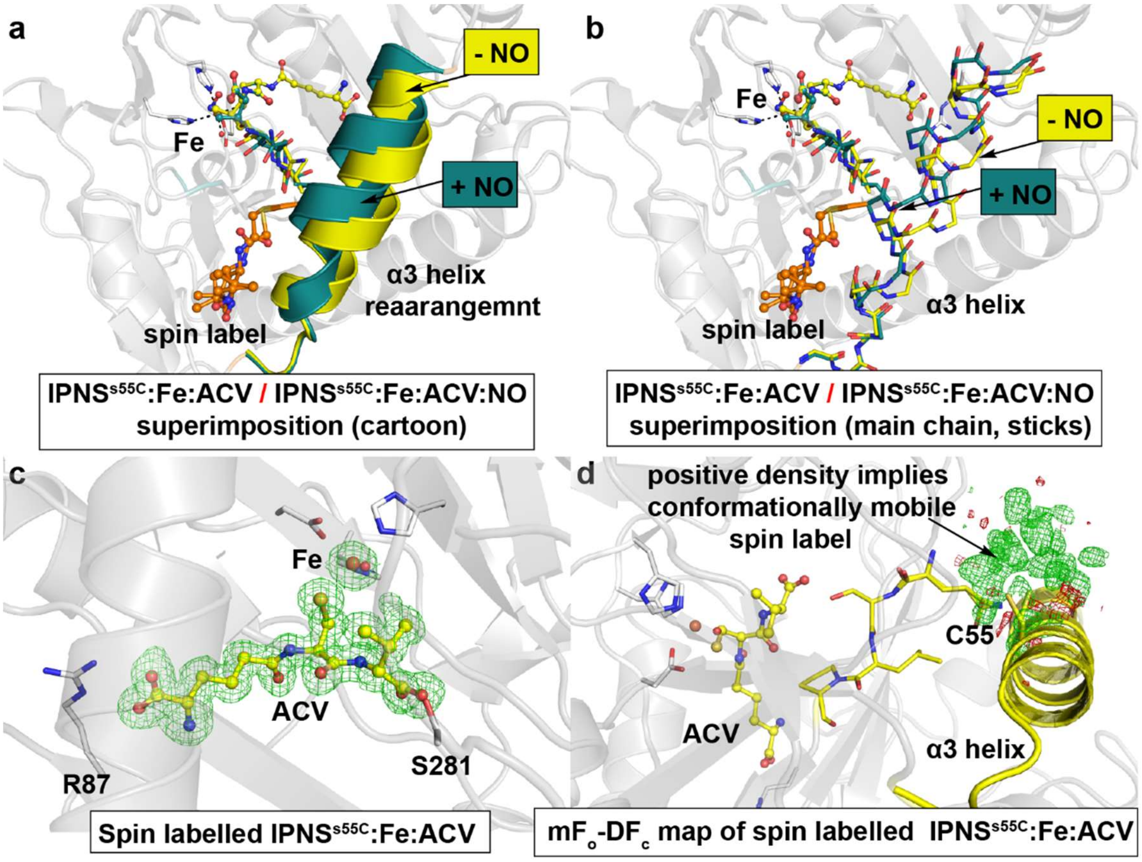

Figure 24. Views from crystal structures of spin labelled IPNS^{S55C}:Fe:ACV (PDB: 7PSW) and spin labelled IPNS^{S55C}:Fe:ACV:NO (PDB: 7POY) revealing rearrangement of α3 on NO binding. Views from a superimposition of spin labelled IPNS^{S55C}:Fe:ACV (PDB: 7PSW, 1.21 Å resolution, yellow) with spin labelled IPNS^{S55C}:Fe:ACV:NO (PDB: 7POY, 1.75 Å resolution, teal) illustrated as a) a cartoon view and b) as sticks only showing the main chain with side chains removed for easier visualization. Spin label: *N*-(1-hydroxy-2,2,5,5-tetramethylpyrrolidin-3-yl)acetamide. c) Polder omit map of spin labelled IPNS^{S55C}:Fe:ACV (PDB: 7PSW) carved around ACV and Fe. d) mF_o-DF_c map of the spin labelled IPNS^{S55C}:Fe:ACV complex (PDB: 7PSW) revealing positive electron density adjacent to Cys55 implying a conformationally mobile spin label on the surface of the protein meaning confident modelling of the spin label was not possible. Note, calculation of isomorphous difference maps between spin labelled IPNS^{S55C}:Fe:ACV and IPNS^{S55C}:Fe:ACV was not possible due to unit cell changes caused by the covalent modification with the spin label.

References:

1. Rabe, P., Kamps, J., Sutherlin, K. D., Linyard, J. D. S., Aller, P., Pham, C. C., Makita, H., Clifton, I., McDonough, M. A., Leissing, T. M., Shutin, D., Lang, P. A., Butryn, A., Brem, J., Gul, S., Fuller, F. D., Kim, I. S., Cheah, M. H., Fransson, T., Bhowmick, A., Young, I. D., O'Riordan, L., Brewster, A. S., Pettinati, I., Doyle, M., Joti, Y., Owada, S., Tono, K., Batyuk, A., Hunter, M. S., Alonso-Mori, R., Bergmann, U., Owen, R. L., Sauter, N. K., Claridge, T. D. W., Robinson, C. V., Yachandra, V. K., Yano, J., Kern, J. F., Orville, A. M., and Schofield, C. J. (2021) X-ray free-electron laser studies reveal correlated motion during isopenicillin N synthase catalysis. *Sci Adv* **7**
2. Baldwin, J. E., Moroney, S. E., and Ting, H. H. (1985) A coupled enzyme assay for isopenicillin N synthetase. *Anal Biochem* **145**, 183-187
3. Jeschke, G., Timmel, C., Harmer, J. J. S., and Bonding. (2013) Structural Information from Spin-Labels and Intrinsic Paramagnetic Centres in the Biosciences. **152**, 83-120
